# Genes reveal traces of common recent demographic history for most of the Uralic-speaking populations

**DOI:** 10.1186/s13059-018-1522-1

**Published:** 2018-09-21

**Authors:** Kristiina Tambets, Bayazit Yunusbayev, Georgi Hudjashov, Anne-Mai Ilumäe, Siiri Rootsi, Terhi Honkola, Outi Vesakoski, Quentin Atkinson, Pontus Skoglund, Alena Kushniarevich, Sergey Litvinov, Maere Reidla, Ene Metspalu, Lehti Saag, Timo Rantanen, Monika Karmin, Jüri Parik, Sergey I. Zhadanov, Marina Gubina, Larisa D. Damba, Marina Bermisheva, Tuuli Reisberg, Khadizhat Dibirova, Irina Evseeva, Mari Nelis, Janis Klovins, Andres Metspalu, Tõnu Esko, Oleg Balanovsky, Elena Balanovska, Elza K. Khusnutdinova, Ludmila P. Osipova, Mikhail Voevoda, Richard Villems, Toomas Kivisild, Mait Metspalu

**Affiliations:** 1 Estonian Biocentre, Institute of Genomics, University of Tartu, Riia 23b, 51010 Tartu, Estonia; 2 Ufa Scientific Center of RAS, Ufa, 450054 Russia; 3 Statistics and Bioinformatics Group, Institute of Fundamental Sciences, Massey University, Palmerston North, 4442 New Zealand; 4 Department of Biology, University of Turku, 20014 Turku, Finland; 5 Institute of Estonian and General Linguistics, University of Tartu, 51014 Tartu, Estonia; 6 School of Psychology, University of Auckland, Auckland, 1142 New Zealand; 7 Department of Linguistic and Cultural Evolution, Max Planck Institute for the Science of Human History, D-07745 Jena, Germany; 8 The Francis Crick Institute, 1 Midland Road, London, NW1 1AT UK; 9 Institute of Genetics and Cytology of the National Academy of Sciences of Belarus, Minsk, 220072 Republic of Belarus; 10 Institute of Biochemistry and Genetics, Ufa Scientific Center of RAS, Ufa, 450054 Russia; 11 Department of Evolutionary Biology, Institute of Molecular and Cell Biology, University of Tartu, 51010 Tartu, Estonia; 12 Department of Geography and Geology, University of Turku, 20014 Turku, Finland; 13 Department of Radiology, The Mount Sinai Medical Center, New York, NY 10029 USA; 14 Institute of Cytology and Genetics, Siberian Branch of RAS, Novosibirsk, 630090 Russia; 15 Research Institute of Medical and Social Problems and Control of the Healthcare Department of Tuva Republic, Kyzyl, 667003 Russia; 16 Research Centre for Medical Genetics, Russian Academy of Medical Sciences, Moscow, 115478 Russia; 17 Northern State Medical University, Arkhangelsk, 163000 Russia; 18 Anthony Nolan, London, NW3 2NU UK; 19 Research Centre of Estonian Genome Center, Institute of Genomics, University of Tartu, 51010 Tartu, Estonia; 20 Latvian Biomedical Research and Study Centre, Riga, LV-1067 Latvia; 21 Vavilov Institute for General Genetics, RAS, Moscow, 119991 Russia; 22 Department of Genetics and Fundamental Medicine, Bashkir State University, Ufa, 450054 Russia; 23 Novosibirsk State University, 2 Pirogova Str, Novosibirsk, 630090 Russia; 24 Institute of Internal Medicine, Siberian Branch of Russian Academy of Medical Sciences, Novosibirsk, 630090 Russia; 25 Department of Archaeology, University of Cambridge, Cambridge, CB2 1QH UK; 26 Department of Human Genetics, KU Leuven, Leuven, 3000 Belgium

**Keywords:** Population genetics, Genome-wide analysis, Haplotype analysis, IBD-segments, Uralic languages

## Abstract

**Background:**

The genetic origins of Uralic speakers from across a vast territory in the temperate zone of North Eurasia have remained elusive. Previous studies have shown contrasting proportions of Eastern and Western Eurasian ancestry in their mitochondrial and Y chromosomal gene pools. While the maternal lineages reflect by and large the geographic background of a given Uralic-speaking population, the frequency of Y chromosomes of Eastern Eurasian origin is distinctively high among European Uralic speakers. The autosomal variation of Uralic speakers, however, has not yet been studied comprehensively.

**Results:**

Here, we present a genome-wide analysis of 15 Uralic-speaking populations which cover all main groups of the linguistic family. We show that contemporary Uralic speakers are genetically very similar to their local geographical neighbours. However, when studying relationships among geographically distant populations, we find that most of the Uralic speakers and some of their neighbours share a genetic component of possibly Siberian origin. Additionally, we show that most Uralic speakers share significantly more genomic segments identity-by-descent with each other than with geographically equidistant speakers of other languages. We find that correlated genome-wide genetic and lexical distances among Uralic speakers suggest co-dispersion of genes and languages. Yet, we do not find long-range genetic ties between Estonians and Hungarians with their linguistic sisters that would distinguish them from their non-Uralic-speaking neighbours.

**Conclusions:**

We show that most Uralic speakers share a distinct ancestry component of likely Siberian origin, which suggests that the spread of Uralic languages involved at least some demic component.

**Electronic supplementary material:**

The online version of this article (10.1186/s13059-018-1522-1) contains supplementary material, which is available to authorized users.

## Background

The linguistic landscape of North Eurasia is dominated by three language families—Turkic, Indo-European (IE) and Uralic. It has recently been shown that the spread of Turkic languages was mediated by gene flow from South Siberia [1]. Similarly, ancient DNA evidence of a major episode of gene flow from the Ponto-Caspian Steppe Belt to Central Europe and Central Asia during the Late Neolithic and Early Bronze Age (BA) has been interpreted as supporting the ‘Steppe Hypothesis’ of the spread of IE languages [2, 3]. However, while historical linguists have some level of consensus over the origin and spread of the Uralic languages and archaeologists have views about the dynamics of material culture over the relevant time and space [4–8], the genetic history of Uralic-speaking populations has remained poorly known.

**Fig. 1:**
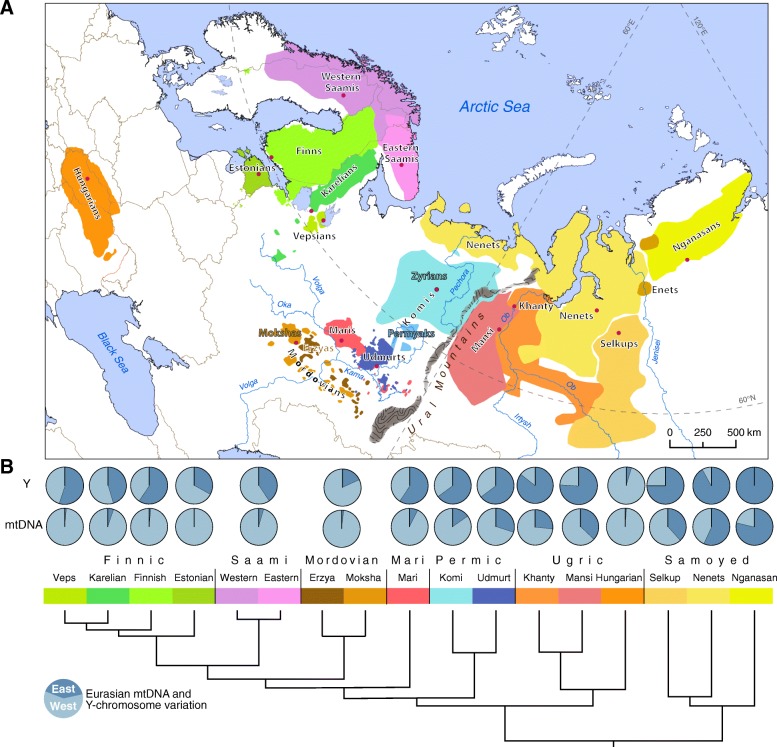
Geographic distribution of the Uralic-speaking populations and the schematic tree of the Uralic languages. **a** The geographic spread of the Uralic-speaking populations. Colour coding corresponds to the respective language in panel **b**. **b** Schematic representation of the phylogeny of the Uralic languages. Pie diagrams indicate the relative share of West and East Eurasian mitochondrial (mtDNA) and Y chromosomal (Y) lineages. Data from Additional file 5: Table S4 and Additional file 6: Table S5

The Uralic family contains 40–50 different languages [9–11] and covers a vast territory mainly from the shores of the Baltic Sea in Europe to the West Siberian Plain and the Taymyr Peninsula in Asia (Fig. 1a). According to the classical view, the Uralic languages derive from a protolanguage that split into two major branches—the Finno-Ugric (FU) and the Samoyed. The suggested age of the Uralic language family is 6,000–4,000 years before present (BP) (see e.g. [12–14], cf. [15, 16]). The most widely accepted hypotheses place the homeland of the Uralic language family into the watershed of river Volga and its tributaries Oka and Kama (see e.g. [17–20] and references therein), while some scholars propose a Siberian homeland [12, 21, 22]. The precursors of present-day FU languages gradually spread west towards the Baltic Sea (Proto-Finnic) [13, 23], north-west (Proto-Saami) [24], north (Proto-Permian branch giving rise to Komi) [9], whereas some (Udmurt, Mordovian and Mari) remained in the Volga area. The precursors of the Ugric (Khanty, Mansi and Hungarian) and Samoyed languages (e.g. Nenets, Nganasan, Selkup), spoken today mostly to the east of the Ural Mountains, but also in Central Europe (Hungarian) and in Northeast (NE) Europe (Nenets), are thought to have descended from the easternmost varieties  of the Uralic proto-languages spoken in western [18] or eastern [12] side of the Ural Mountains. Their geographic range expansion occurred most likely as a combination of demic and cultural dispersal processes [9]. The proto-Hungarian spread southwest towards Central Europe during the first millennium AD, while the linguistic ancestors of Mansi and Khanty remained mostly in West Siberia [9]. The Samoyed languages reached the periphery of their present-day spread area in the Taimyr Peninsula as late as on sixteenth century AD [25, 26]. Recent linguistic studies associate the diversification of the Uralic family with climatic and cultural changes [13] that may have led to a considerable demographic changes since the Mesolithic times in their core areas and to further migrations towards the north and northwest.

The question as which material cultures may have co-spread together with proto-Uralic and Uralic languages depends on the time estimates of the splits in the Uralic language tree. Deeper age estimates (6,000 BP) of the Uralic language tree suggest a connection between the spread of FU languages from the Volga River basin towards the Baltic Sea either with the expansion of the Neolithic culture of Combed Ware, e.g. [6, 7, 17, 26] or with the Neolithic Volosovo culture [7]. Younger age estimates support a link between the westward dispersion of Proto-Finno-Saamic and eastward dispersion of Proto-Samoyedic with a BA Sejma-Turbino (ST) cultural complex [14, 18, 27, 28] that mediated the diffusion of specific metal tools and weapons from the Altai Mountains over the Urals to Northern Europe or with the Netted Ware culture [23], which succeeded Volosovo culture in the west. It has been suggested that Proto-Uralic may have even served as the lingua franca of the merchants involved in the ST phenomenon [18]. All these scenarios imply that material culture of the Baltic Sea area in Europe was influenced by cultures spreading westward from the periphery of Europe and/or Siberia. Whether these dispersals involved the spread of both languages and people remains so far largely unknown.

Previous genetic studies have shown that demographic histories of Uralic-speaking populations inferred from maternally inherited mitochondrial (mtDNA) and paternally inherited Y chromosomes (chrY) are different. MtDNA studies of Uralic speakers suggest that the distribution of Western and Eastern Eurasian components is mostly determined by geography [29–32]. Thus, Western and Eastern Eurasian mtDNA lineages co-occur only in their contact zone in the Circum-Uralic region [29, 31, 33]. In contrast, the spread of paternal lineages among Uralic speakers in Europe does not follow this pattern: up to one half of males belong to the pan-North Eurasian chrY haplogroup (hg) N3a, which is closely related to lineages found in Siberian and East Asian populations [34–36]. This hg is virtually absent or rare in Southern Europe and in IE-speaking Scandinavians [30, 35, 37–42]. A recent study suggests that the high frequency of N3a lineages in Eastern and Northern Europe is due to a demic expansion from East Eurasia within the last 5000 years [35, 36]. It has also been suggested that certain hg N3a3`6 sub-branches may have co-spread with ST tools and possibly also FU languages [36].

Our goal in this study was to test whether the Uralic-speaking peoples share recent common genetic ancestry in their genomes. Specifically, we tested whether the clear signal of migration between East Eurasia and Europe that is present in the distribution of paternal lineages could be also detected in the patterns of autosomal variation. It has been shown earlier that the genetic landscape of northern and northeastern European populations displays affinities with Siberia [43–45] and today the components of East Eurasian origin are seen most prominently among the Fennoscandian Saami [46, 47], where they constitute about 13% of their genomes [47]. To this end, we generated a dataset of genome-wide genetic variation at over half a million genomic positions (Additional file 1: Table S1) for 15 Uralic-speaking populations (Additional file 2: Table S2), covering the main groups of the language family. We analysed this dataset in the context of relevant European and Asian populations.

## Results

### The population structure of Uralic speakers

**Fig. 2:**
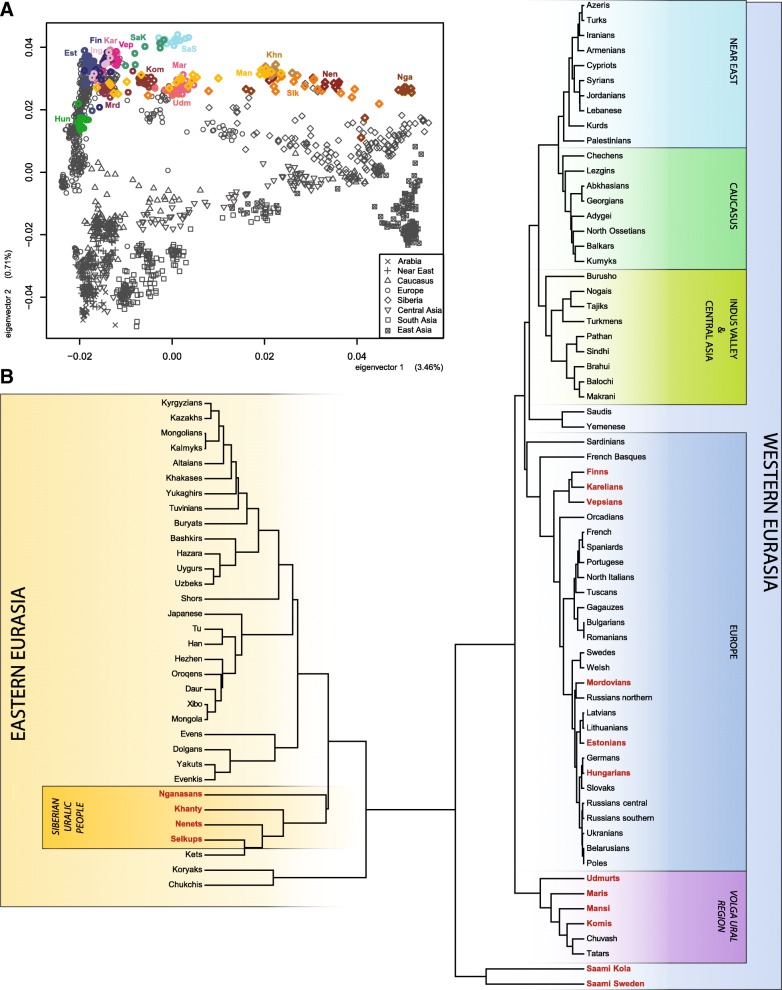
Principal component analysis (PCA) and genetic distances of Uralic-speaking populations. **a** PCA (PC1 vs PC2) of the Uralic-speaking populations (highlighted, population abbreviations are as in Additional file 1: Table S1). Values in brackets along the axes indicate the proportion of genetic variation explained by the components. **b** UPGMA tree of *F*_ST_ distances calculated based on autosomal genetic variation

To contextualize the autosomal genetic diversity of Uralic speakers among other Eurasian populations (Additional file 1: Table S1), we first ran the principal component (PC) analysis (Fig. 2a, Additional file 3: Figure S1). The first two PCs (Fig. 2a, Additional file 3: Figure S1A) sketch the geography of the Eurasian populations along the East-West and North-South axes, respectively. The Uralic speakers, along with other populations speaking Slavic and Turkic languages, are scattered along the first PC axis in agreement with their geographic distribution (Figs. 1 and 2a) suggesting that geography is the main predictor of genetic affinity among the groups in the given area. Secondly, in support of this, we find that *F*_ST_-distances between populations (Additional file 3: Figure S2) decay in correlation with geographical distance (Pearson’s *r* = 0.77, *p* < 0.0001). On the UPGMA tree based on these *F*_ST_-distances (Fig. 2b), the Uralic speakers cluster into several different groups close to their geographic neighbours.

**Fig. 3:**
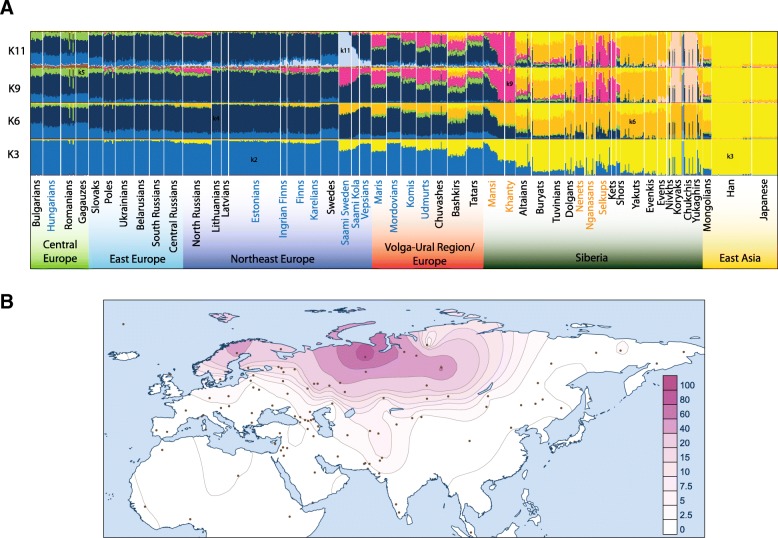
Population structure of Uralic-speaking populations inferred from ADMIXTURE analysis on autosomal SNPs in Eurasian context. **a** Individual ancestry estimates for populations of interest for selected number of assumed ancestral populations (K3, K6, K9, K11). Ancestry components discussed in a main text (k2, k3, k5, k6, k9, k11) are indicated and have the same colours throughout. The names of the Uralic-speaking populations are indicated with blue (Finno-Ugric) or orange (Samoyedic). The full bar plot is presented in Additional file 3: Figure S3. **b** Frequency map of component k9

We next used ADMIXTURE [48], which presents the individuals as composed of inferred genetic components in proportions that maximize Hardy-Weinberg and linkage equilibrium in the overall sample (see the ‘Methods’ section for choice of presented *K*). Overall, and specifically at lower values of *K*, the genetic makeup of Uralic speakers resembles that of their geographic neighbours. The Saami and (a subset of) the Mansi serve as exceptions to that pattern being more similar to geographically more distant populations (Fig. 3a, Additional file 3: S3). However, starting from *K* = 9, ADMIXTURE identifies a genetic component (k9, magenta in Fig. 3a, Additional file 3: S3), which is predominantly, although not exclusively, found in Uralic speakers. This component is also well visible on *K* = 10, which has the best cross-validation index among all tests (Additional file 3: S3B). The spatial distribution of this component (Fig. 3b) shows a frequency peak among Ob-Ugric and Samoyed speakers as well as among neighbouring Kets (Fig. 3a). The proportion of k9 decreases rapidly from West Siberia towards east, south and west, constituting on average 40% of the genetic ancestry of FU speakers in Volga-Ural region (VUR) and 20% in their Turkic-speaking neighbours (Bashkirs, Tatars, Chuvashes; Fig. 3a). The proportion of this component among the Saami in Northern Scandinavia is again similar to that of the VUR FU speakers, which is exceptional in the geographic context. It is also notable that North Russians, sampled from near the White Sea, differ from other Russians by sporting higher proportions of k9 (10–15%), which is similar to the values we observe in their Finnic-speaking neighbours. Notably, Estonians and Hungarians, who are geographically the westernmost Uralic speakers, virtually lack the k9 cluster membership.

The mitochondrial gene pool of most of the Uralic speakers is comprised of typical West Eurasian mtDNAs (Fig. 1b). Only West Siberian Nenets and Nganasans have > 50% of Eastern Eurasian mtDNA variants (Additional file 4: Table S3 and Additional file 5: Table S4). Contrary to that, a considerable amount of the chrY lineages of both West Siberian and European Uralic speakers belong to East Eurasian hg N (Fig. 1b, Additional file 6: Table S5). The only exceptions to this pattern among the Uralic speakers are Hungarians and Selkups. Among Hungarians, hg N is virtually absent, while among Selkups, it is much less frequent (< 10%) than in other Samoyeds (Additional file 6: Table S5). The prevailing hg in the paternal pool of Selkups is hg Q, which they share with genetically similar Kets and South Siberians (Additional file 3: Figure S11). Besides low frequencies of hg N Selkups share with East European populations also hg R1a-M458 (Additional file 6: Table S5). We performed correlation analysis to formally test whether the distribution of the k9 component (Fig. 3b) spatially overlaps with the spread of specific chrY hg N2 and N3 lineages that have been shown to be relevant in the context of Uralic speakers [36]. We found a weak but significant correlation with the sub-hgs spread near the Ural Mountains, but not with those that reach up to Fennoscandia (Additional file 7: Table S6).

We also tested the different demographic histories of female and male lineages by comparing outgroup *f*3 results for autosomal and X chromosome (chrX) data for pairs of populations (Estonians, Udmurts or Khanty vs others) with high versus low probability to share their patrilineal ancestry in chrY hg N (see the ‘Methods’ section, Additional file 3: Figure S13). We found a minor but significant excess of autosomal affinity relative to chrX for pairs of populations that showed a higher than 10% chance of two randomly sampled males across the two groups sharing their chrY ancestry in hg N3-M178, compared to pairs of populations where such probability is lower than 5% (Additional file 3: Figure S13).

In sum, these results suggest that most of the Uralic speakers may indeed share some level of genetic continuity via k9, which, however, also extends to the geographically close Turkic speakers.

### Distilling the language-mediated excess of genetic continuity

To test whether the common genetic substrate of Uralic speakers suggested by the k9 component also presents itself in the sharing patterns of derived alleles across the genome, we calculated *D*-statistics [49] as in Skoglund et al. [50] (Additional file 3: Figure S4). We explored derived allele sharing patterns in a wide set of Eurasian populations contrasting sharing with the westernmost Uralic speakers (Saami, Finns, Estonians, Hungarians) on one hand and European populations (Swedes, Poles, French) on the other. We found that it is the admixture with the Siberians that makes the Western Uralic speakers different from the tested European populations (Additional file 3: Figure S4A-F, H, J, L). Differentiating between Estonians and Finns, the Siberians share more derived alleles with Finns, while the geographic neighbours of Estonians (and Finns) share more alleles with Estonians (Additional file 3: Figure S4M). Importantly, Estonians do not share more derived alleles with other Finnic, Saami, VUR FU or Ob-Ugric-speaking populations than Latvians (Additional file 3: Figure S4O). The difference between Estonians and Latvians is instead manifested through significantly higher levels of shared drift between Estonians and Siberians on the one hand and Latvians and their immediate geographic neighbours on the other hand. None of the Uralic speakers, including linguistically close Khanty and Mansi, show significantly closer affinities to the Hungarians than any non-FU population from NE Europe (Additional file 3: Figure S4R).

**Fig. 4:**
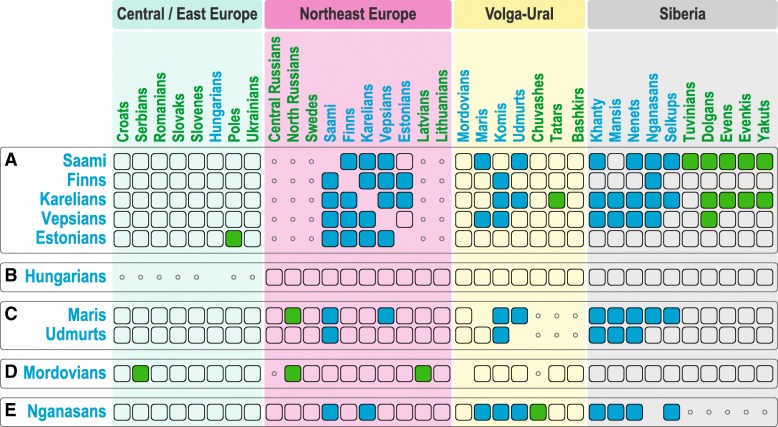
Share of ~ 1–2 cM identity-by-descent (IBD) segments within and between regional groups of Uralic speakers. For each Uralic-speaking population representing lines in this matrix, we performed permutation test to estimate if it shows higher IBD segment sharing with other population (listed in columns) as compared to their geographic control group. Empty rectangles indicate no excess IBD sharing, rectangles filled in blue indicate comparisons when statistically significant excess IBD sharing was detected between one Uralic-speaking population with another Uralic-speaking population (listed in columns), rectangles filled in green mark the comparisons when a Uralic-speaking population shows excess IBD sharing with a non-Uralic-speaking population. For each tested Uralic speaker (matrix rows) populations in the control group that were used to generate permuted samples are indicated using small circles. For example, the rectangle filled in blue for Vepsians and Komis (A) implies that the Uralic-speaking Vepsians share more IBD segments with the Uralic-speaking Komis than the geographic control group for Vepsians, i.e. populations indicated with small circles (Central and North Russians, Swedes, Latvians and Lithuanians). The rectangle filled in green for Vepsians and Dolgans shows that the Uralic-speaking Vepsians share more IBD segments with the non-Uralic-speaking Dolgans than the geographic control group

We next tested whether the Uralic-speaking populations share more identity-by-descent (IBD) [51] segments with geographically distant Uralic groups than their non-Uralic neighbours do (Fig. 4 A–E, Additional file 8: Table S7, Additional file 9: Table S8, Additional file 10: Table S9). High IBD sharing between Permic speakers and Khanty has been earlier reported in Triska et al. [52]. Indeed, Finnic speakers and Saami share more IBD segments with their distant linguistic relatives in VUR (Mari, Komi and Udmurts) and even with West Siberian Uralic speakers than NE Europeans in the control group (blue cells, Fig. 4 A). In addition, Saami and Karelians show a significant excess of IBD segment sharing with several non-Uralic peoples of Siberia (green cells, Fig. 4 A). Compared to their non-Uralic neighbours, the Samoyedic Nganasans share more IBD segments with all the tested Siberian Uralic speakers, most of the Uralic speakers from VUR, and even with Saami and Karelians from NE Europe (Fig. 4 E). When Maris and Udmurts (Fig. 4 C) are compared to their neighbouring Chuvashes, Tatars and Bashkirs, they display more shared IBD segments with Saami, Vepsians and North Russians in the west and specifically only with the Uralic-speaking populations to the east of the Ural Mountains. All the above-mentioned findings of IBD analyses attest to at least some degree of common genetic substrate among most of the analysed Uralic populations. Yet, we did not find any excess IBD sharing when Estonians (Fig. 4 A), Hungarians (Fig. 4 B) and Mordovians (Fig. 4 D) were compared to the Uralic speakers from VUR and Siberia.

#### fineSTRUCTURE. Globetrotter

To study the fine-scale genetic structure of Uralic-speaking populations and to reconstruct past admixture events in their history, we used the haplotype-based approach implemented in fineSTRUCTURE [53] and GLOBETROTTER [54]. fineSTRUCTURE clusters individuals into natural groups based on patterns of haplotype sharing similarity. The clusters identified in our sample largely correspond to self-identified ethnic groups while higher hierarchical clusters follow broader geographic proximity patterns (Additional file 3: Figures S5-S6). In some cases, geographic and linguistic proximity co-vary and for example the clusters we name ‘Finnic’ and ‘Saami’ (Additional file 3: Figure S5) consist of only Uralic speakers, while ‘Europe 1’ and ‘Europe 2’ encompass both Uralic and non-Uralic speakers. Here, Uralic-speaking Hungarians group together with Slavic speakers and Germanic-speaking Swedes in ‘Europe 2’ (Additional file 3: Figure S5). Similarly, Uralic-speaking Estonians form a cluster with Baltic-speaking Latvians and Lithuanians (‘Europe 1’ in Additional file 3: Figure S5), which also includes Mordovians and Russians.

##### Globetrotter full analysis

**Fig. 5:**
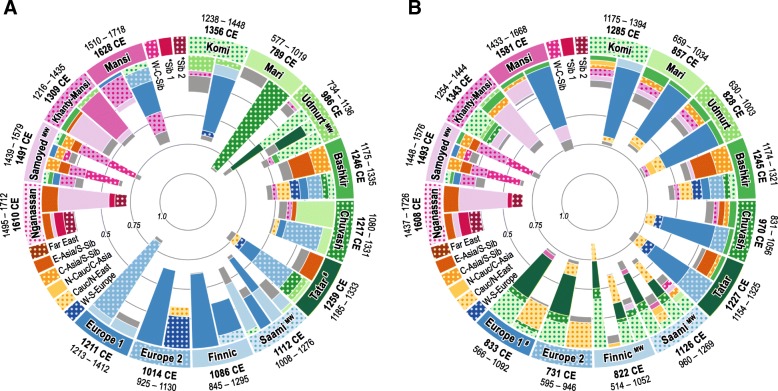
Circos plots of GLOBETROTTER (GT) results. The outer circle represents target groups for which GT inference was performed (wide segments) and additional surrogate populations, which were used to describe admixture in target populations (narrow segments). Geographic affiliation of target groups is colour-coded: blue—Europe (except populations from Volga-Ural region—Komis, Udmurts, Maris, Tatars, Chuvashes, Bashkirs); green—Volga-Ural region; and magenta—Western Siberia. Inner bar plots depict genetic composition of inferred sources of admixture in each of the target groups. A pair of sources is shown for a simple one-way admixture event between two populations, and an additional pair of sources for the less strongly signaled event is shown for a one-date multi-way admixture between more than two sources (marked as MW in the outer circle). In a simple one-date event, a pair of sources contributes 100% of the DNA of the target population. Surrogate populations in the inner bar plots are shaded according to the colour scheme given in the outer ring, and those contributing < 3% to mixing sources are coloured in grey. Point estimates and confidence intervals for the date of inferred admixture event are shown next to the cluster label. The details of the GT source groups are given in Additional file 3: Figure S5 and Additional file 11: Table S10. **a** Results of ‘full’ analysis, where each cluster was allowed to copy from every other cluster. **b** Results of ‘regional’ analysis, where no copying between samples from the same geographical region was allowed. For example, in the ‘full’ analysis of the ‘Europe 1’ cluster, a simple one-date admixture event was detected. The first source population contributes 85% of the total DNA, including 76% from the ‘Europe 2’ surrogate; the second source contributes 15% and is dominated by the ‘Finnic’ cluster. The admixture took place around 1211 CE (95% CI: 1213–1412 CE). Abbreviations: C-Central; Cauc-Caucasus; E-East; N-North; S-South; Sib-Siberia; W-West.

The natural clusters defined by fineSTRUCTURE were further used to study admixture history with Globetrotter analysis, which was applied in two different setups. First, we performed the ‘full’ analysis, where every recipient population could copy from any other donor group (Fig. 5a, Additional file 11: Table S10). Populations of interest were clustered into three geographically defined groups: (1) European (blue palette, all studied Europeans except the easternmost VUR populations—Maris, Udmurts, Komis, Tatars, Bashkirs and Chuvashes); (2) VUR (green palette); and (3) West Siberian (magenta palette). Most of the inferred admixture events in this analysis were simple one-date events with high statistical support (third quartile of maxR2 fit scores of single date events = 0.91, Additional file 11, Table S10). As expected, many events involved contacts between geographically close source populations. For example, the majority of admixture events in the European set (‘Europe 1’, ‘Europe 2’, ‘Finnic’ and ‘Saami’, Fig. 5a) involve populations from within the set, and only two groups show traces of admixture from non-European sources: ‘Europe 2’ from Caucasus/Near Eastern groups and ‘Saami’ from the VUR (Fig. 5).

In the VUR (Fig. 5a), all populations except Maris and Udmurts show considerable admixture with other Europeans. In West Siberia, three out of four clusters display traces of admixture mostly with Siberian, and East and Central Asian donors. On the contrary, ‘Mansi’ has a distinctive admixture profile—nine Mansi samples cluster closely together with VUR populations (Additional file 3: Figure S5; for ADMIXTURE profiles, see also Fig. 3) and, similarly to them, show evidence for substantial recent admixture with Europeans around sixteenth to eighteenth century. This group is considered as an outlier here. The rest of the Mansi are clustered with Khanty people following the linguistic grouping of Ob-Ugric (Additional file 3: Figure S5).

##### Globetrotter regional analysis

The excess of admixture events between closely related geographical neighbours may mask traces of subtle genetic contacts with distantly related populations [54]. Therefore, in the ‘regional’ analysis, we excluded neighbours from the set of possible surrogates, allowing copying only from donors with a different group label, if not specified differently (see Additional file 12: Table S11 and note therein). Similarly to the ‘full’ analysis, ‘regional’ results have high statistical support (third quartile of maxR2 fit scores of single date events = 0.93, Additional file 11: Table S10).

In the European set, only the Uralic-speaking ‘Finnic’ and ‘Saami’ clusters have a detectable (more than 3%) amount of admixture with West Siberian sources (Fig. 5b), even if we leave aside the contribution from already admixed Western Siberian ‘Mansi’ cluster (Fig. 5 and Additional file 3: Figure S5). In ‘Saami’, the Siberian influence is more notable as well as diverse: it is linked both with ‘Samoyed’ (consisting here of Nenets, Selkups and neighbouring Kets) and with West/Central Siberian (‘W-C-Sib’) clusters (Fig. 5b).

The Uralic- and non-Uralic clusters from the VUR have different admixture histories. Turkic speakers (Bashkirs, Tatars and Chuvashes) contain three European donors (‘Europe1’, ‘Europe2’ and ‘W-S-Europe’; Fig. 5b), while admixture in Uralic speakers displays mostly only one dominating European Eastern Baltic/Russian surrogate (‘Europe1’). In addition, Turkic speakers receive substantial genetic contribution from South Siberian/East Asian groups (‘E-Asia/S-Sib’ in Fig. 5b), as was also shown earlier in Yunusbaev et al. [1]. This is not seen in the Uralic-speaking groups (Komis, Maris, Udmurts), who instead have both ‘Khanty-Mansi’ and ‘Samoyed’, i.e. Uralic-speaking Siberian donors. Contacts between Uralic speakers from Europe and West Siberia/VUR display mostly unidirectional east-to-west ‘donating’ pattern: for example, Komis are dominant surrogates for the ‘Finnic’ and ‘Saami’ groups, but the latter two do not contribute much to admixture events involving Komis. A similar trend was also seen in IBD analysis (Fig. 4).

In the ‘regional’ analysis, admixture sources of West Siberian ‘Khanty-Mansi’ include Samoyeds and a range of VUR surrogates with a minor Central Asian/South Siberian component (‘C-Asia/S-Sib’). The ‘Samoyed’ cluster shows evidence for a complex one-date multiway admixture shaped by multiple regionally diverse surrogates dominated by West/Central Siberian and Khanty-Mansi groups. The ‘Samoyed’ cluster is, together with South and West Siberians, also a major contributor to an admixture event in a separate Samoyed-speaking group—Nganasans (‘Nganassan’, Fig. 5b and Additional file 3: Figure S6, see Additional file 12: Table S11 for details). The most distinct difference between Ob-Ugric (Khanty-Mansi) and Samoyedic speakers (Nenets, Selkups and Nganasans) is the presence of East Asian/South Siberian (‘E-Asia/S-Sib’, Fig. 5) component in the latter.

The time depth of the Globetrotter (Fig. 5b) inferred admixture events is relatively recent—500–1900 AD (see also complementary ALDER results, in Additional file 13: Table S12 and Additional file 3: Figure S7)—and agrees broadly with the results reported in Busby et al. [55]. A more detailed examination of the ALDER dates, however, reveals an interesting pattern. The admixture events detected in the Baltic Sea region and VUR Uralic speakers are the oldest (800–900 AD or older) followed by those in VUR Turkic speakers (∼1200–1300 AD), while the admixture dates for most of the Siberian populations (>1500 AD) are the most recent (Additional file 3: Figure S7). The West Eurasian influx into West Siberia seen in modern genomes was thus very recent, while the East Eurasian influx into NE Europe seems to have taken place within the first millennium AD (Fig. 5b, Additional file 3: Figure S7).

### Affinities of the Uralic speakers with ancient Eurasians

**Fig. 6:**
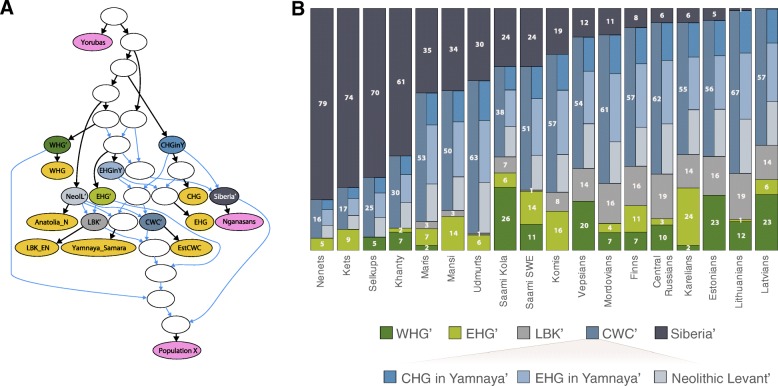
Proportions of ancestral components in studied European and Siberian populations and the tested qpGraph model. **a** The qpGraph model fitting the data for the tested populations. Colour codes for the terminal nodes: pink—modern populations (‘Population X’ refers to test population) and yellow—ancient populations (aDNA samples and their pools). Nodes coloured other than pink or yellow are hypothetical intermediate populations. We putatively named nodes which we used as admixture sources using the main recipient among known populations. The colours of intermediate nodes on the qpGraph model match those on the admixture proportions panel. **b** Admixture proportions (%) of ancestral components. We calculated the admixture proportions summing up the relative shares of a set of intermediate populations to explain the full spectrum of admixture components in the test population. We further did the same for the intermediate node CWC’ and present the proportions of the mixing three components in the stacked column bar of CWC’. Colour codes for ancestral components are as follows: dark green—Western hunter gatherer (WHG’); light green—Eastern hunter gatherer (EHG’); grey—European early farmer (LBK’); dark blue—carriers of Corded Ware culture (CWC’); and dark grey—Siberian. CWC’ consists of three sub-components: blue—Caucasian hunter-gatherer in Yamnaya (CHGinY’); light blue—Eastern hunter-gatherer in Yamnaya (EHGinY’); and light grey—Neolithic Levant (NeolL’)

We next calculated outgroup *f*3-statistics [48] to estimate the extent of shared genetic drift between modern and ancient Eurasians (Additional file 14: Table S13, Additional file 3: Figures S8-S9). Consistent with previous reports [45, 50], we find that the NE European populations including the Uralic speakers share more drift with any European Mesolithic hunter-gatherer group than Central or Western Europeans (Additional file 3: Figure S9A-C). Contrasting the genetic contribution of western hunter-gatherers (WHG) and eastern hunter-gatherers (EHG), we find that VUR Uralic speakers and the Saami share more drift with EHG. Conversely, WHG shares more drift with the Finnic and West European populations (Additional file 3: Figure S9A). Interestingly, we see a similar pattern of excess of shared drift between VUR and EHG if we substitute WHG with the aDNA sample from the Yamnaya culture (Additional file 3: Figure S9D). As reported before [2, 45], the genetic contribution of European early farmers decreases along an axis from Southern Europe towards the Ural Mountains (Fig. 6, Additional file 3: Figure S9E-F).

We then used the *qpGraph* software [48] to test alternative demographic scenarios by trying to fit the genetic diversity observed in a range of the extant Finno-Ugric populations through a model involving the four basic European ancestral components: WHG, EHG, early farmers (LBK), steppe people of Yamnaya/Corded Ware culture (CWC) and a Siberian component (Fig. 6, Additional file 3: Figure S10). We chose the modern Nganasans to serve as a proxy for the latter component because we see least evidence for Western Eurasian admixture (Additional file 3: Figure S3) among them. We also tested the Khantys for that proxy but the model did not fit (yielding *f*2-statistics, *Z*-score > 3). The only Uralic-speaking population that did not fit into the tested model with five ancestral components were Hungarians. The *qpGraph* estimates of the contributions from the Siberian component show that it is the main ancestry component in the West Siberian Uralic speakers and constitutes up to one third of the genomes of modern VUR and the Saami (Fig. 6). It drops, however, to less than 10% in most of NE Europe, to 5% in Estonians and close to zero in Latvians and Lithuanians. Indeed, Estonians show an excess of shared derived alleles with Nganasans compared to Latvians [*D*-statistic of the form D(Yorubas, Nganasans; Estonians, Latvians) = − 0.00263 (± 0.0008); *Z*-score = − 3.0691)] and Lithuanians [D(Yorubas, Nganasans; Estonians, Lithuanians) = − 0.00426 (± 0.0009); *Z*-score = − 5.6638)].

### Correlation between linguistic, geographical and genetic data of Uralic speakers

In order to determine whether and to what extent Uralic linguistic ancestry predicts genetic ancestry (see Additional file 3: Figure S12), we studied the correlations of genetic (autosomal, mtDNA and chrY, Additional file 15: Table S14A-F), linguistic (Additional file 15: Table S14G) and geographical distances (Additional file 15: Table S14H) with Mantel [56] and partial Mantel tests [57] (see the ‘Methods’ section for details). We used two types of autosomal distance matrices: *F*_ST_ distances and the fineSTRUCTURE coancestry based matrix using the data of shared chunk counts and two types of *F*_ST_s with both mtDNA and chrY (six genetic distance matrices in total, Additional file 15: Table S14).

Lexical distances between Uralic languages were significantly positively correlated with all types of genetic distances (Additional file 16: Table S15). Lexical distances also increased with geographical distances (*r* = 0.62, *p* = 0.001) as did all the genetic distances (Additional file 16: Table S15). When the effect of geographical distance was taken into account, lexical and autosomal distances still showed significant connections. For the fineSTRUCTURE-based distances, the correlation was twice stronger than for *F*_ST_-based distances (*r* = 0.46, *p* = 0.001 vs *r* = 0.25, *p* = 0.01). This is consistent with the expectation that haplotype-based distances capture more recent signals of shared ancestry that are more relevant to recent history of language expansions. For mtDNA and chrY distances, correlation was not significant after correcting for geography. Thus, our findings indicate a clear relationship between autosomal genetic distances and lexical distances among Uralic-speaking populations, even when the effect of geographical distance is taken into account. The non-significant finding with respect to mtDNA and chrY data may reflect greater noise in these haploid loci. It is also worth noting that geographical distances significantly predict autosomal and chrY distances (but do not predict mtDNA distances) when keeping the lexical distance constant (Additional file 16: Table S15). This indicates that while lexical distance accounts for some of the variation in autosomal genetic distances between populations independent of geography, there remains genetic variation between groups that is attributable to geography independent of lexical distance—i.e. genetic variation is explained by a combination of lexical distance and geographic distance.

## Discussion

In prehistoric times, with no written texts or alphabets, learning a language was only possible through direct contact and, hence, it is natural to expect that languages would often spread together with human migrations [58]. There are several examples where indeed a large-scale migration of people has also resulted in the dispersal of new languages in a previously populated area [59–61]. On the other hand, it has been shown that the spread of Turkic languages has at least in its westernmost reach been mediated by only a handful of migrants [1]. A more complex pattern of migration and admixture appears to be behind the ‘Central-East European’ supralinguistic genetic substratum characterising both East and West Slavic-IE speakers [62].

Here, we have studied the genetic variation of 15 Uralic-speaking populations to reveal patterns that could correspond to the spatial distribution of the Uralic languages. Our analyses show that in the first approximation, the genetic diversity patterns of the Uralic speakers correspond to geography. Principal component and ADMIXTURE analyses suggest that the Uralic-speaking populations are genetically more similar to their neighbours than to geographically distant linguistic relatives. These analyses capture the broad-scale patterns of genetic variation arisen through the cumulative demographic processes in the population history of the continent. Importantly, ADMIXTURE analysis suggests a genetic component (k9) that is primarily present in most Uralic speakers (Fig. 3). Assuming that the spread of Uralic languages occurred within the past 5 kya, we next focused on haplotype sharing patterns between populations to concentrate on more recent demographic events. By inferring sharing of IBD segments between populations, we found the excess of shared IBD segments between most of the Uralic speakers (Fig. 4). This pattern is most notable for Uralic speakers in the Volga River basin who share more IBD segments with Uralic speakers both to the west and to the east of them than do their geographic neighbours.

Our Mantel test is consistent with the presence of common genetic substrate in most of the Uralic speakers (Additional file 16: Table S15): we found a significant association between autosomal genes and lexical variation that is independent of geographic proximity. This could be due to the legacy of ancient migrations shaping both genetic and linguistic diversity. Alternatively, the association could reflect a bias in gene flow between close linguistic relatives. The clear relationship between genetic and linguistic data does not extend to haploid markers, possibly because the latter are more prone to the effects of genetic drift. It has to be noted that this quantitative approach does not allow us to study the correlation between the spread of specific genetic and linguistic sub-lineages, for which a more precise case studies could provide information in the future.

We next used fineSTRUCTURE and Globetrotter approach to identify genetic clusters and admixture signals based on a wider spectrum of shared haplotypes. This approach does not depend on prior information on sample groupings and operates instead with data-driven natural groups defined by patterns of haplotype sharing. Most of the Finnic, Saami and VUR Uralic speakers form clusters in accordance with their self-reported linguistic affinity. These clusters are also distinct from the neighbouring Turkic speakers who form their own groupings. The exceptions here are for example the Mansi, who clearly form two clusters that differ in the extent of recent admixture with NE Europeans.

One of the notable observations that stands out in the fineSTRUCTURE analysis is that neither Hungarians nor Estonians or Mordovians form genetic clusters with other Uralic speakers but instead do so with a broad spectrum of geographically adjacent samples. Despite the documented history of the migration of Magyars [63] and their linguistic affinity to Khantys and Mansis, who today live east of the Ural Mountains, there is nothing in the present-day gene pool of the sampled Hungarians that we could tie specifically to other Uralic speakers. It is important to note here that our sample comes from the capital region. Given the complex history and ethnic makeup of Hungary, it is possible that a comprehensive sampling of the country could reveal genetic ties to the Ugric speakers. Furthermore, analyses of early medieval aDNA samples from Karos-Eperjesszög cemeteries in Hungary have revealed the presence of mtDNA haplogroups with East Asian provenance testifying for vestiges of a real migration of people from the east [64].

Perhaps even more surprisingly, we found that Estonians, who show close affinities in IBD analysis to neighbouring Finnic speakers and Saami, do not share an excess of IBD segments with the VUR or Siberian Uralic speakers. This is even more striking considering that the immediate neighbours—Finns, Vepsians and Karelians—do. In this context, it is important to remind that the limited (5%, Fig. 6) East Eurasian impact in the autosomal gene pool of modern Estonians contrasts with the fact that more than 30% of Estonian (but not Hungarian) men carry chrY N3 that has an East Eurasian origin and is very frequent among NE European Uralic speakers [36]. However, the spread of chrY hg N3 is not language group specific as it shows similar frequencies in Baltic-speaking Latvians and Lithuanians, and in North Russians, who in all our analyses are very similar to Finnic-speakers. The latter, however, are believed to have either significantly admixed with their Uralic-speaking neighbours or have undergone a language shift from Uralic to Indo-European [38].

Saami stand out from other NE European populations by drawing up to 30% of their autosomal ancestry from Asian genetic components (Fig. 3). They also display long-range genetic affinities with both the Uralic- and non-Uralic-speaking Siberians (Figs. 4 and 5). This is probably because the ancestors of the modern Saami (a) have lived in isolation from Southern and Eastern European gene flow and (b) have had more contacts with Nordic peoples on both sides of the Ural Mountains, driven by the similar life-style of the Arctic people. Curiously enough, the mtDNA heritage of Saami can be considered as predominantly (< 90%) Western Eurasian [30].

With some exceptions such as Estonians, Hungarians and Mordovians, both IBD sharing and Globetrotter results suggest that there are detectable inter-regional haplotype sharing ties between Uralic speakers from West Siberia and VUR, and between NE European Uralic speakers and VUR. In other words, there is a fragmented pattern of haplotype sharing between populations but no unifying signal of sharing that unite all the studied Uralic speakers.

Recent aDNA studies have shown that extant European populations draw ancestry form three main migration waves during the Upper Palaeolithic, the Neolithic and Early Bronze Age [2, 3, 45]. The more detailed reconstructions concerning NE Europe up to the Corded Ware culture agree broadly with this scenario and reveal regional differences [65–67]. However, to explain the demographic history of extant NE European populations, we need to invoke a novel genetic component in Europe—the Siberian. The geographic distribution of the main part of this component is likely associated with the spread of Uralic speakers but gene flow from Siberian sources in historic and modern Uralic speakers has been more complex, as revealed also by a recent study of ancient DNA from Fennoscandia and Northwest Russia [68]. Thus, the Siberian component we introduce here is not the perfect but still the current best candidate for the genetic counterpart in the spread of Uralic languages. On the westernmost reach of Uralic speakers, the extent of this shared ancestry is, however, small on the genome level and is significantly sex-specific in its nature. The shared ancestry is clearly pronounced in chrY, with Uralic speakers showing distinctively high (29% on average) frequency of hg N3-M178. The tested Uralic-speaking populations show marginally, though significantly, higher affinity to populations with high frequency of N3-M178 in the autosomal loci than predicted from their X chromosomal similarity and their comparison to other populations where N3-M178 is infrequent or absent. These sex-specific differences which are widely spread among Uralic speakers today may trace their origins back to the time of the shared population history of the Uralic populations and reflect complex socio-cultural factors amplified by small effective population sizes, potentially including examples such as male-specific *elite dominance* and/or cultural inheritance of male reproductive fitness [34, 69] during the time of their dispersal and admixture with neighbouring groups.

Understanding the interplay between the cultural and demographic processes leading to these observations will, no doubt, motivate future studies, especially those that will be done in the field of ancient genomics.

## Conclusions

Here, we present for the first time the comparison of genome-wide genetic variation of nearly all extant Uralic-speaking populations from Europe and Siberia. We show that (1) the Uralic speakers are genetically most similar to their geographical neighbours; (2) nevertheless, most Uralic speakers along with some of their geographic neighbours share a distinct ancestry component of likely Siberian origin. Furthermore, (3) most geographically distant Uralic speaking populations share more genomic IBD segments with each other than with equidistant populations speaking other languages and (4) there is a positive correlation between linguistic and genetic data of the Uralic speakers. This suggests that the spread of the Uralic languages was at least to some degree associated with movement of people. Moreover, the discovery of the Siberian component shows that the three known major components of genetic diversity in Europe (European hunter-gatherers, early Neolithic farmers and the Early Bronze Age steppe people) are not enough to explain the extant genetic diversity in (northeast) Europe.

## Methods

### Linguistic background and geographical location of the samples

Approximately 20.5 million people speak Uralic languages today [10] (see details in Additional file 2: Table S2), and only three of the Uralic languages—Hungarian, Finnish and Estonian (Fig. 1a, Additional file 2: Table S2)—are not listed as endangered in the *UNESCO Atlas of the World’s Languages in Danger* [70]*.* In this study, DNA samples of a total of 15 Uralic-speaking populations from Europe and Western Siberia were collected from the present-day spread area of corresponding Uralic languages (Fig. 1, Additional file 2: Table S2). The population affiliations of the samples were derived from the reported self-identity of the volunteers. We assume that these affiliations reflect also the language they speak as their mother tongue. The samples used here do not encompass all extant Uralic languages, but represent examples of each of the main branches of the family, and cover the whole distribution area.

Due to the small sample sizes and genetic homogeneity revealed by the genetic profiles on the ADMIXTURE plot of some of the studied populations, we pooled the samples of Erzas and Mokshas together as Mordovians and the samples of Permyak and Zyryan Komis as Komi. The heterogeneous Mansi population was divided into two to three subsets, according to the proportions of Eastern and Western Eurasian ancestry components in their genetic profiles. The Finnish group consists of Finns and Ingrian Finns who have been analysed separately in the analyses of PCA, *F*_ST_ distance calculations, ADMIXTURE and *D*-statistics.

DNA of the samples was extracted from whole blood according to the phenol/chloroform method [71]. DNA concentrations were determined with spectrophotometry (NanoDrop, Wilmington).

A total of 286 samples of Uralic-speaking individuals, of those 121 genotyped in this study, were analysed in the context of 1514 Eurasian samples (including 14 samples published for the first time) based on whole genome single nucleotide polymorphisms (SNPs) (Additional file 1: Table S1). All these samples, together with the larger sample set of Uralic speakers, were characterized for mtDNA and chrY markers.

### Population structure and admixture

A total of 135 samples from this study were genotyped using the Illumina 610K, 650K, 660K or 1M SNPs arrays (Human610-Quad, HumanHap650Y, Human660W-Quad or HumanOmni1-Quad BeadChip DNA Analysis BeadChip Kits) and analysed for the whole-genome variation together with published genotype data (Additional file 1: Table S1). We used the software PLINK 1.05 [72] to filter the dataset and to include only SNPs of autosomal chromosomes with minor allele frequency > 0.1% and genotyping success > 97%. We excluded SNPs in strong linkage disequilibrium (LD) (pairwise genotypic correlation *r*^2^ > 0.4) in a window of 200 SNPs (sliding the window by 25 SNPs at a time), due to the possible effect of background LD on PCA and structure-like analysis. To exclude possible close relative pairs (first and second degree) among the individuals, the software KING v1.4 [73] was applied to the entire dataset and the resulting data were confirmed by REAP v.1.2 [74]. The samples (populations and no. of individuals) used for different analysis are given in Additional file 1: Table S1.

#### PCA and *F*_ST_

PCA (Fig. 2a and Additional file 3: Figure S1) was carried out with the smartpca program of the EIGENSOFT package [75], using 171,454 SNPs. Mean pairwise *F*_ST_ values between populations and regional population groups for 303,671 autosomal SNPs (Additional file 3: Figure S2) were calculated with the method of Weir and Cockerman [76] as in Metspalu et al. [77]. Only populations with *n* > 4 were included in *F*_ST_ calculations. The UPGMA tree that visualizes the clustering based on the genetic distances of studied population was built with MEGA7 [78] (Fig. 2b).

#### ADMIXTURE

The population structure was analysed using the software ADMIXTURE [79] implementing a structure-like [80] model-based maximum likelihood clustering algorithm (Fig. 3 and Additional file 3: Figure S3). The final dataset of ADMIXTURE analysis of Uralic-speaking populations in the general Eurasian background consisted of 181,005 SNPs and 1800 individuals from 111 populations (Additional file 1: Table S1). We ran ADMIXTURE 100 times for each assumed number of ancestral populations (*K*) from *K* = 3 to *K* = 20 to observe the deviation of the results between individual runs (Additional file 3: Figure S3A). According to a low level of variation in log likelihood scores (LLs < 1) within the top 10% fraction of runs with the highest LLs [81], we assume that usable results were at *K* = 3 to *K* = 18 and the best fit *K* value appears on K10 level (Additional file 3: Figure S3). We use letter *k* to refer to the specific components in the genetic profiles of individuals/populations. The frequency of k9 component from Additional file 7: Table S6B was spatially mapped (Fig. 3b) with Surfer software (v7, Golden Software, Inc.).

#### *D*-statistics

We calculated *D*-statistics [49] (Additional file 3: Figure S4A-R) from tests of four populations in the form of (A, B; C, D), where A is an outgroup, B is a test population, C is an Uralic-speaking population and D is a non-Uralic-speaking population, for a suggested tree-like population history as in Skoglund et al. [50]. The test provides information on whether or not the test population (B) shares more derived alleles with one population from a pair (C, D) than is expected from the process of incomplete lineage sorting without admixture, indicating a recent gene flow between B and C or B and D. If *D* < 0, a test population (B) shares more derived alleles with the Uralic-speaking population (C) compared to the non-Uralic-speaking population (D); if *D* > 0, a test population (B) shares more derived alleles with the non-Uralic-speaking population (D) compared to the Uralic-speaking population (C). We used Yorubas as an outgroup (A) and one out of four westernmost Uralic-speaking populations: Saami from Sweden, Finns, Estonians and Hungarians; and one out of three European non-Uralic speakers: French (representing West Europeans), Poles (representing East Europeans) and Swedes (representing North Europeans) as a fixed pair (C, D). We ran the *D*-statistics test (Additional file 3: Figure S4A-L) with a list of European and Siberian populations (Additional file 1: Table S1) used as the test population (B). We also ran the test using the local geographical neighbours of European Uralic speakers (Additional file 3: Figure S4M-R). The null hypothesis was that there is no excess share of derived alleles between the Uralic-speaking populations and test population B (*D* = 0). Only the *D*-values with |*Z*-score| > 3 were considered significant.

#### Analysis of IBD segment sharing

We used the fastIBD algorithm implemented in the BEAGLE 3.3 software [51] to detect chromosomal tracts (> 1 cM in length) that are IBD between pairs of individuals (Fig. 4, Additional file 8: Table S7, Additional file 9: Table S8, Additional file 10: Table S9). IBD tracts with a fastIBD score of 1e−10 from ten independent runs were further post-processed using the algorithm developed by Ralph and Coop 2013 [82]. This algorithm removes spurius gaps, breaks introduced into long blocks of IBD by low marker density and phasing switch-error and performs final IBD tract calls. IBD tracts were first sorted into bins (classes) based on their length: 1–2, 2–3, 3–4 and 4–5 cM. Within each bin, we computed average length of IBD (sum of all tracts divided by sample size) between randomly chosen pairs of subsamples from two populations. We then tested whether Uralic speakers from different regions demonstrate more IBD sharing between each other. For this, we split populations in our dataset into three regional groups: 1—Baltic-Scandinavian; 2—Eastern European-Volga; and 3—Siberian. We then computed average IBD sharing between Uralic speakers from two different regions. Next, within each region, for each tested Uralic-speaking population, we selected non-Uralic-speaking populations that are geographically close to them, as a control group. In order, to assess, for example, whether Finns from the Baltic-Scandinavian region have higher than expected IBD sharing with Udmurts, we compared observed IBD sharing with values characteristic of the control group populations. Namely, we took multiple random samples from the pooled set of control for Finns and computed IBD sharing with Udmurts and compared it with the observed value. Given no recent shared ancestry between Finns and Udmurts due to linguistic relatedness, Finns are expected to show the same level of IBD sharing as their control group. IBD sharing values higher than background were counted to compute a *p* value. We note that for some populations, we do not have an appropriate geographic control group to carry out this kind of permutation test. Nevertheless, most of the Uralic peoples tested show higher IBD sharing with distant Uralic speakers compared to their regional non-Uralic control, and this suggests higher number of shared ancestors between Uralic speakers within the past dozens of generations.

#### fineSTRUCTURE and GLOBETROTTER

While ADMIXTURE uses independent unlinked SNPs for reconstructing individual ancestries, fineSTRUCTURE is a much more powerful approach which infers fine-scale population structure from haplotype data. Each individual is presented as a matrix of non-recombining genomic chunks received from a set of multiple donor individuals. The patterns of similarities between these copying matrices are then used to cluster individuals into genetic groups using the Bayesian approach (Fig. 5, Additional file 3: Figures S5-S6). This multistage process included the following steps:

- First, we phased the data with SHAPEIT v.2 [83], using the HapMap phase II b37 genetic map;
- We performed population assignments (Additional file 1: Table S1) to genetic groups (Additional file 3: Figure S5) using fineSTRUCTURE v.2 [53]. We estimated initial Ne and θ parameters using 10% of the samples and 10 Expectation-Maximization steps of the algorithm. Next, we described each individual recipient chromosome as a mixture of genetic chunks from the set of all other individuals (donors) using the estimated demographic parameters;
- We used a matrix of the copying vectors generated in the previous step to cluster the individuals using the Bayesian algorithm. We performed two parallel runs and assessed convergence between them using Gelman-Rubin statistics, as implemented in the software. Convergence was reached after 35 million MCMC iterations, including the first three million iterations, which we discarded as burn-in;
- Finally, we performed the tree-building step using default settings and used the run with the highest observed posterior likelihood to cluster the samples into genetic groups. We inspected the population dendrogram manually and assigned samples to individual groups. We excluded a few outlying samples, which showed evidence for very recent genetic admixture or incorrect population identification, from further admixture inference with Globetrotter (Additional file 3: Figure S5, Additional file 1: Table S1).

After assigning individual samples into natural genetic groups, we performed two types of Globetrotter analysis (Fig. 5), following the guidelines as in Hellenthal et al. [54]. First, in the ‘full’ analysis (Additional file 11: Table S10), we allowed the recipient individual to copy from every donor population, except from its own population label (self-copying). Second, in the ‘regional’ analysis (Additional file 11: Table S10), we grouped the genetic clusters identified by fineSTRUCTURE into three geographic regions: Europe, VUR and Western Siberia. We allowed no self-copying within regional groups (Additional file 12: Table S11 and see Note therein). In both analyses, we used additional donors from outside of the populations of interest to describe genetic ancestry, but we did not perform admixture inference for them. These included ‘W-S-Europe’, ‘Cauc/N-East’, ‘N-Cauc/C-Asia’, ‘C-Asia/S-Sib’, "E-Asia/S-Sib", ‘Far East’, ‘W-C-Sib’, ‘Sib 1’ and ‘Sib 2’ (Additional file 3: Figure S5, Additional file 12: Table S11). For all, except the ‘Nganassan’ group, we grouped all donors from Western and Central Siberia to form the ‘W-C-Sib’ cluster. For the admixture analysis of Nganasan samples, we split the ‘W-C-Sib’ cluster into ‘Sib 1’, ‘Sib 2’ and ‘Nganassan’ and excluded the latter from the generation of copying vectors to deny self-copying as explained above (see Additional file 12: Table S11 for details).

#### ALDER analysis

We used a method based on the decay of admixture linkage disequilibrium (LD) implemented in the ALDER v1.03 software [84] to test and date the admixture signal in contemporary Northern Eurasian populations (Additional file 3: Figure S7). We tested all population triplets in our dataset with pre-set ALDER v1.03 parameters (‘multiple admixture tests’ mode). We report the admixed populations with their pairs of reference populations and their inferred admixture timeframe that passed all the pre-test steps had significant *p* values and highest two-reference weighted LD curve amplitude, presenting only triplets with consistent LD decay rates if possible (Additional file 13: Table S12). Exceptions include Finns, Swedish Saami, Vepsians and Khanty whose decay time constants for all reference populations disagree by more than 25% (Additional file 13: Table S12), which may stem from bottlenecks in their demographic history [84].

#### Outgroup *f*3-statistics

We performed *f*3 analysis of our modern and published ancient human genotyping data with the AdmixTools v3.0 software package [48]. The outgroup *f*3-statistic (outgroup; X, Y) is a function of shared branch length between X and Y in the absence of admixture with the outgroup [44]. We used Yorubas as an outgroup to non-African populations and computed *f*3-statistics in the form of (Yorubas; ancient group, modern group) to investigate the shared history of a set of 47 European, Siberian and East Asian populations, including the Uralic speakers and 216 ancient genomes (Additional file 1: Table S1). We first prepared our modern dataset by excluding all positions with less than 3% genotyping success rate, and A/T and C/G polymorphisms to minimize potential strand mismatch problems. We extracted genotype information of 522,274 SNPs, which passed the filtering criteria, from the ancient DNA dataset of Mathieson et al. [85]. We divided ancient samples further into groups according to their cultural background as in the source article [85] (Additional file 1: Table S1) and merged the modern and ancient datasets. We performed an outgroup *f*3 test on all pairwise combinations between ancient and modern groups (Additional file 3: Figure S8 and Additional file 14: Table S13).

To allow for chrX versus autosomes comparison, outgroup *f*3 statistics of the form *f*3(Yorubas; test population, comparison population) were computed with Uralic-speaking populations and their geographical neighbours as test populations, and all European and Siberian populations from the EBC Illumina dataset as comparison populations. The analyses (data shown for Estonians, Udmurts and Khantys in Additional file 3: Figure S13) were run both using the same autosomal SNPs as for qpGraph (see below, Illumina chip dataset filtered by missing genotypes and minor allele frequency; 511,602 SNPs) and also all chrX positions available in the filtered dataset (12,547 SNPs). Since all individuals inherit half of their autosomal material from their father but only females inherit their chrX from their father, then in this comparison, chrX data gives more information about the female and autosomal data about the male ancestors of a population. Considering that chrY hg N3-M178 has a distinctively high frequency in Uralic-speaking populations, we used a summary statistic p(M178-coA), the probability for a pair of men sampled from two different populations to share their chrY ancestry in hg N3-M178 (calculated by multiplying hg frequencies for compared population pairs, data from Additional file 6: Table S5). This data was added to plots opposing the chrX and autosomal outgroup *f*3 results of the above-mentioned test populations to see whether those results also reflect the differences observed between chrY and mtDNA affinities among the populations are reflected also in the chrX and autosomal data (Additional file 3: Figure S13). The significance of the slope and interception of the regression lines of high (> 10%) and low (< 5%) M178-co-ancestry groups under a linear model was tested with ANOVA in R, using the car package [86].

#### qpGraph

We ran the *qpGraph* software v6.5 of the AdmixTools v4.1 package [48] on a merged dataset of modern and ancient data. To merge the two datasets, we extracted the 511,602 SNPs present in the quality filtered Illumina chip data from a dataset containing ancient samples from Lazaridis et al. [87], Jones et al. [67] and Saag et al. [66] resulting in a genotyping rate of 0.4. Only samples with at least 100,000 SNPs covered were used in the analysis. We used qpGraph with default settings, with Yorubas as an outgroup, with the useallsnps = YES option, retaining 362,380 SNPs. We were able to fit the demographic model with our data (*f*2-statistics`|*Z*-score| < 3) when we modelled ancient and modern European populations through several admixture events shown in Fig. 6 (see Additional file 3: Figure S10 for details). Of the tested Uralic-speaking populations, only Hungarians did not fit into the model.

### MtDNA and Y chromosomal variation

We present new genotype data of 1578 mtDNAs and 994 chrY of Uralic-speaking individuals, which include also all those individuals genotyped for autosomal markers. MtDNA hgs were determined by genotyping the variation of the first hypervariable segment (HVS-I) and coding region markers of mtDNA (Additional file 4: Table S3). The PCR-amplified probes were examined by RFLP or direct sequencing. The classification of mtDNA hgs follows the present nomenclature of the Global Human Mitochondrial DNA Phylogenetic Tree (mtDNA tree Build 17) [88]. The samples studied for chrY variation were genotyped for 18 NRY SNP markers at minimal, analysed by PCR/AFLP, PCR/RFLP or PCR/sequencing. The hg designation follows common nomenclature [34, 89, 90]. The hg frequencies for mtDNA and chrY were calculated and presented in a context of published data of 12,157 mtDNA (Additional file 5: Table S4) and 9730 chrY (Additional file 6: Table S5). A subset of the samples from the chrY hg Q was analysed for markers M346 and L54 [91] (Additional file 17: Table S16, Additional file 3: Figure S11) and a subset of the Selkup samples from hg R1a for marker M458.

### Correlation analysis

#### Linguistic data and lexical distances

To measure linguistic distances, we first created a Uralic family tree (Additional file 3: Figure S12A) by using Uralic basic vocabulary data and cognate coding as described in Syrjänen et al. [16] and Lehtinen et al. [92] with extension to Nganasan (data collected by BEDLAN). We used only the languages matching the ethnic identity of the individuals sampled for the genetic analyses (16 languages in total). We chose North Saami language to represent the genetic sample of Swedish Saami as that population has been sampled between the speaker areas of Lule Saami and North Saami (we do not have linguistic data on Lule Saami). We used Kildin Saami language to represent the genetic sample of Kola Saami as the sample has been collected from the classic distribution range of Kildin Saami.

Our linguistic data comprises of basic vocabulary data referring to meanings (words) that are universal, maximally resistant to borrowings and temporally stable. The Uralic basic vocabulary data and cognate assessments were achieved from the available literature. The data was collected by one single person (Jyri Lehtinen) which ensured equal quality of the data throughout the languages. In total, we had 226 meanings based on the meanings listed in Swadesh 100 and 200 lists [93, 94] and the Leipzig-Jakarta list [95]. We used the whole data without extracting the known loan words, as Lehtinen et al. [92] concludes that the loan words do not mess the evolutionary signal of the Uralic tree, but add information of the horizontal transfer of lexical material. The linguistic data was coded into binary form according to the cognacy relationships, i.e. whether the words for a meaning in two languages shared a common origin (=1) or not (=0). The phylogenetic tree was made with the MrBayes software [96] by following the settings in Syrjänen et al. [16]. The produced phylogeny resembles the ones in Syrjänen et al. [16] and Lehtinen et al. [92], has a well-supported structure following the outcomes in the earlier Uralic literature and is better resolved than many of the recent trees made with traditional linguistic methods without objective computational analyses or large data behind (see Syrjänen et al. [16] for review). The in-depth presentation of the language data, analyses, the comparison between trees, networks and earlier suggestions of the Uralic tree are given in Lehtinen et al. [92] and Syrjänen et al. [16], respectively. Some early criticism of the use of Swadesh list data by geneticists concerned the use of distance-based tree-building techniques known as ‘lexicostatistics’. Over the last decade, the application of modern Bayesian phylogenetic methods to linguistic data has allowed researchers to overcome these problems [97, 98]. We calculated pairwise linguistic distances between the tips of the phylogenetic tree (i.e. branch lengths) with the ‘ape’ package [99] in R [100]. Geographical distances were calculated as great-circle-distances (haversine) between genetic sampling locations with the ‘geosphere’ package in R [101].

To assess correlation between genetic, linguistic (lexical) and geographic distances for the Uralic-speaking populations, we employed the Mantel test [56] using Pearson’s correlation coefficient. To test whether statistically significant associations between linguistic and genetic affiliations reflect the same events in population history or parallel but separate isolation by distance processes, partial correlations keeping geography constant were performed [102]. Analyses were performed using the ‘vegan’ package [103] in R. Statistical significance was assessed using 1000 random permutations. We applied both of these tests to four types of genetic matrices. We used (1) Slatkin’s linearized Ф_ST_ [104] values calculated based on both mtDNA and chrY hg frequencies and (2) conventional F_ST_ for both mtDNA and chrY [105], (3) Weir and Cockerham [76] pairwise average F_ST_ for autosomal SNPs and (4) total variation distances (TVD) [61] between group pairs of fineSTRUCTURE chunkcount matrix (Additional file 15: Table S14). The calculations of geographic distances between populations were performed by using approximate latitude and longitude data for the sample sites (Additional file 15: Table S14I). The results of the Mantel test are presented in Additional file 16: Table S15.

#### Correlation between autosomal ADMIXTURE component k9 l and chrY hg N sublineages

Pearson correlation coefficients between two variables—the frequency of the k9 ancestral component (Fig.3b) and the frequency of chrY hg N sub-hgs in European, Volga-Uralic, Siberian and Central Asian populations —were calculated in R [100] using *cor.test()*. Results are presented in Additional file 7: Table S6.

## Additional files

Additional file 1: **Table S1.** Autosomal data used in the study. (XLSX 28 kb)

Additional file 2: **Table S2.** Ethnolinguistic characteristics of studied Uralic populations. (PDF 15 kb)

Additional file 3: **Figure S1.** Principal component analysis of the studied Uralic-speaking populations in Eurasian context. **Figure S2.** F_ST_ distances of the studied Uralic-speaking populations in the global context. **Figure S3.** ADMIXTURE analysis. **Figure S4.** *D*-statistics. **Figure S5.** fineSTRUCTURE tree. **Figure S6.** fineSTRUCTURE heatmap. **Figure S7.** Admixture dates of ALDER. **Figure S8.** Outgroup *f*3 test between modern and ancient samples. **Figure S9.** Outgroup *f*3 results plotted pairwise against each other. **Figure S10.** Admixture graphs. **Figure S11.** Phylogenetic network of Y chromosome hg Q. **Figure S12.** Trees constructed from linguistic and genetic data of Uralic peoples. **Figure S13.** Comparison of autosomal and X chromosomal outgroup *f*3 statistics for Estonians, Udmurts and Khanty. (PDF 11550 kb)

Additional file 4: **Table S3.** Mitochondrial DNA data of Uralic speakers. (XLSX 541 kb)

Additional file 5: **Table S4.** Mitochondrial DNA haplogroup frequencies in Eurasia. (XLSX 71 kb)

Additional file 6: **Table S5.** Y chromosome haplogroup frequencies in Eurasia. (XLSX 22 kb)

Additional file 7: **Table S6.** Correlation analysis of k9 ancestral component and chromosome Y haplogroup N sub-haplogroups. (XLSX 16 kb)

Additional file 8: **Table S7.** Average identity-by-descent-segment share in Northeast European populations. (XLSX 50 kb)

Additional file 9: **Table S8.** Average identity-by-descent-segment share in Central and East European populations. (XLSX 44 kb)

Additional file 10: **Table S9.** Average identity-by-descent-segment share in Siberian populations. (XLSX 41 kb)

Additional file 11: **Table S10.** Globetrotter results. (XLSX 48 kb)

Additional file 12: **Table S11.** Details of Globetrotter regional analysis. (PDF 50 kb)

Additional file 13: **Table S12.** ALDER vs Globetrotter dates. (XLSX 210 kb)

Additional file 14: **Table S13.** Outgroup *f*3 statistic. (XLSX 305 kb)

Additional file 15: **Table S14.** Data matrices for correlation analysis and geographical coordinates of studied populations. (XLSX 38 kb)

Additional file 16: **Table S15.** Mantel test results. (XLSX 11 kb)

Additional file 17: **Table S16.** Y chromosomal hg Q STR data. (XLSX 16 kb)
