# Supplementary material for: Genes reveal traces of common recent demographic history for most of the Uralic-speaking populations
Source: Genome Biol. 2018 Sep 21;19:139. doi: 10.1186/s13059-018-1522-1 (PMC6151024; doi:10.1186/s13059-018-1522-1)
Supplement: Supplementary file 2 — Table S2. Ethnolinguistic characteristics of studied Uralic populations. (PDF 15 kb) [file 13059_2018_1522_MOESM2_ESM.pdf]

Table S2. Ethnolinguistic characteristics of studied Uralic-speaking populations

Population	Country	No. of speakers*	Language subgroup**
Saami	Finland/Sweden/Norway/Russia	23 300[1]	Saami
Finn***	Finland	4 869 362[2]	Finnic
Estonian	Estonia	886 859[3]	Finnic
Karelian	Russia	60 815[5]	Finnic
Vepsian	Russia	5 936[5]	Finnic
Mari	Russia	547 605[5]	Mari
Mordovian	Russia	744 237[5]	Mordvin
Udmurt	Russia	552 299[5]	Permian
Komi	Russia	322 691[5]	Permian
Khanty	Russia	30 943[5]	Ob-Ugric
Mansi	Russia	12 269[5]	Ob-Ugric
Hungarian	Hungary	9 896 333[6]	Ugric
Nenets	Russia	44 640[5]	Samoyed
Nganasan	Russia	862[5]	Samoyed
Selkup	Russia	3 649[5]	Samoyed

* no. of speakers of given language can be much smaller than the no. of individuals with self-reported ethnicity in a population and is given only for shown country

** according to Korhonen, M. (1981). Johdatus lapin kielen historiaan (Helsinki: Suomalaisen Kirjallisuuden Seura)

*** includes also Ingrian Finns

[1] Lewis, M.P., Simons, G.F., and Fennig, C.D. (2016). Ethnologue: Languages of the World (Dallas: SIL International).

[2] Finnish census 2011 http://www.stat.fi/til/vaerak/index_en.html

[3] Estonian census 2011 http://www.stat.ee/phc2011

[4] according to the All-Russian population census 2002, from http://www.suri.ee/uralic.html

[5] according to the All-Russian population census 2010 http://www.gks.ru/free_doc/new_site/perepis2010/croc/Documents/Vol4/pub-04-01.pdf

[6] according to Hungarian census 2011 http://www.ksh.hu/nepszamlalas/tables_regional_00?lang=en
