# Supplementary material for: Genes reveal traces of common recent demographic history for most of the Uralic-speaking populations
Source: Genome Biol. 2018 Sep 21;19:139. doi: 10.1186/s13059-018-1522-1 (PMC6151024; doi:10.1186/s13059-018-1522-1)
Supplement: Supplementary file 3 — Figure S1. Principal component analysis of the studied Uralic-speaking populations in Eurasian context. Figure S2. FST distances of the studied Uralic-speaking populations in the global context. Figure S3. ADMIXTURE analysis. Figure S4. D-statistics. Figure S5. fineSTRUCTURE tree. Figure S6. fineSTRUCTURE heatmap. Figure S7. Admixture dates of ALDER. Figure S8. Outgroup f3 test between modern and ancient samples. Figure S9. Outgroup f3 results plotted pairwise against each other. Figure S10. Admixture graphs. Figure S11. Phylogenetic network of Y chromosome hg Q. Figure S12. Trees constructed from linguistic and genetic data of Uralic peoples. Figure S13. Comparison of autosomal and X chromosomal outgroup f3 statistics for Estonians, Udmurts and Khanty. (PDF 11550 kb) [file 13059_2018_1522_MOESM3_ESM.pdf]

A.

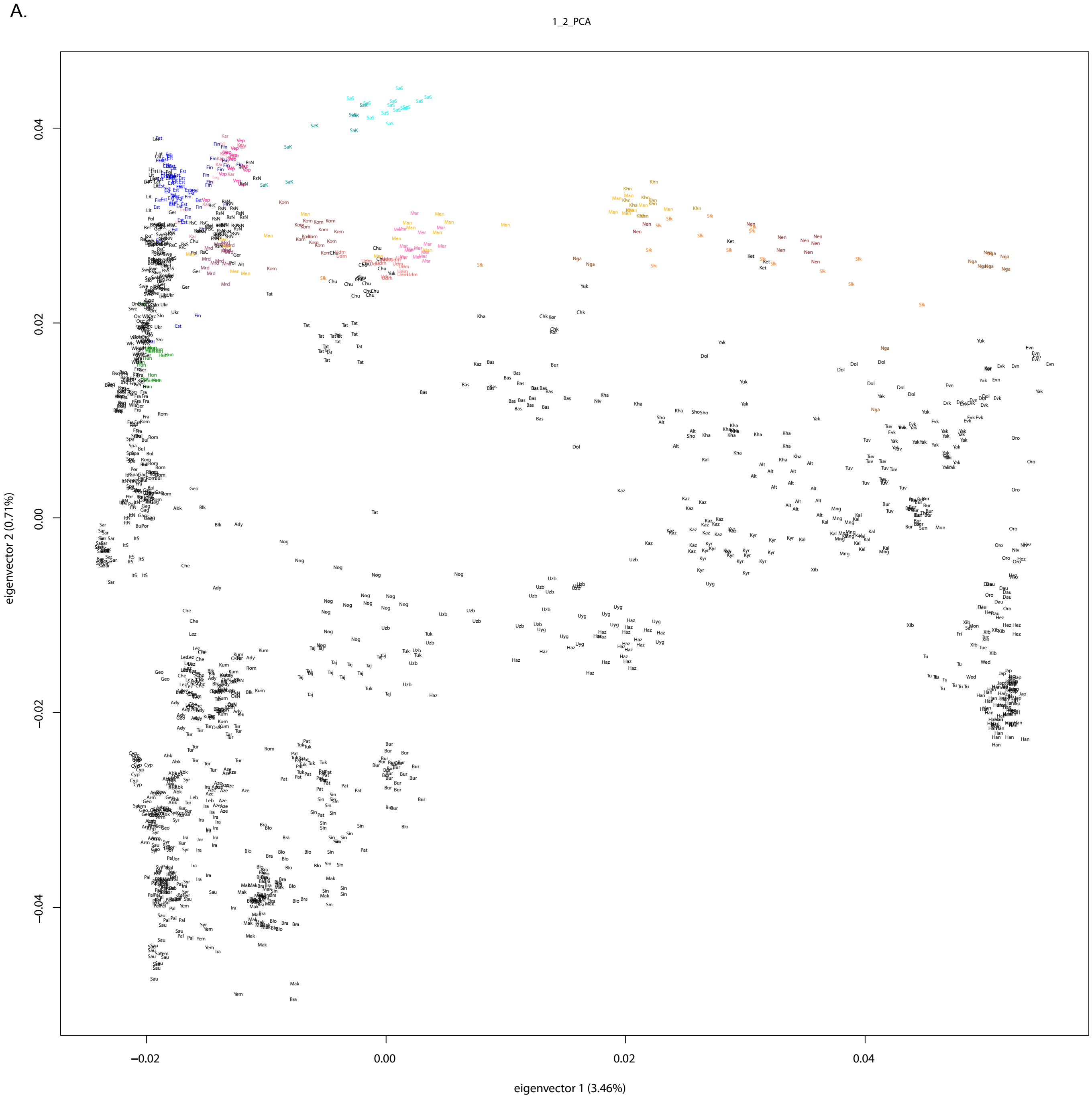

1_2_PCA
eigenvector 2 (0.71%)
eigenvector 1 (3.46%)
0.04
0.02
0.00
−0.02
−0.04
−0.02
0.00
0.02
0.04

B.

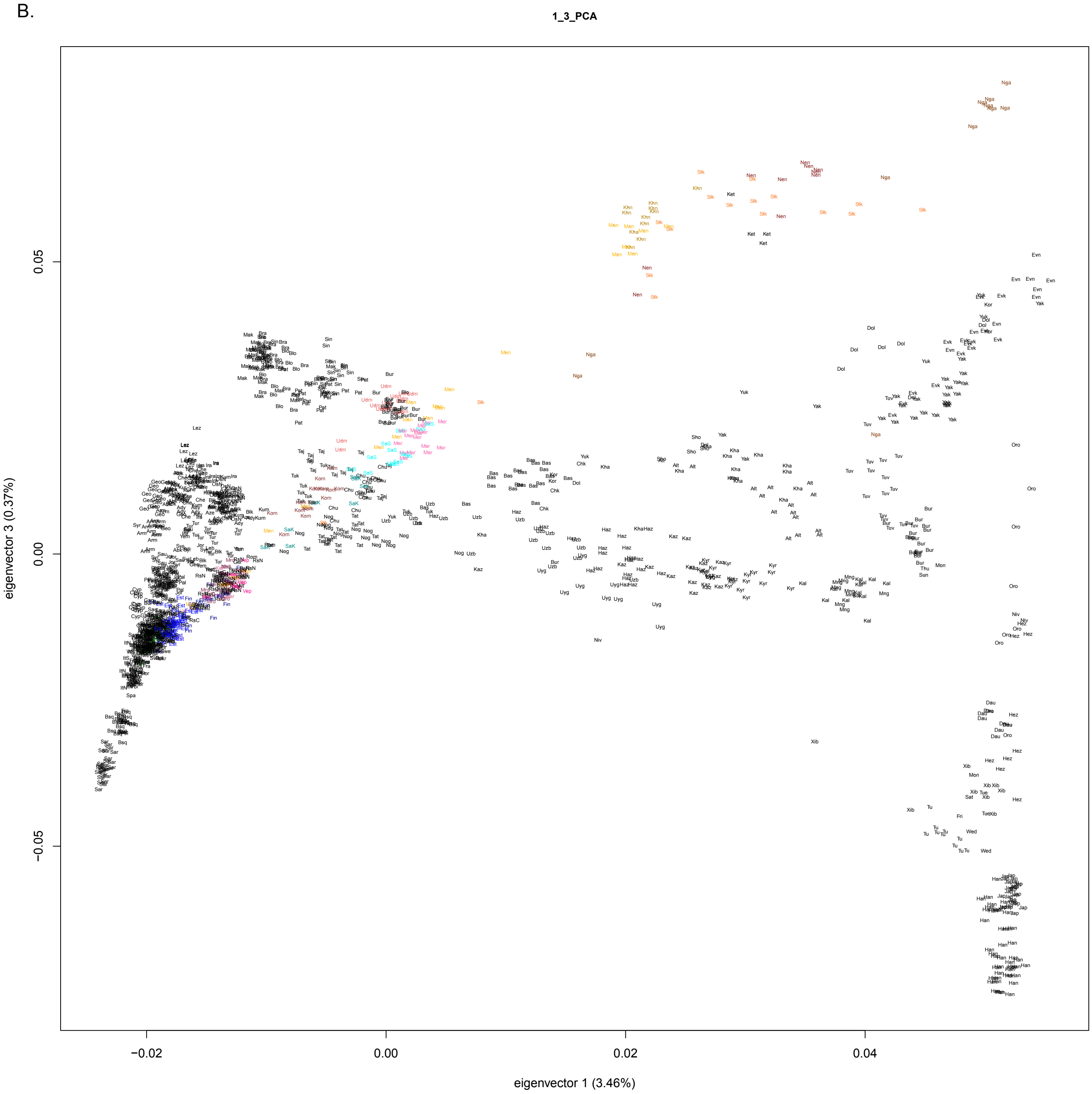
1_3_PCA
eigenvector 3 (0.37%)
eigenvector 1 (3.46%)
0.05
0.00
−0.05
−0.02
0.00
0.02
0.04

C.

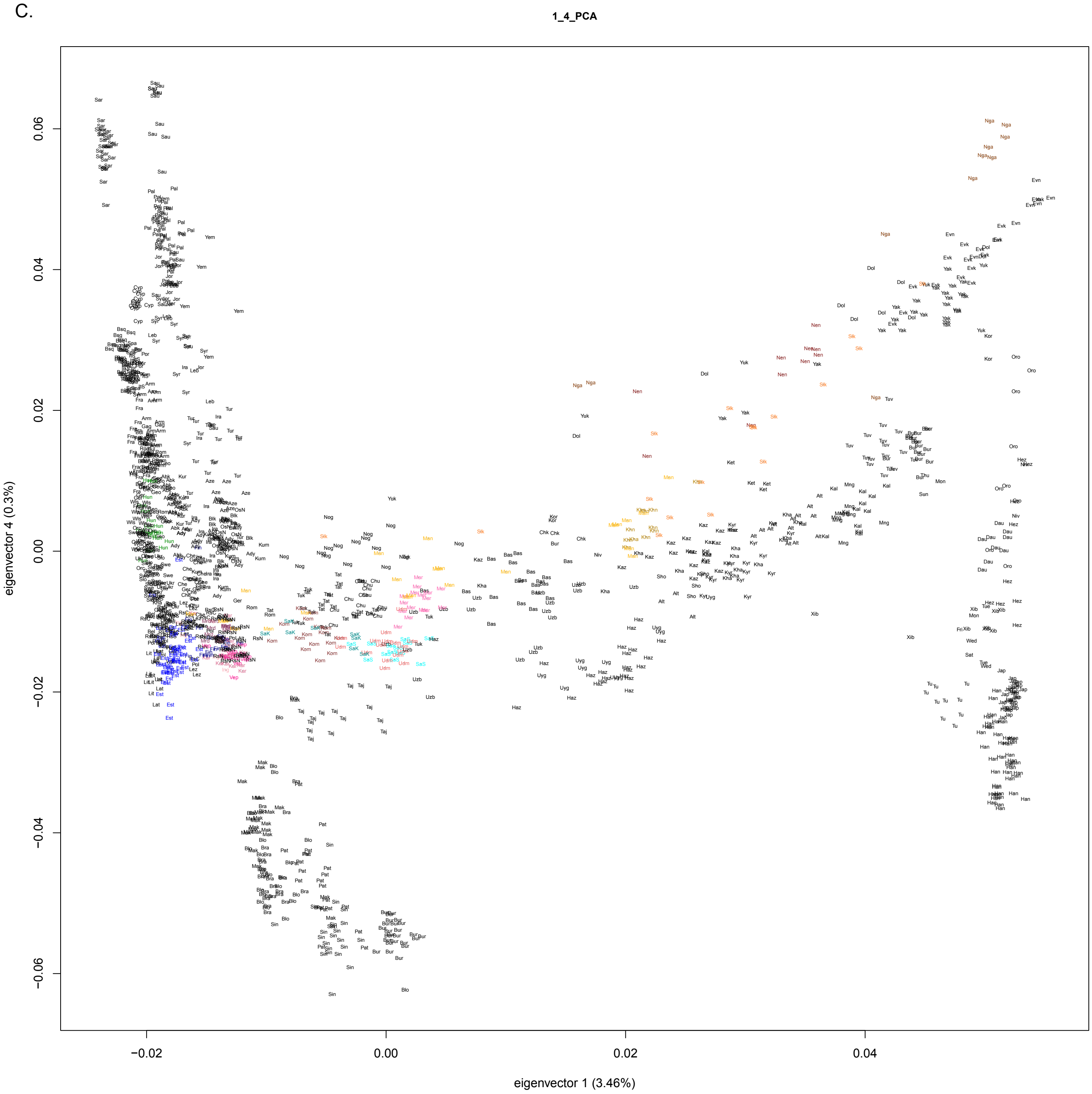
1_4_PCA
eigenvector 4 (0.3%)
eigenvector 1 (3.46%)
−0.02
0.00
0.02
0.04
0.06
−0.04
−0.06

D.

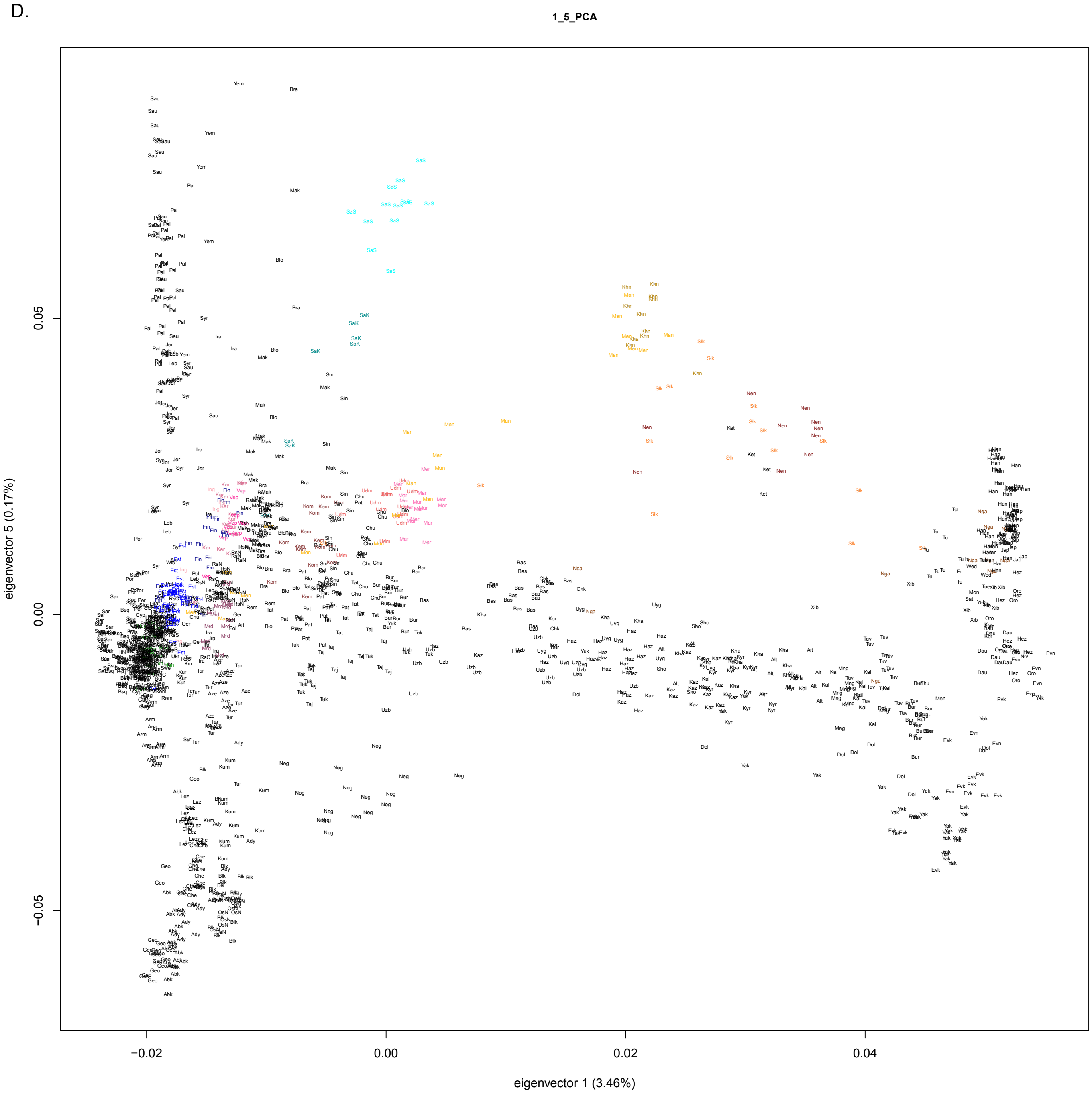
1_5_PCA
eigenvector 5 (0.17%)
0.05
0.00
−0.05
−0.02
0.00
0.02
0.04
eigenvector 1 (3.46%)

E.

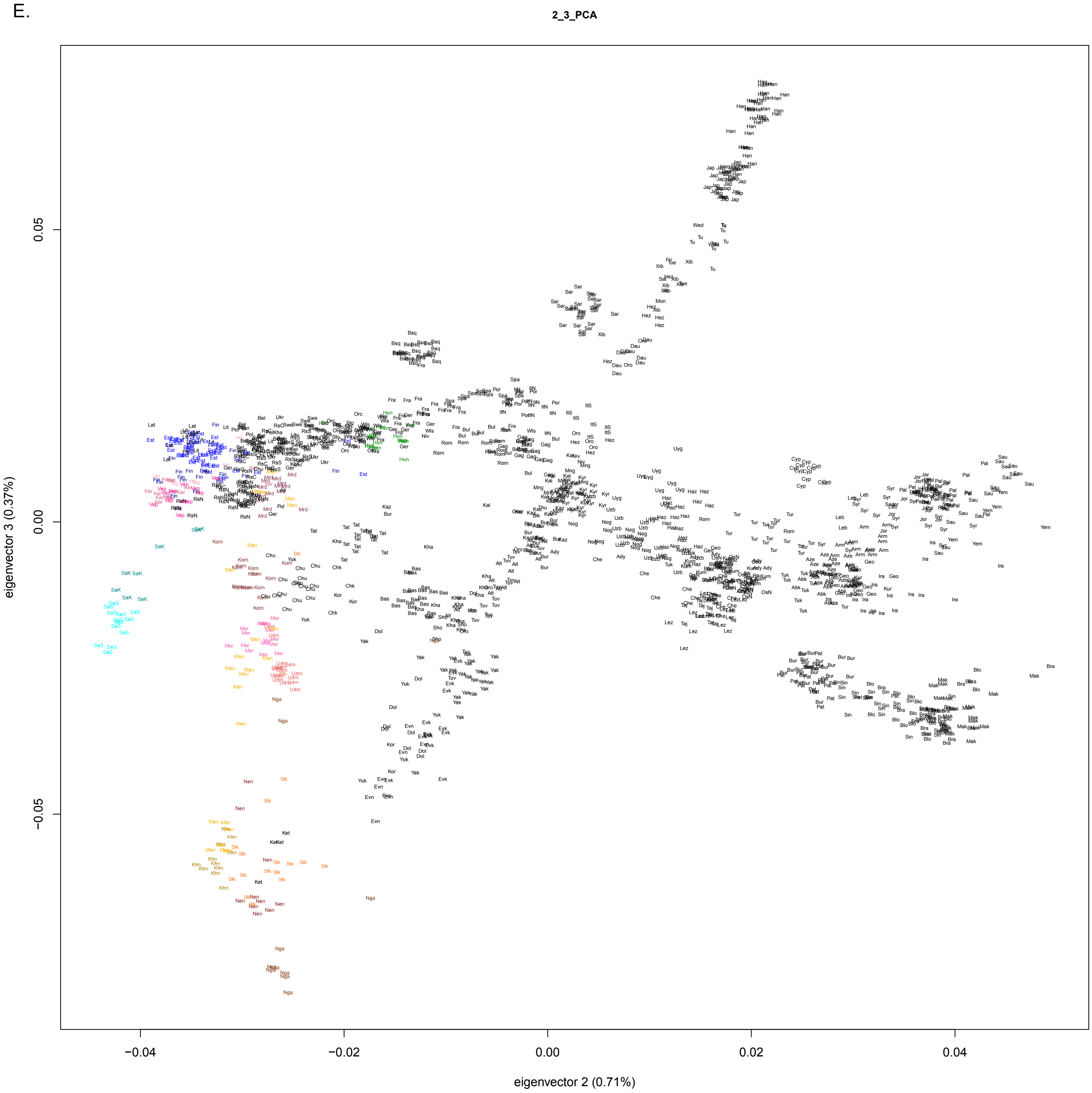
2_3_PCA
eigenvector 3 (0.37%)
eigenvector 2 (0.71%)
0.05
0.00
−0.05
−0.04
−0.02
0.00
0.02
0.04

F.

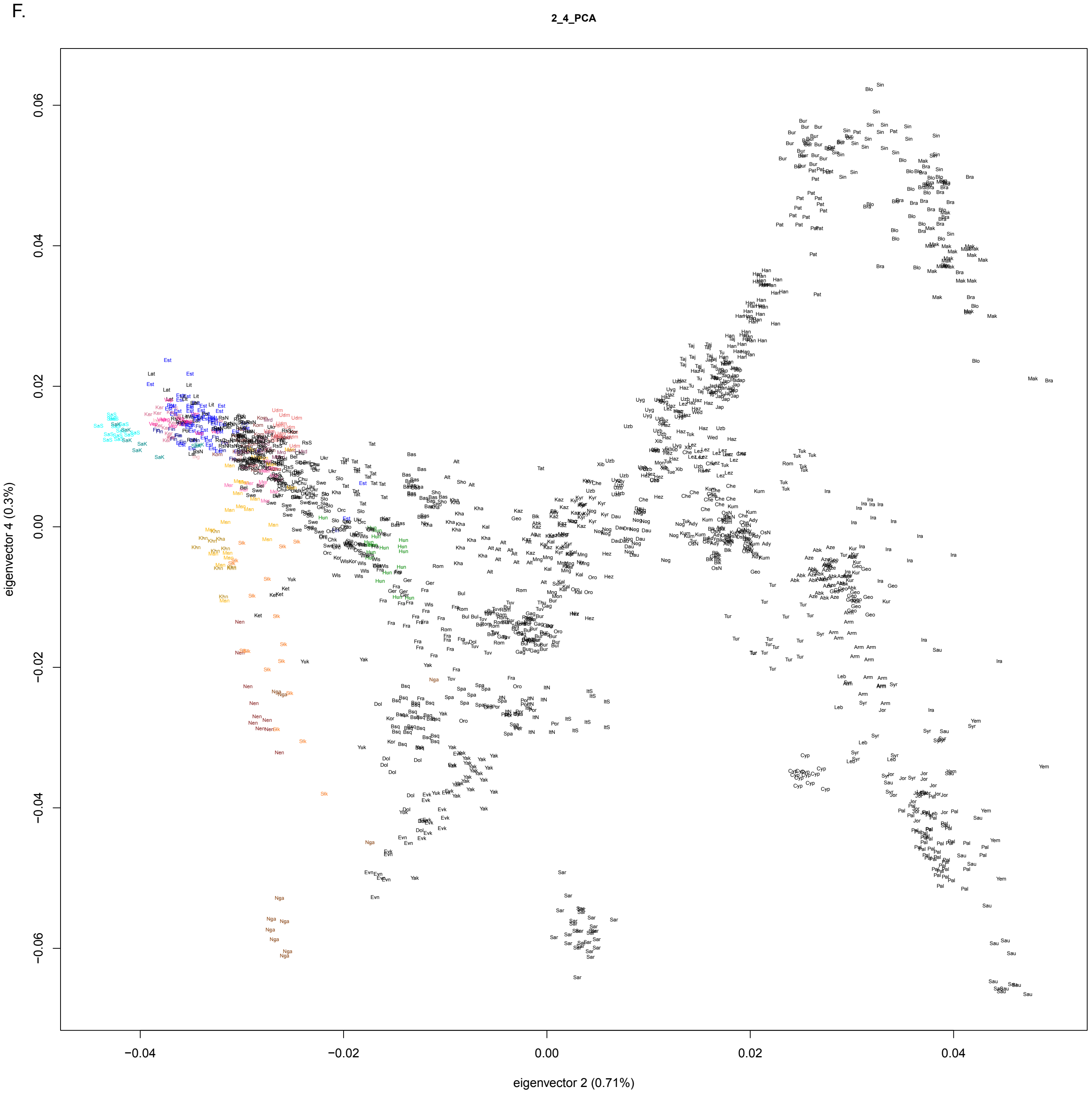
2_4_PCA
eigenvector 4 (0.3%)
eigenvector 2 (0.71%)
0.06
0.04
0.02
0.00
−0.02
−0.04
−0.06
−0.04
−0.02
0.00
0.02
0.04

G.

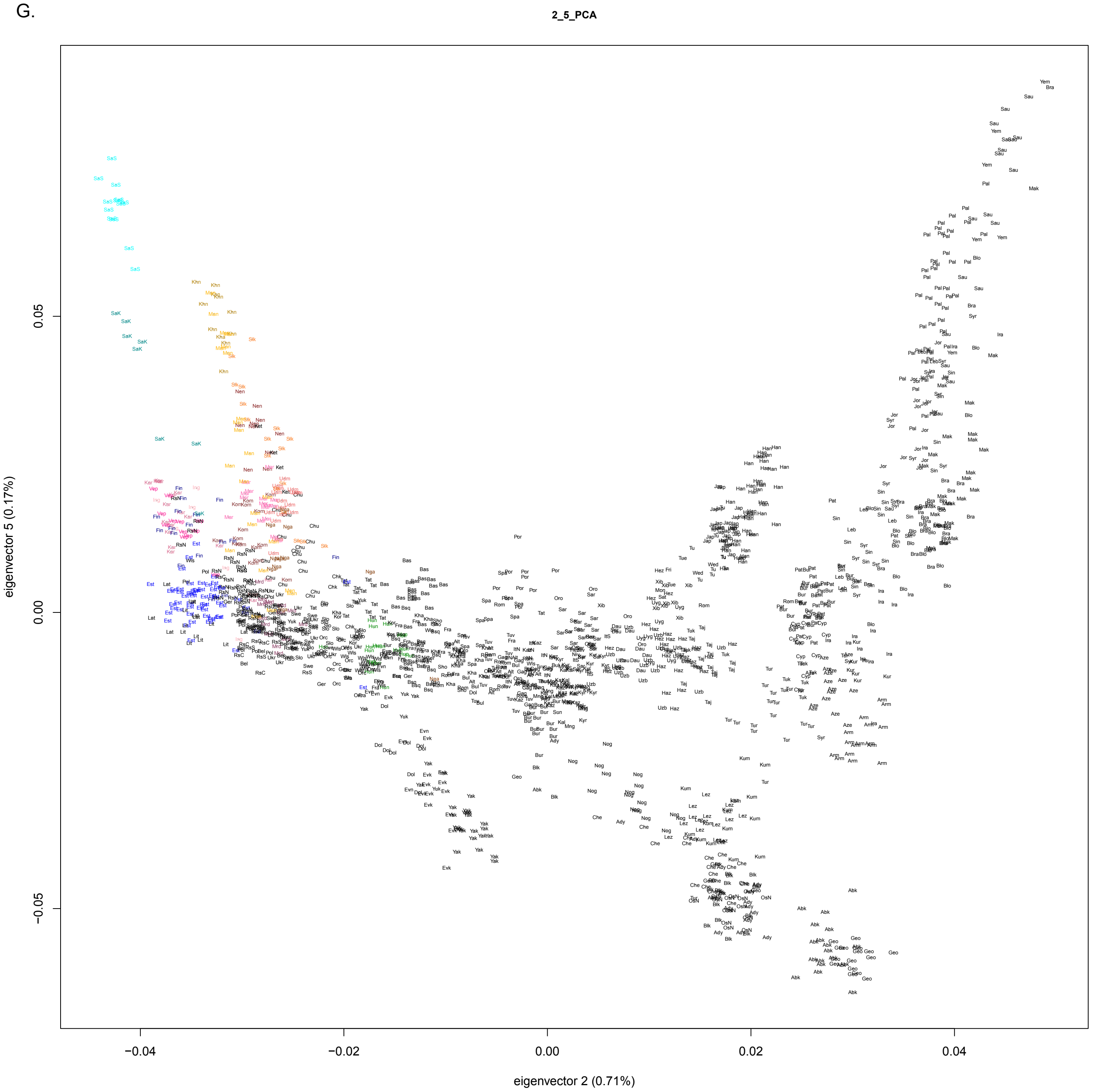
2_5_PCA
eigenvector 5 (0.17%)
0.05
0.00
-0.05
-0.04
-0.02
0.00
0.02
0.04
eigenvector 2 (0.71%)

H.

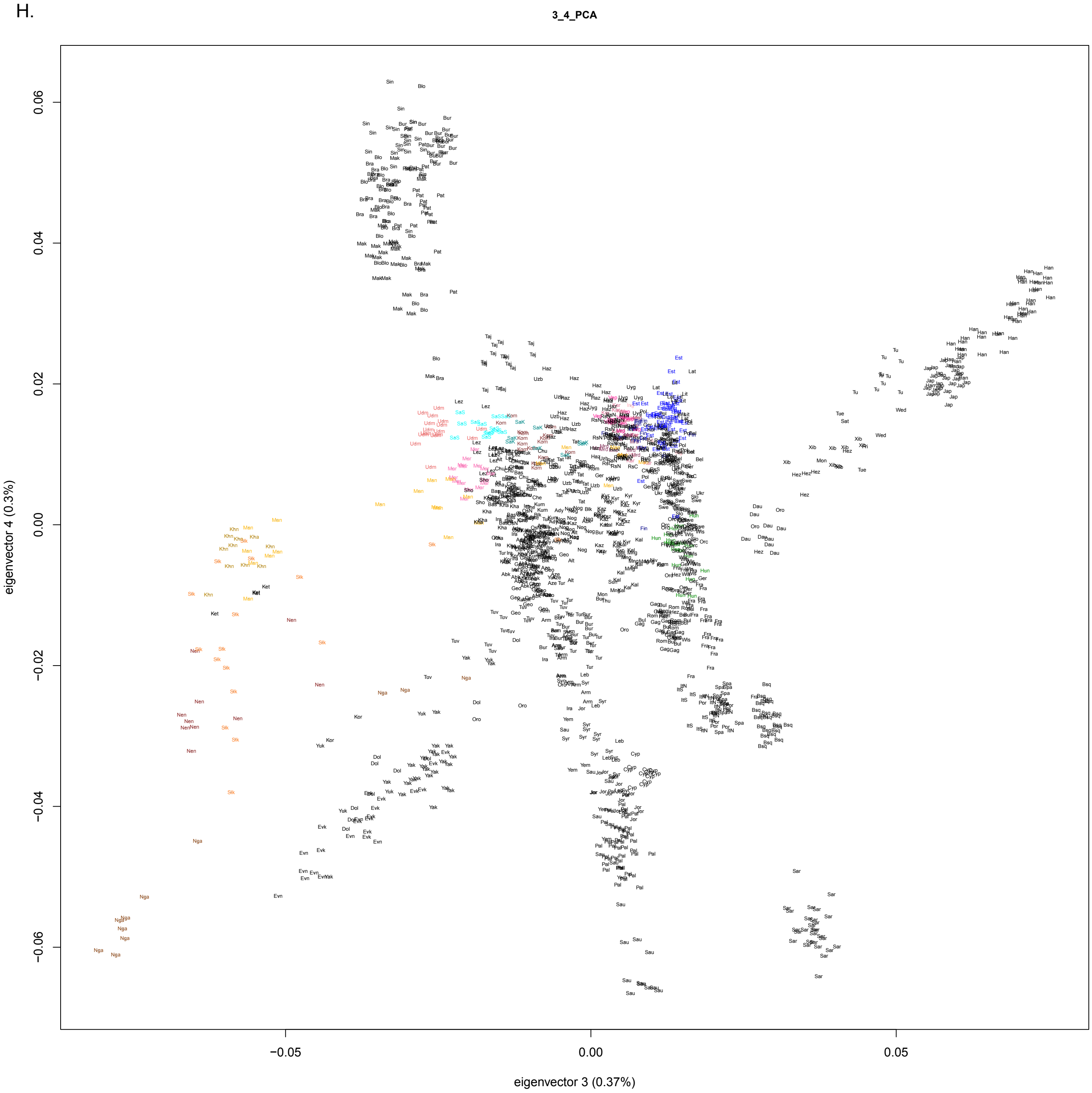
3_4_PCA
eigenvector 4 (0.3%)
eigenvector 3 (0.37%)
0.06
0.04
0.02
0.00
−0.02
−0.04
−0.06
−0.05
0.00
0.05

I.

4_5_PCA

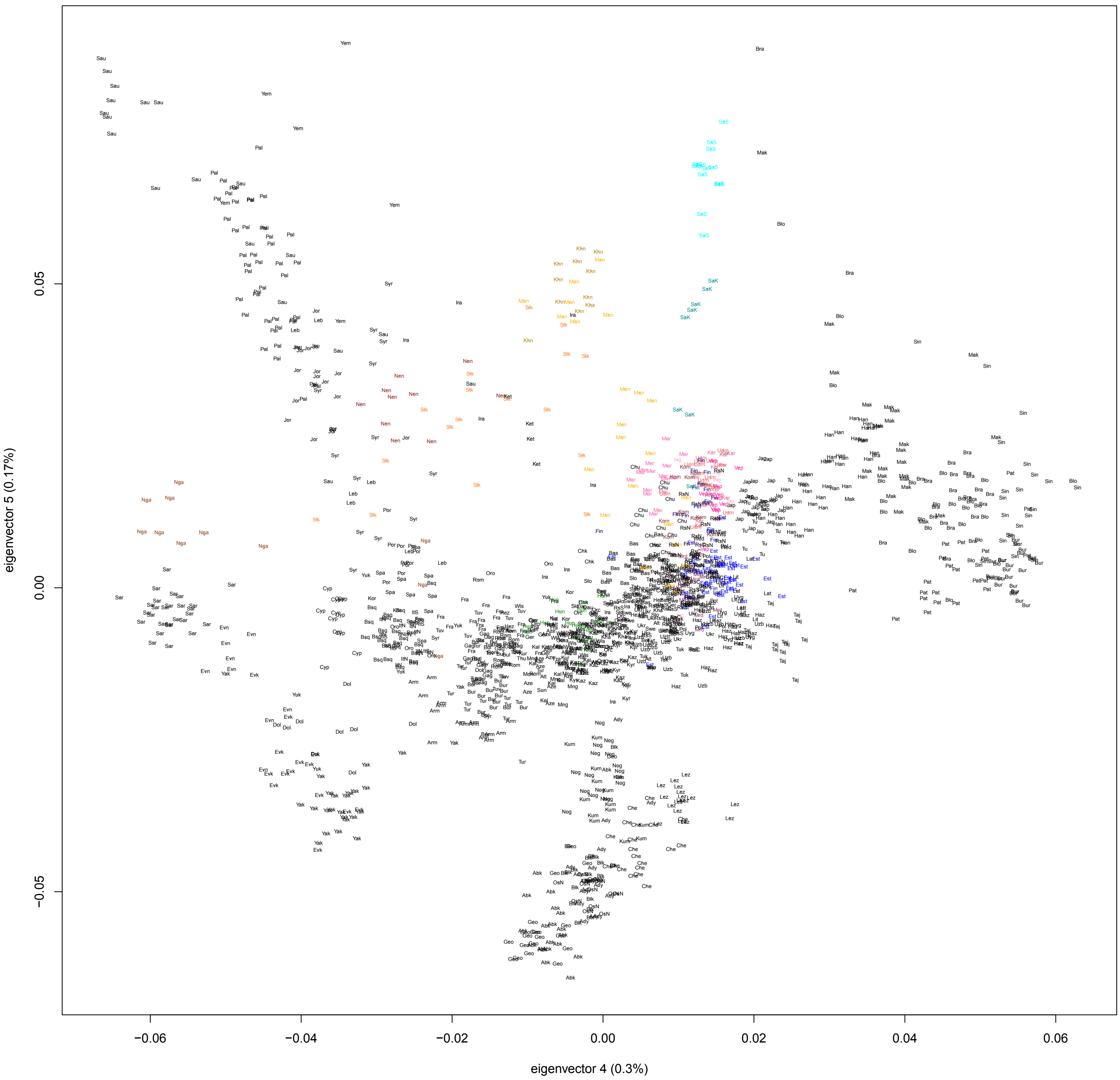

eigenvector 5 (0.17%)
0.05
0.00
−0.05
−0.06
−0.04
−0.02
0.00
0.02
0.04
0.06
eigenvector 4 (0.3%)

J.

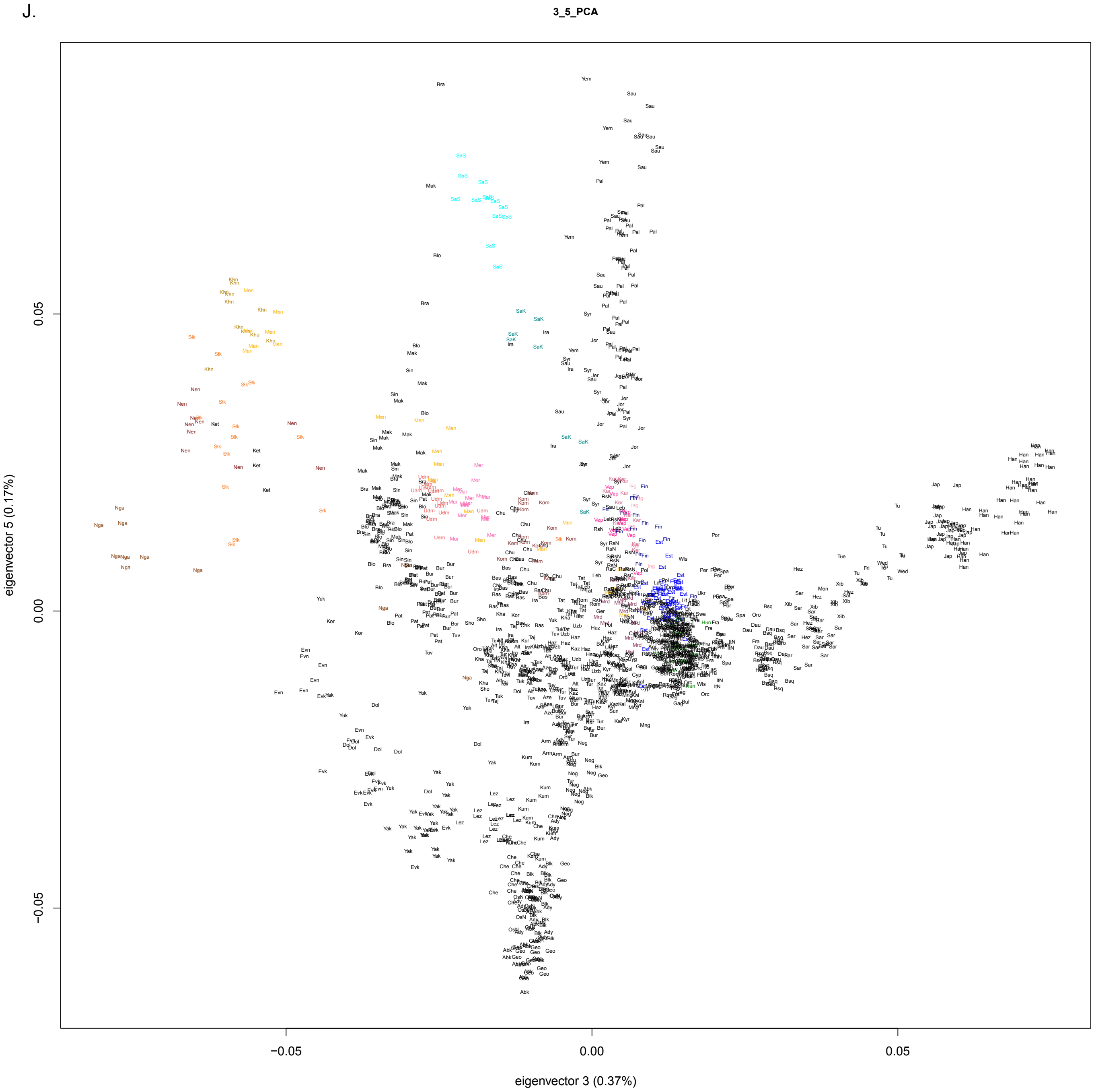

Figure S1. Principal component (PC) analysis of the studied Uralic-speaking populations (highlighted) in Eurasian context (see data and abbreviations of used populations from Additional file 1: Table S1). For each eigenvector the fraction of the variance (%) is specified; **A** – PC1 versus PC2; **B** – PC1 versus PC3; **C** – PC1 versus PC4; **D** – PC1 versus PC5; **E** – PC2 versus PC3; **F** – PC2 versus PC4; **G** – PC2 versus PC5; **H** – PC3 versus PC4; **I** – PC4 versus PC5; **J** – PC3 versus PC5.

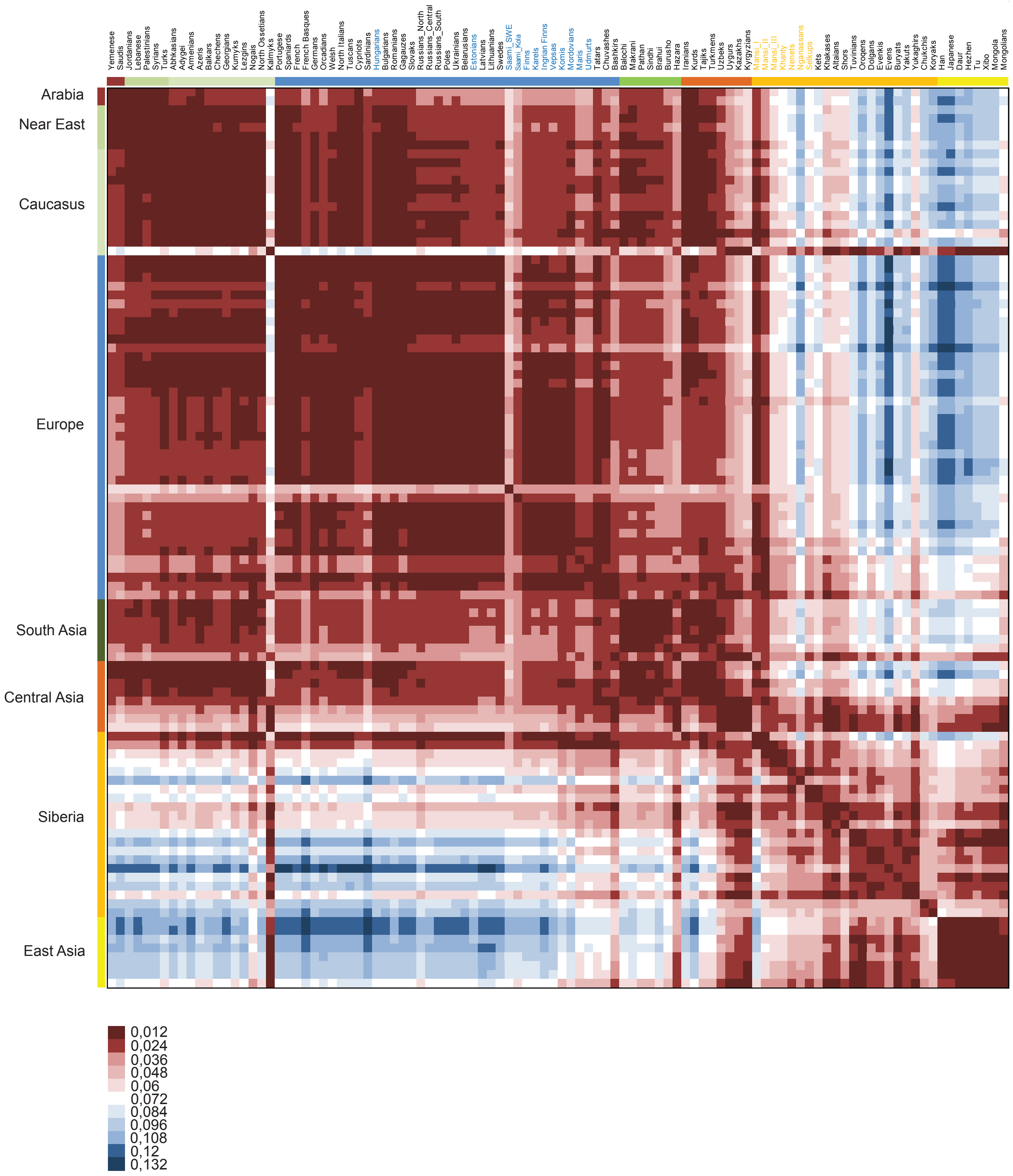

Figure S2. F_{st}-distances of the studied Uralic-speaking populations in the global context. Regional groups are indicated with color bars along the axes of the distance matrix. Uralic-speaking populations are with blue (European) or orange (Siberian) font. Color codes for the values of the genetic distances between population pairs are shown with the brown-to-blue (lowest to highest) bar below the matrix.

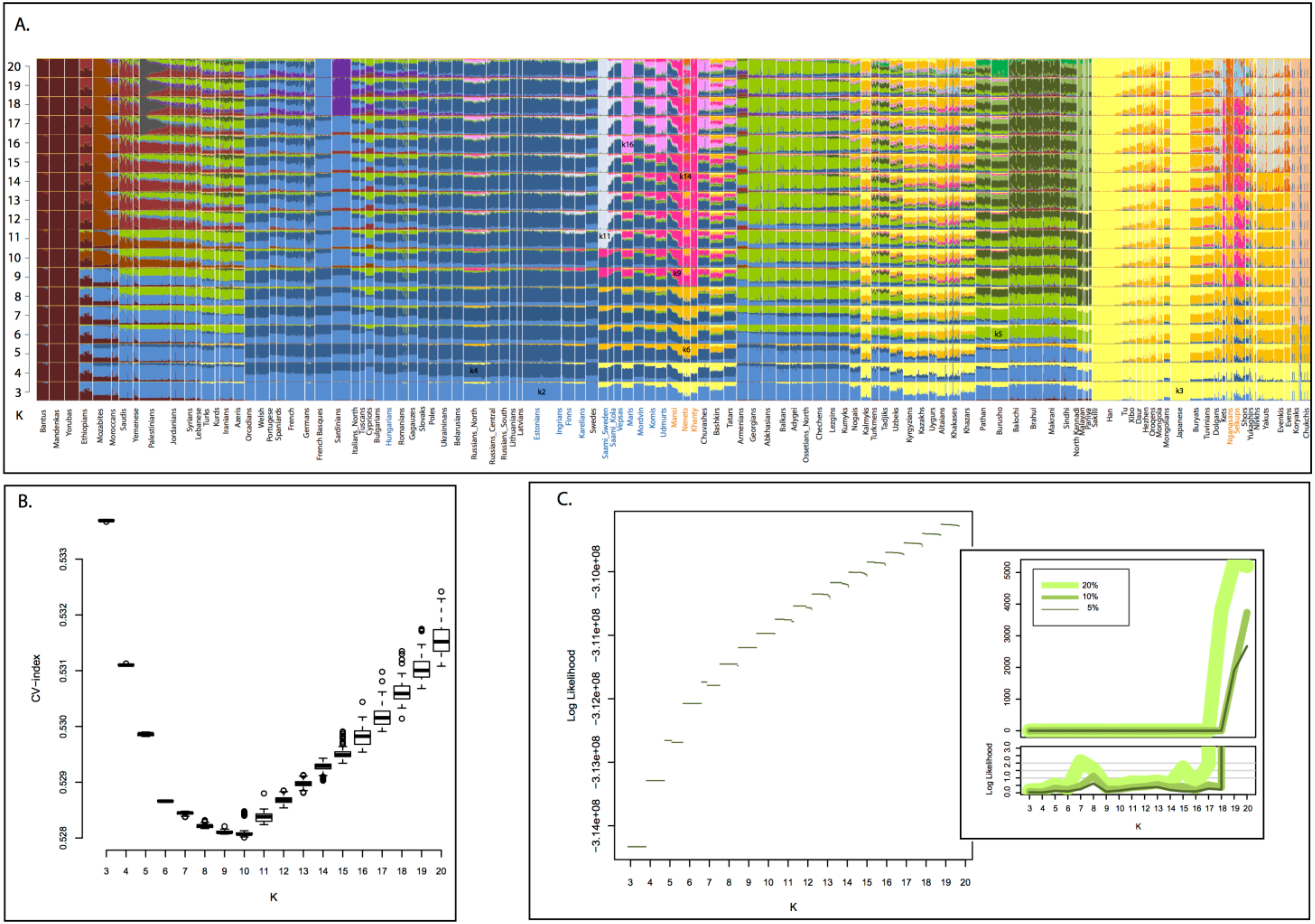

A.
K
20
19
18
17
16
15
14
13
12
11
10
9
8
7
6
5
4
3
k16
k14
k11
k9
k6
k5
k4
k3
k2
Bantus
Mandenkas
Yorubas
Ethiopians
Mozabites
Moroccans
Saudis
Yemenese
Palestinians
Jordanians
Syrians
Lebanese
Turks
Kurds
Iranians
Azeris
Orcadians
Welsh
Portugese
Spaniards
French
Germans
French Basques
Sardinians
Italians_North
Tuscans
Cypriots
Bulgarians
Hungarians
Romanians
Gagauzes
Slovaks
Poles
Ukrainians
Belarusians
Russians_North
Russians_Central
Russians_South
Lithuanians
Latvians
Estonians
Ingrians
Finns
Karelians
Swedes
Saami_Sweden
Saami_Kola
Vepsas
Maris
Mordvin
Komis
Udmurts
Mansi
Nenets
Khanty
Chuvashes
Bashkirs
Tatars
Armenians
Georgians
Abkhasians
Balkars
Adygei
Ossetians_North
Chechens
Lezgins
Kumyks
Nogais
Kalmyks
Turkmens
Tadjiks
Uzbeks
Kyrgyzians
Kazakhs
Uygurs
Altaians
Khakases
Khazars
Pathan
Burusho
Balochi
Brahui
Makrani
Sindhi
North Kannadi
Malayan
Pariyan
Sakilli
Han
Tu
Xibo
Daur
Hezhen
Oroqens
Mongola
Mongolians
Japanese
Buryats
Tuvinians
Dolgans
Kets
Nganasans
Selkups
Shors
Yukaghirs
Nivkhs
Yakuts
Evenkis
Evens
Koryaks
Chukchis
B.
CV-index
0.528
0.529
0.530
0.531
0.532
0.533
K
3 4 5 6 7 8 9 10 11 12 13 14 15 16 17 18 19 20
C.
Log Likelihood
-3.14e+08
-3.13e+08
-3.12e+08
-3.11e+08
-3.10e+08
K
3 4 5 6 7 8 9 10 11 12 13 14 15 16 17 18 19 20
20%
10%
5%
0
1000
2000
3000
4000
5000
Log Likelihood
0.0 1.0 2.0 3.0
3 4 5 6 7 8 9 10 11 12 13 14 15 16 17 18 19 20
K

Figure S3. ADMIXTURE plots of the Uralic-speaking populations in a global context based on autosomal SNPs and with the number of assumed ancestral populations (K) ranging from 3 to 20. **A.** Bar plot displaying individual ancestry estimates for studied populations. **B.** Box and whiskers plot of the cross validation (CV) indexes of all K x 100 runs of ADMIXTURE; **C.** Log-likelihood (LL) scores of all K x 100 runs of ADMIXTURE. Inset shows the variation in the fractions (5%, 10% and 20%) of runs that reached the highest LL values.

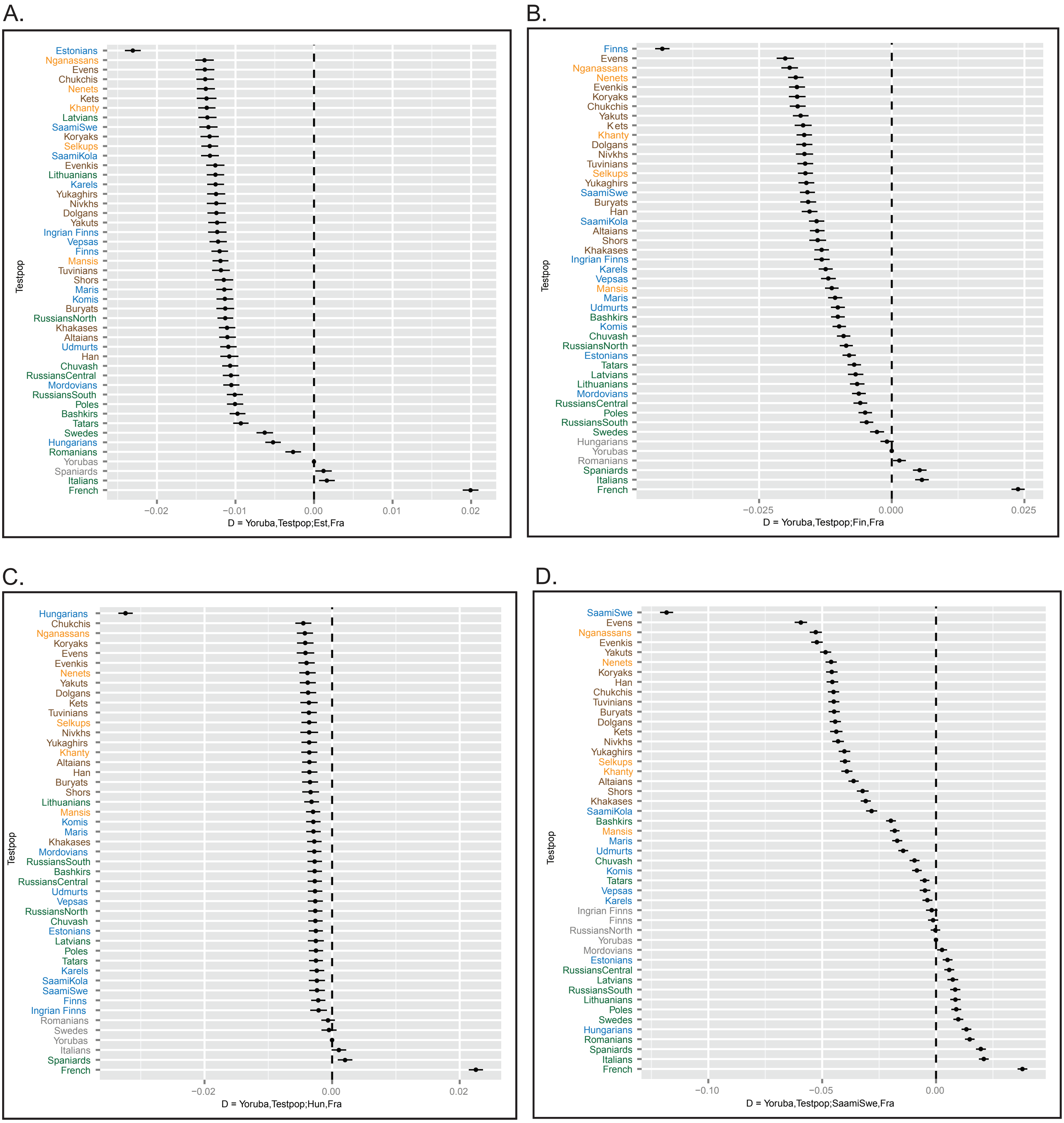
A.
Estonians
Nganassans
Evens
Chukchis
Nenets
Kets
Khanty
Latvians
SaamiSwe
Koryaks
Selkups
SaamiKola
Evenkis
Lithuanians
Karels
Yukaghirs
Nivkhs
Dolgans
Yakuts
Ingrian Finns
Vepsas
Finns
Mansis
Tuvinians
Shors
Maris
Komis
Buryats
RussiansNorth
Khakases
Altaians
Udmurts
Han
Chuvash
RussiansCentral
Mordovians
RussiansSouth
Poles
Bashkirs
Tatars
Swedes
Hungarians
Romanians
Yorubas
Spaniards
Italians
French
Testpop
−0.02
−0.01
0.00
0.01
0.02
D = Yoruba,Testpop;Est,Fra
B.
Finns
Evens
Nganassans
Nenets
Evenkis
Koryaks
Chukchis
Yakuts
Kets
Khanty
Dolgans
Nivkhs
Tuvinians
Selkups
Yukaghirs
SaamiSwe
Buryats
Han
SaamiKola
Altaians
Shors
Khakases
Ingrian Finns
Karels
Vepsas
Mansis
Maris
Udmurts
Bashkirs
Komis
Chuvash
RussiansNorth
Estonians
Tatars
Latvians
Lithuanians
Mordovians
RussiansCentral
Poles
RussiansSouth
Swedes
Hungarians
Yorubas
Romanians
Spaniards
Italians
French
Testpop
−0.025
0.000
0.025
D = Yoruba,Testpop;Fin,Fra
C.
Hungarians
Chukchis
Nganassans
Koryaks
Evens
Evenkis
Nenets
Yakuts
Dolgans
Kets
Tuvinians
Selkups
Nivkhs
Yukaghirs
Khanty
Altaians
Han
Buryats
Shors
Lithuanians
Mansis
Komis
Maris
Khakases
Mordovians
RussiansSouth
Bashkirs
RussiansCentral
Udmurts
Vepsas
RussiansNorth
Chuvash
Estonians
Latvians
Poles
Tatars
Karels
SaamiKola
SaamiSwe
Finns
Ingrian Finns
Romanians
Swedes
Yorubas
Italians
Spaniards
French
Testpop
−0.02
0.00
0.02
D = Yoruba,Testpop;Hun,Fra
D.
SaamiSwe
Evens
Nganassans
Evenkis
Yakuts
Nenets
Koryaks
Han
Chukchis
Tuvinians
Buryats
Dolgans
Kets
Nivkhs
Yukaghirs
Selkups
Khanty
Altaians
Shors
Khakases
SaamiKola
Bashkirs
Mansis
Maris
Udmurts
Chuvash
Komis
Tatars
Vepsas
Karels
Ingrian Finns
Finns
RussiansNorth
Yorubas
Mordovians
Estonians
RussiansCentral
Latvians
RussiansSouth
Lithuanians
Poles
Swedes
Hungarians
Romanians
Spaniards
Italians
French
Testpop
−0.10
−0.05
0.00
D = Yoruba,Testpop;SaamiSwe,Fra

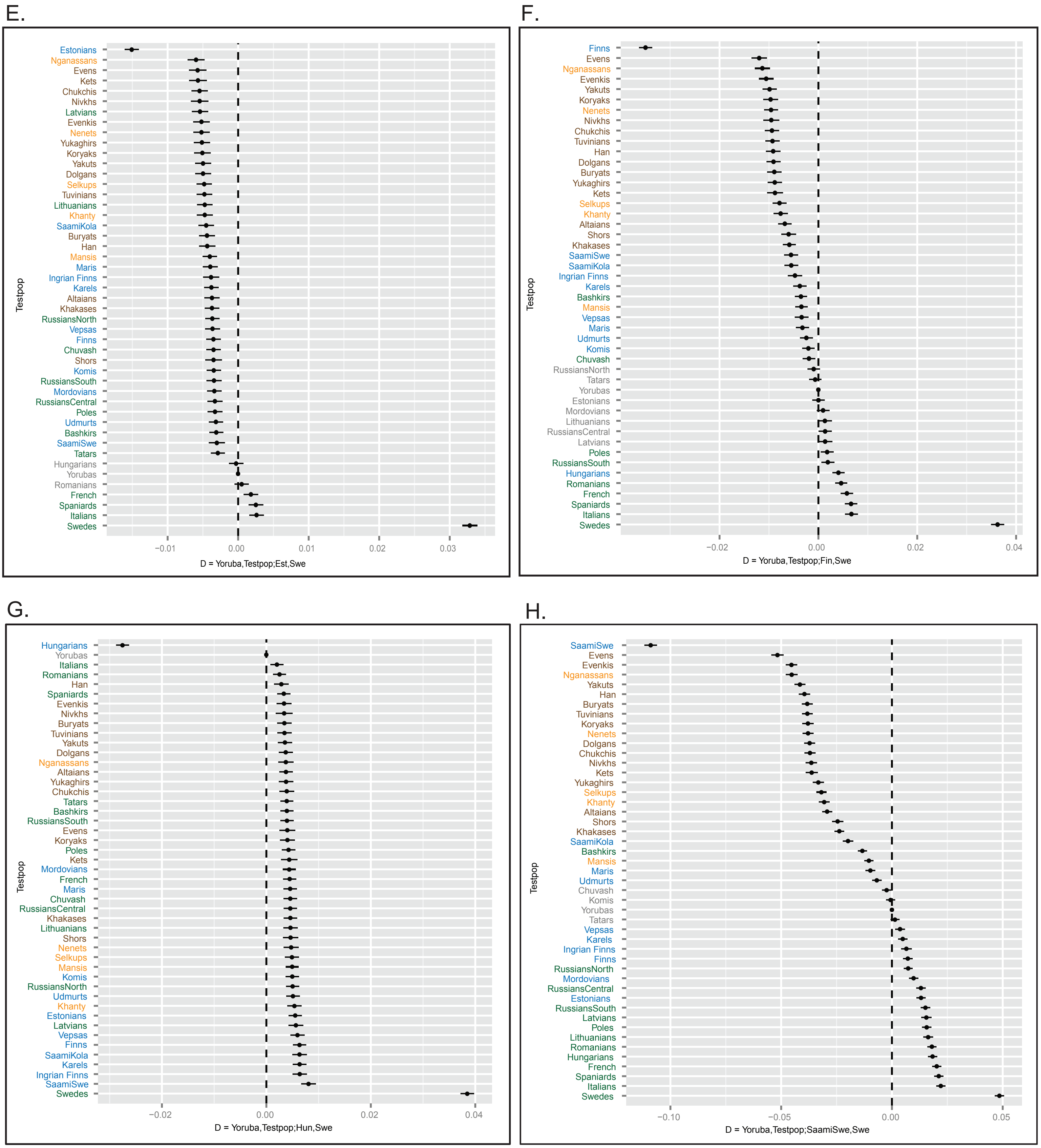

E.
Testpop
Estonians
Nganassans
Evens
Kets
Chukchis
Nivkhs
Latvians
Evenkis
Nenets
Yukaghirs
Koryaks
Yakuts
Dolgans
Selkups
Tuvinians
Lithuanians
Khanty
SaamiKola
Buryats
Han
Mansis
Maris
Ingrian Finns
Karels
Altaians
Khakases
RussiansNorth
Vepsas
Finns
Chuvash
Shors
Komis
RussiansSouth
Mordovians
RussiansCentral
Poles
Udmurts
Bashkirs
SaamiSwe
Tatars
Hungarians
Yorubas
Romanians
French
Spaniards
Italians
Swedes
−0.01
0.00
0.01
0.02
0.03
D = Yoruba,Testpop;Est,Swe
F.
Testpop
Finns
Evens
Nganassans
Evenkis
Yakuts
Koryaks
Nenets
Nivkhs
Chukchis
Tuvinians
Han
Dolgans
Buryats
Yukaghirs
Kets
Selkups
Khanty
Altaians
Shors
Khakases
SaamiSwe
SaamiKola
Ingrian Finns
Karels
Bashkirs
Mansis
Vepsas
Maris
Udmurts
Komis
Chuvash
RussiansNorth
Tatars
Yorubas
Estonians
Mordovians
Lithuanians
RussiansCentral
Latvians
Poles
RussiansSouth
Hungarians
Romanians
French
Spaniards
Italians
Swedes
−0.02
0.00
0.02
0.04
D = Yoruba,Testpop;Fin,Swe
G.
Testpop
Hungarians
Yorubas
Italians
Romanians
Han
Spaniards
Evenkis
Nivkhs
Buryats
Tuvinians
Yakuts
Dolgans
Nganassans
Altaians
Yukaghirs
Chukchis
Tatars
Bashkirs
RussiansSouth
Evens
Koryaks
Poles
Kets
Mordovians
French
Maris
Chuvash
RussiansCentral
Khakases
Lithuanians
Shors
Nenets
Selkups
Mansis
Komis
RussiansNorth
Udmurts
Khanty
Estonians
Latvians
Vepsas
Finns
SaamiKola
Karels
Ingrian Finns
SaamiSwe
Swedes
−0.02
0.00
0.02
0.04
D = Yoruba,Testpop;Hun,Swe
H.
Testpop
SaamiSwe
Evens
Evenkis
Nganassans
Yakuts
Han
Buryats
Tuvinians
Koryaks
Nenets
Dolgans
Chukchis
Nivkhs
Kets
Yukaghirs
Selkups
Khanty
Altaians
Shors
Khakases
SaamiKola
Bashkirs
Mansis
Maris
Udmurts
Chuvash
Komis
Yorubas
Tatars
Vepsas
Karels
Ingrian Finns
Finns
RussiansNorth
Mordovians
RussiansCentral
Estonians
RussiansSouth
Latvians
Poles
Lithuanians
Romanians
Hungarians
French
Spaniards
Italians
Swedes
−0.10
−0.05
0.00
0.05
D = Yoruba,Testpop;SaamiSwe,Swe

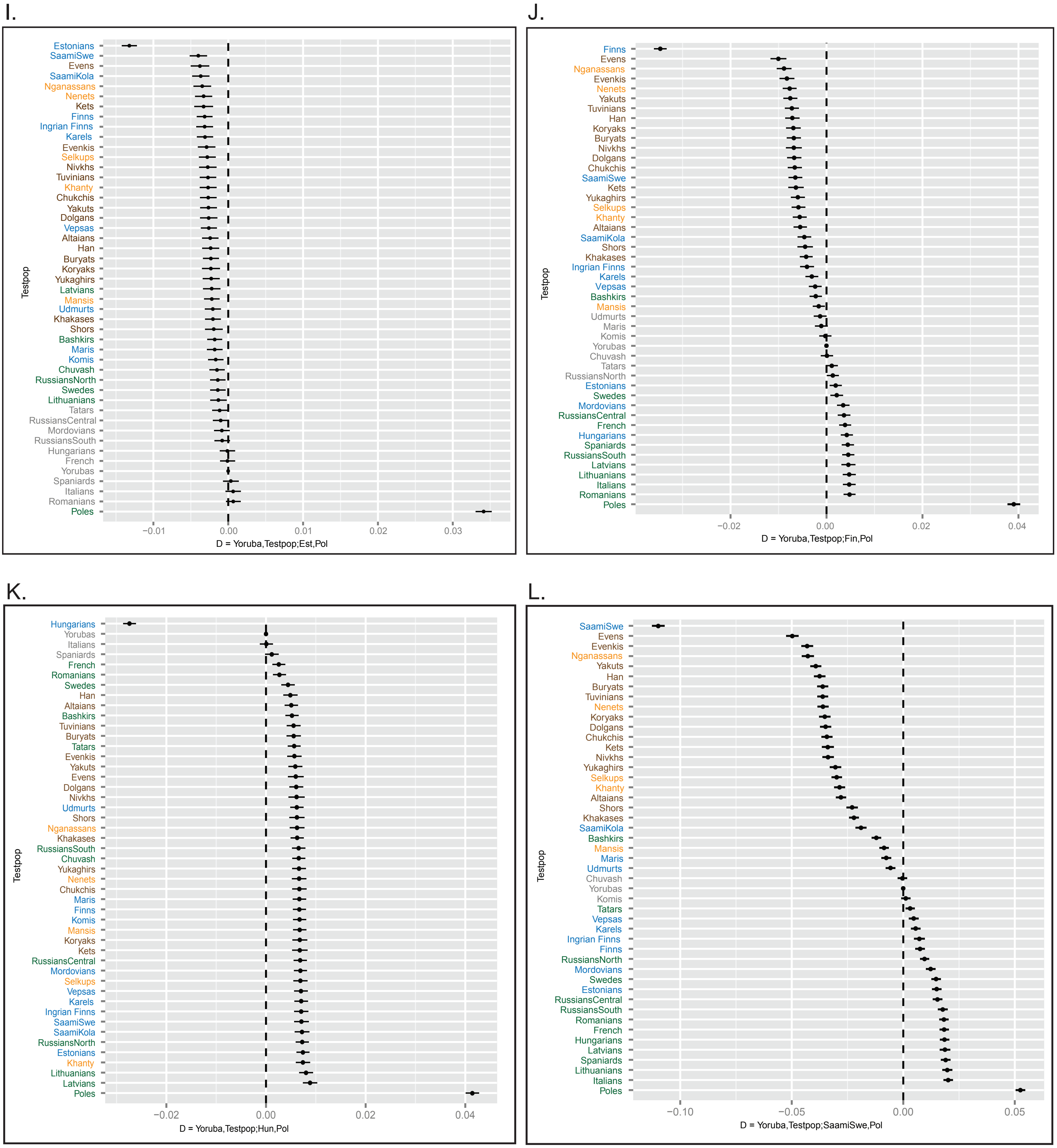
I.
Estonians
SaamiSwe
Evens
SaamiKola
Nganassans
Nenets
Kets
Finns
Ingrian Finns
Karels
Evenkis
Selkups
Nivkhs
Tuvinians
Khanty
Chukchis
Yakuts
Dolgans
Vepsas
Altaians
Han
Buryats
Koryaks
Yukaghirs
Latvians
Mansis
Udmurts
Khakases
Shors
Bashkirs
Maris
Komis
Chuvash
RussiansNorth
Swedes
Lithuanians
Tatars
RussiansCentral
Mordovians
RussiansSouth
Hungarians
French
Yorubas
Spaniards
Italians
Romanians
Poles
Testpop
−0.01
0.00
0.01
0.02
0.03
D = Yoruba,Testpop;Est,Pol
J.
Finns
Evens
Nganassans
Evenkis
Nenets
Yakuts
Tuvinians
Han
Koryaks
Buryats
Nivkhs
Dolgans
Chukchis
SaamiSwe
Kets
Yukaghirs
Selkups
Khanty
Altaians
SaamiKola
Shors
Khakases
Ingrian Finns
Karels
Vepsas
Bashkirs
Mansis
Udmurts
Maris
Komis
Yorubas
Chuvash
Tatars
RussiansNorth
Estonians
Swedes
Mordovians
RussiansCentral
French
Hungarians
Spaniards
RussiansSouth
Latvians
Lithuanians
Italians
Romanians
Poles
Testpop
−0.02
0.00
0.02
0.04
D = Yoruba,Testpop;Fin,Pol
K.
Hungarians
Yorubas
Italians
Spaniards
French
Romanians
Swedes
Han
Altaians
Bashkirs
Tuvinians
Buryats
Tatars
Evenkis
Yakuts
Evens
Dolgans
Nivkhs
Udmurts
Shors
Nganassans
Khakases
RussiansSouth
Chuvash
Yukaghirs
Nenets
Chukchis
Maris
Finns
Komis
Mansis
Koryaks
Kets
RussiansCentral
Mordovians
Selkups
Vepsas
Karels
Ingrian Finns
SaamiSwe
SaamiKola
RussiansNorth
Estonians
Khanty
Lithuanians
Latvians
Poles
Testpop
−0.02
0.00
0.02
0.04
D = Yoruba,Testpop;Hun,Pol
L.
SaamiSwe
Evens
Evenkis
Nganassans
Yakuts
Han
Buryats
Tuvinians
Nenets
Koryaks
Dolgans
Chukchis
Kets
Nivkhs
Yukaghirs
Selkups
Khanty
Altaians
Shors
Khakases
SaamiKola
Bashkirs
Mansis
Maris
Udmurts
Chuvash
Yorubas
Komis
Tatars
Vepsas
Karels
Ingrian Finns
Finns
RussiansNorth
Mordovians
Swedes
Estonians
RussiansCentral
RussiansSouth
Romanians
French
Hungarians
Latvians
Spaniards
Lithuanians
Italians
Poles
Testpop
−0.10
−0.05
0.00
0.05
D = Yoruba,Testpop;SaamiSwe,Pol

M.

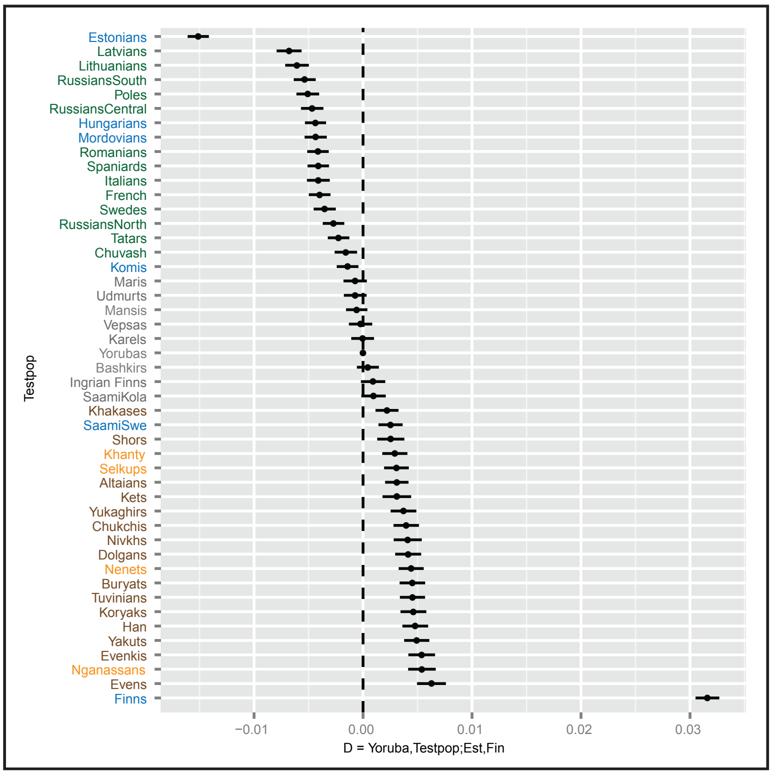
Estonians
Latvians
Lithuanians
RussiansSouth
Poles
RussiansCentral
Hungarians
Mordovians
Romanians
Spaniards
Italians
French
Swedes
RussiansNorth
Tatars
Chuvash
Komis
Maris
Udmurts
Mansis
Vepsas
Karels
Yorubas
Bashkirs
Ingrian Finns
SaamiKola
Khakases
SaamiSwe
Shors
Khanty
Selkups
Altaians
Kets
Yukaghirs
Chukchis
Nivkhs
Dolgans
Nenets
Buryats
Tuvinians
Koryaks
Han
Yakuts
Evenkis
Nganassans
Evens
Finns
Testpop
−0.01
0.00
0.01
0.02
0.03
D = Yoruba,Testpop;Est,Fin

N.

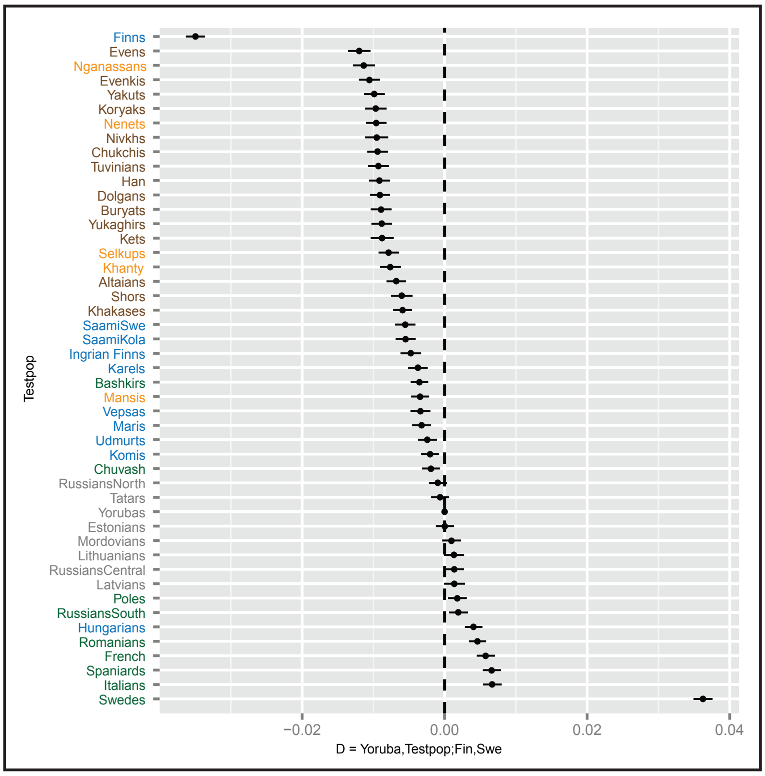
Finns
Evens
Nganassans
Evenkis
Yakuts
Koryaks
Nenets
Nivkhs
Chukchis
Tuvinians
Han
Dolgans
Buryats
Yukaghirs
Kets
Selkups
Khanty
Altaians
Shors
Khakases
SaamiSwe
SaamiKola
Ingrian Finns
Karels
Bashkirs
Mansis
Vepsas
Maris
Udmurts
Komis
Chuvash
RussiansNorth
Tatars
Yorubas
Estonians
Mordovians
Lithuanians
RussiansCentral
Latvians
Poles
RussiansSouth
Hungarians
Romanians
French
Spaniards
Italians
Swedes
Testpop
−0.02
0.00
0.02
0.04
D = Yoruba,Testpop;Fin,Swe

O.

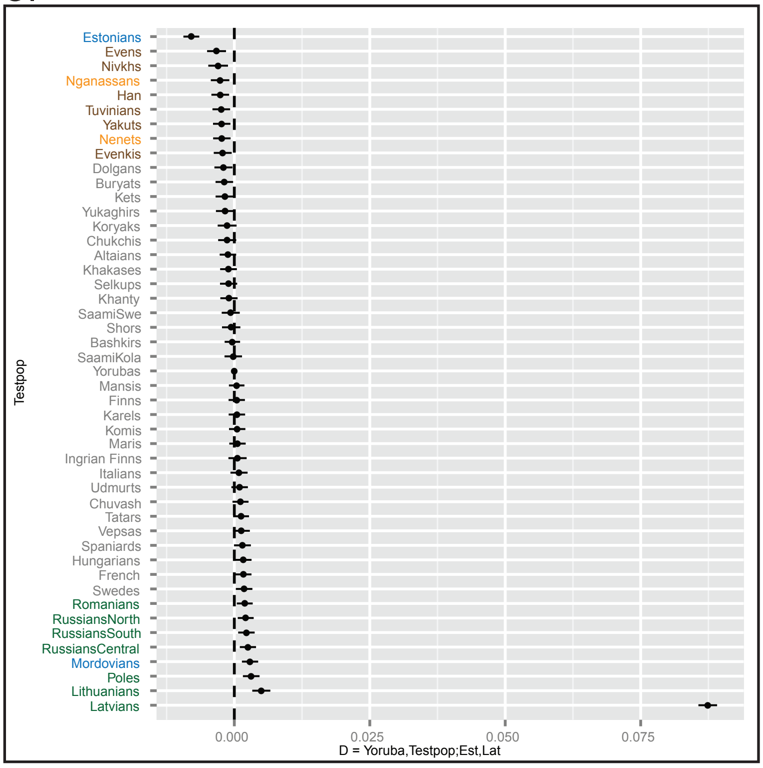
Estonians
Evens
Nivkhs
Nganassans
Han
Tuvinians
Yakuts
Nenets
Evenkis
Dolgans
Buryats
Kets
Yukaghirs
Koryaks
Chukchis
Altaians
Khakases
Selkups
Khanty
SaamiSwe
Shors
Bashkirs
SaamiKola
Yorubas
Mansis
Finns
Karels
Komis
Maris
Ingrian Finns
Italians
Udmurts
Chuvash
Tatars
Vepsas
Spaniards
Hungarians
French
Swedes
Romanians
RussiansNorth
RussiansSouth
RussiansCentral
Mordovians
Poles
Lithuanians
Latvians
Testpop
0.000
0.025
0.050
0.075
D = Yoruba,Testpop;Est,Lat

P.

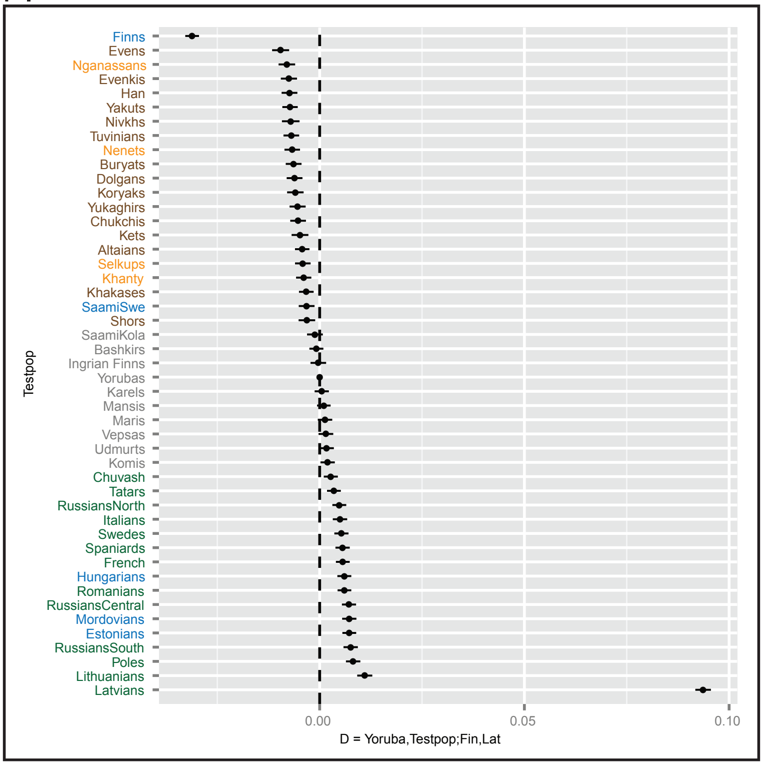
Finns
Evens
Nganassans
Evenkis
Han
Yakuts
Nivkhs
Tuvinians
Nenets
Buryats
Dolgans
Koryaks
Yukaghirs
Chukchis
Kets
Altaians
Selkups
Khanty
Khakases
SaamiSwe
Shors
SaamiKola
Bashkirs
Ingrian Finns
Yorubas
Karels
Mansis
Maris
Vepsas
Udmurts
Komis
Chuvash
Tatars
RussiansNorth
Italians
Swedes
Spaniards
French
Hungarians
Romanians
RussiansCentral
Mordovians
Estonians
RussiansSouth
Poles
Lithuanians
Latvians
Testpop
0.00
0.05
0.10
D = Yoruba,Testpop;Fin,Lat

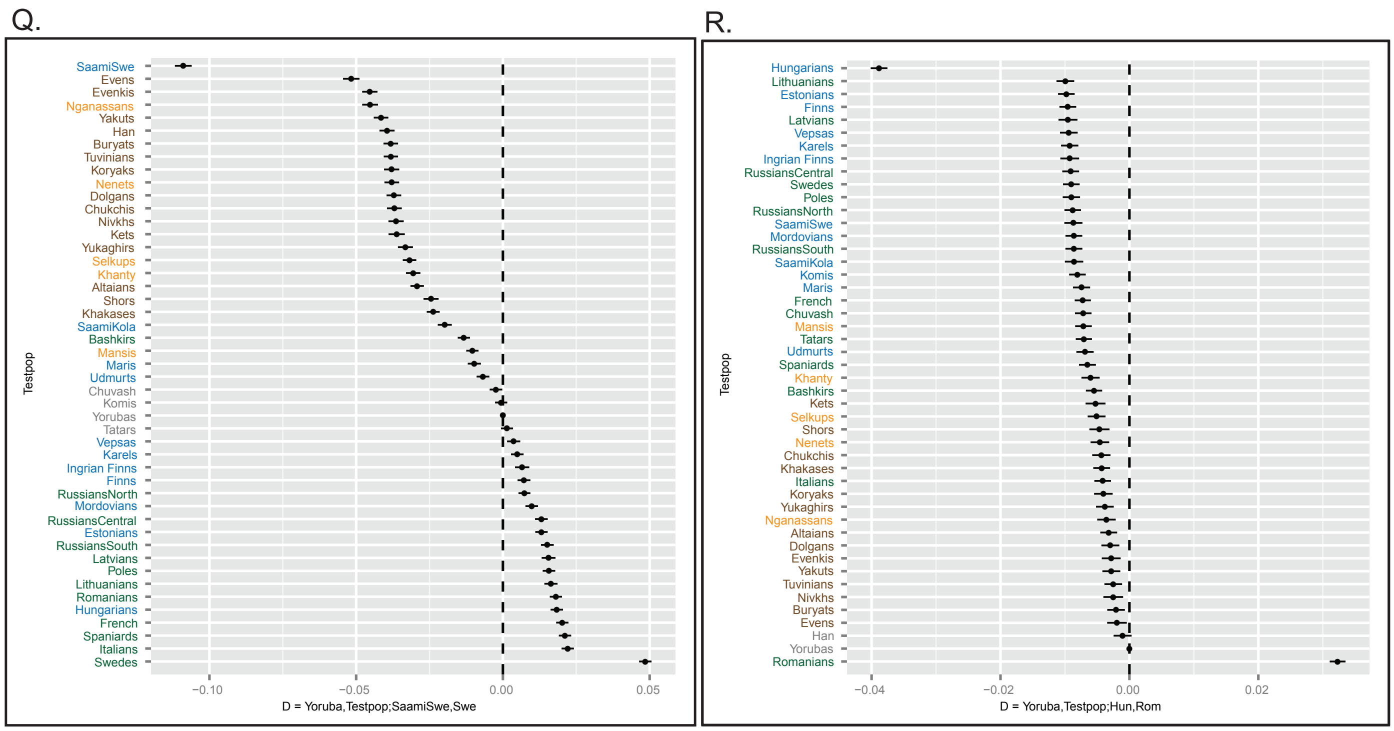

Figure S4. D-statistics calculated for the tree model in the form of D(outgroup, test population; Uralic speaking population, non-Uralic speaking population). Yorubas were used as an outgroup. Uralic speaking populations fixed in the tested model are the Saami from Sweden (SaamiSwe), Finns (Fin), Estonians (Est) and Hungarians (Hun) from Europe. The non-Uralic speaking populations fixed in the tested model are French (Fra, panels **A.-D.**), Swedes (Swe, panels **E.-H., N, Q**) and Poles (Pol, panels **I.-L.**). Panels **M.-R.** depict D-statistics opposing neighbouring population pairs Estonians-Finns (**M.**), Finns-Swedes (**N.**), Estonians-Latvians (Lat, **O.**), Finns-Latvians (**P.**), Saami and Swedes (**Q.**) and Hungarians-Romanians (Rom, **R**). The values on the Y-axis are sorted by D value. Color codes of populations showing significant deviations from D=0 (Z score ≥ 3) correspond to linguistic affinities of tested populations: blue – European Uralic speaking populations; green – European non-Uralic speaking populations; orange – West Siberian Uralic speaking populations; brown – Siberian and East Asian non-Uralic speaking populations. Grey colored labels indicates D=0 (Z score < 3), standard errors to the point estimates are shown with the black bars.

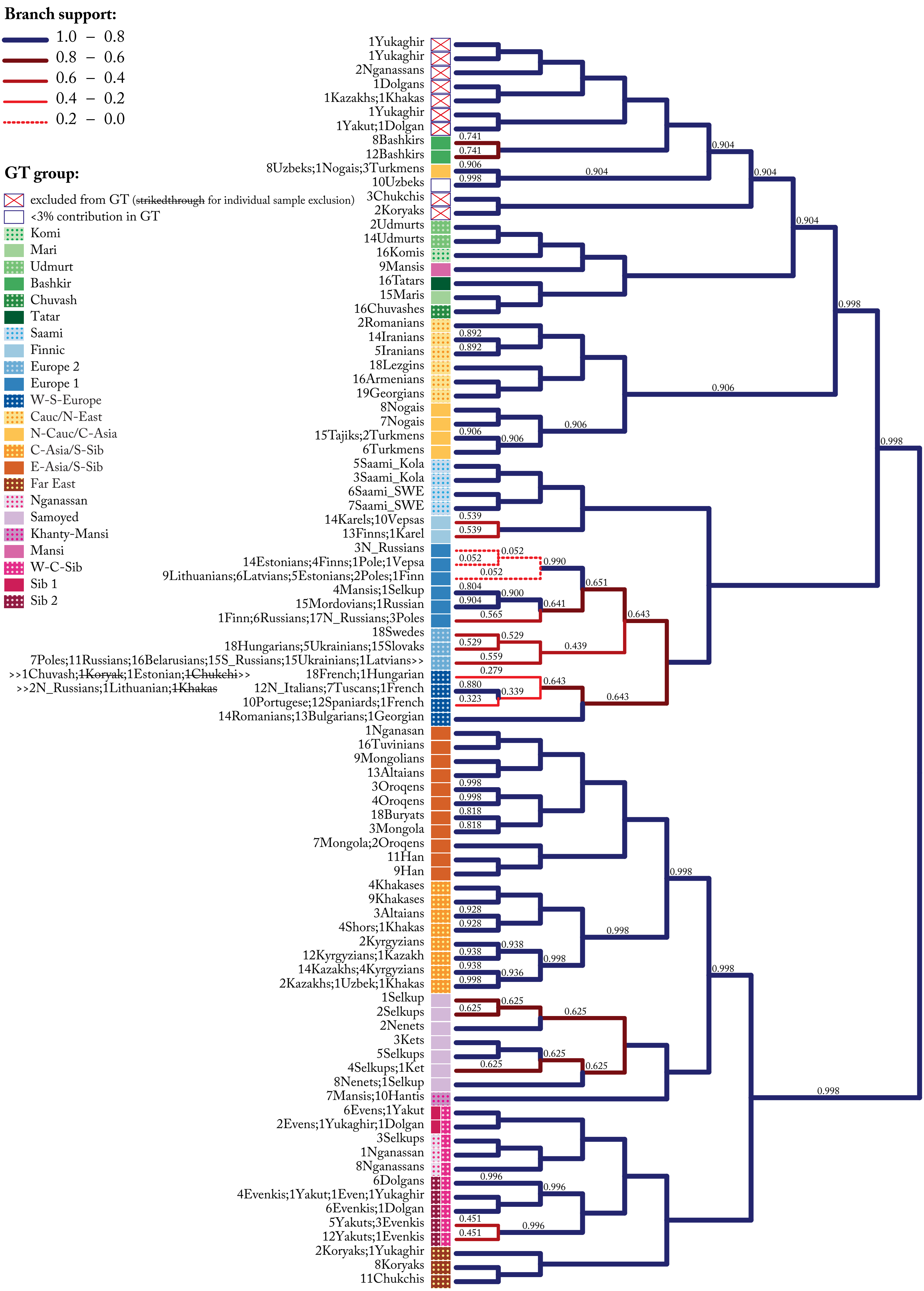
Branch support:
1.0 – 0.8
0.8 – 0.6
0.6 – 0.4
0.4 – 0.2
0.2 – 0.0
GT group:
excluded from GT (strikedthrough for individual sample exclusion)
<3% contribution in GT
Komi
Mari
Udmurt
Bashkir
Chuvash
Tatar
Saami
Finnic
Europe 2
Europe 1
W-S-Europe
Cauc/N-East
N-Cauc/C-Asia
C-Asia/S-Sib
E-Asia/S-Sib
Far East
Nganassan
Samoyed
Khanty-Mansi
Mansi
W-C-Sib
Sib 1
Sib 2
1Yukaghir
1Yukaghir
2Nganassans
1Dolgans
1Kazakhs;1Khakas
1Yukaghir
1Yakut;1Dolgan
8Bashkirs
12Bashkirs
8Uzbeks;1Nogais;3Turkmens
10Uzbeks
3Chukchis
2Koryaks
2Udmurts
14Udmurts
16Komis
9Mansis
16Tatars
15Maris
16Chuvashes
2Romanians
14Iranians
5Iranians
18Lezgins
16Armenians
19Georgians
8Nogais
7Nogais
15Tajiks;2Turkmens
6Turkmens
5Saami_Kola
3Saami_Kola
6Saami_SWE
7Saami_SWE
14Karels;10Vepsas
13Finns;1Karel
3N_Russians
14Estonians;4Finns;1Pole;1Vepsa
9Lithuanians;6Latvians;5Estonians;2Poles;1Finn
4Mansis;1Selkup
15Mordovians;1Russian
1Finn;6Russians;17N_Russians;3Poles
18Swedes
18Hungarians;5Ukrainians;15Slovaks
7Poles;11Russians;16Belarusians;15S_Russians;15Ukrainians;1Latvians>>
>>1Chuvash;1Koryak;1Estonian;1Chukchi>>
>>2N_Russians;1Lithuanian;1Khakas
18French;1Hungarian
12N_Italians;7Tuscans;1French
10Portugese;12Spaniards;1French
14Romanians;13Bulgarians;1Georgian
1Nganasan
16Tuvinians
9Mongolians
13Altaians
3Oroqens
4Oroqens
18Buryats
3Mongola
7Mongola;2Oroqens
11Han
9Han
4Khakases
9Khakases
3Altaians
4Shors;1Khakas
2Kyrgyzians
12Kyrgyzians;1Kazakh
14Kazakhs;4Kyrgyzians
2Kazakhs;1Uzbek;1Khakas
1Selkup
2Selkups
2Nenets
3Kets
5Selkups
4Selkups;1Ket
8Nenets;1Selkup
7Mansis;10Hantis
6Evens;1Yakut
2Evens;1Yukaghir;1Dolgan
3Selkups
1Nganassan
8Nganassans
6Dolgans
4Evenkis;1Yakut;1Even;1Yukaghir
6Evenkis;1Dolgan
5Yakuts;3Evenkis
12Yakuts;1Evenkis
2Koryaks;1Yukaghir
8Koryaks
11Chukchis

Figure S5. Clustering of individual samples from the comparative dataset, as inferred by fineSTRUCTURE (FS). The tree clusters individuals with similar copying vectors. Labels identify how many samples and which samples are included in each cluster. For example, '7Mongola;2Oroqens' cluster comprises of 7 Mongola and 2 Oroqen individuals. Individual tips were manually inspected and grouped for further admixture analysis in GLOBETROTTER (GT, **Figure 5**). GT groups are color-coded; legend is given on the left-hand side. For all GT runs except of analysis of admixture in the Nganasan cluster, single 'W-C-Sib' (, West and Central Siberia) group was used. For the GT analysis of the Nganasans, 'W-C-Sib' was split into three sub-groups: 'Nganassan' ( ), 'Sib 1' ( ) and 'Sib 2' ( ); see Materials and Methods for further details. FS populations (crossed out boxes,) and individual samples (strikethrough font) which show unusually high levels of admixture were excluded from GT. Single donor group, '10Uzbeks', contributed less than 3% of ancestry to all GT target populations (**Figure 5**). Line thickness of individual branches indicates statistical bootstrap support; legend is given in the top left corner.

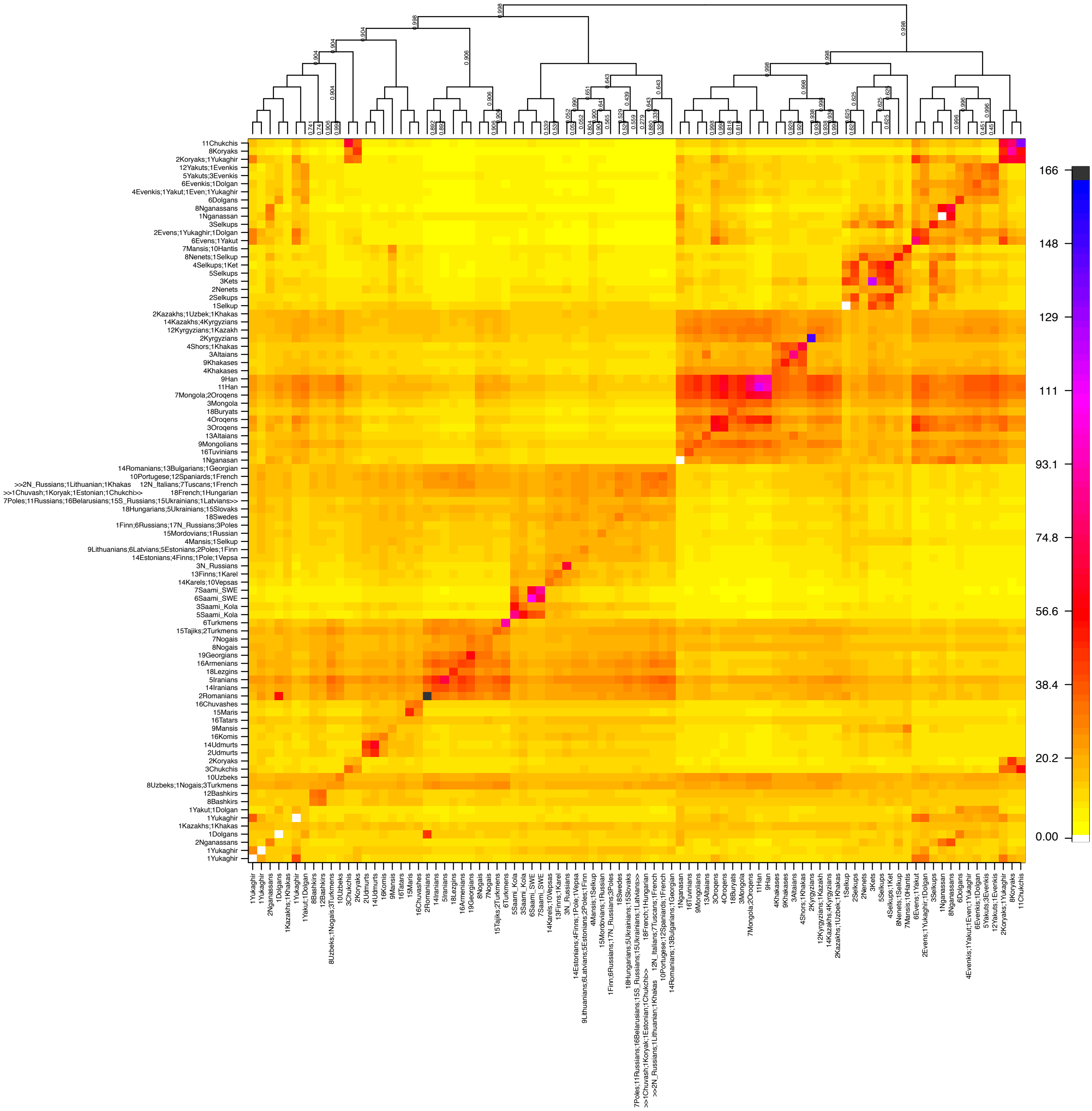

Figure S6. Results of the fineSTRUCTURE clustering analysis using copying vectors generated from chromosome painting. Each row of the heatmap is a recipient copying vector showing the number of chunks shared between the recipient cluster and every donor group (columns). Labels identify how many samples and which samples are included in each cluster. A simplified population clustering dendrogram is shown on top. A detailed dendrogram is given in Figure S5.

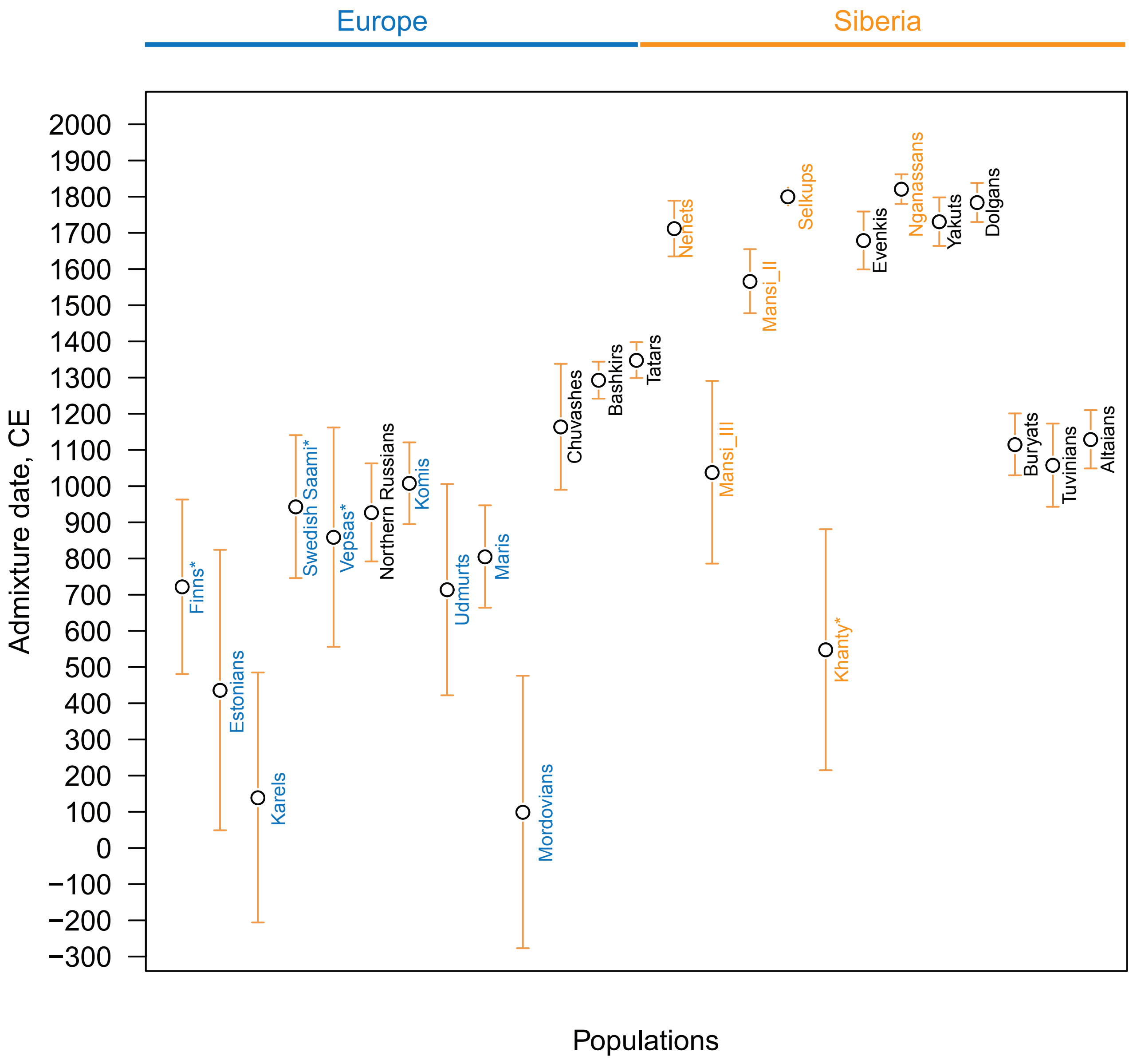

Figure S7. Admixture dates for the eastern and western components of the Uralic-speaking populations (highlighted according to geography blue for Europe and orange for Siberia) in the context of their geographical neighbors on an absolute time scale. Dates are calculated with ALDER according to decay rates of two-reference weighted linkage disequilibrium curve using the generation time of 30 years. Black circles show point estimates and error bars indicate 95% confidence intervals. Admixture dates before Common Era (CE) are shown with a negative sign. (*) indicates admixed populations with inconsistent LD curve decay rates.

A.

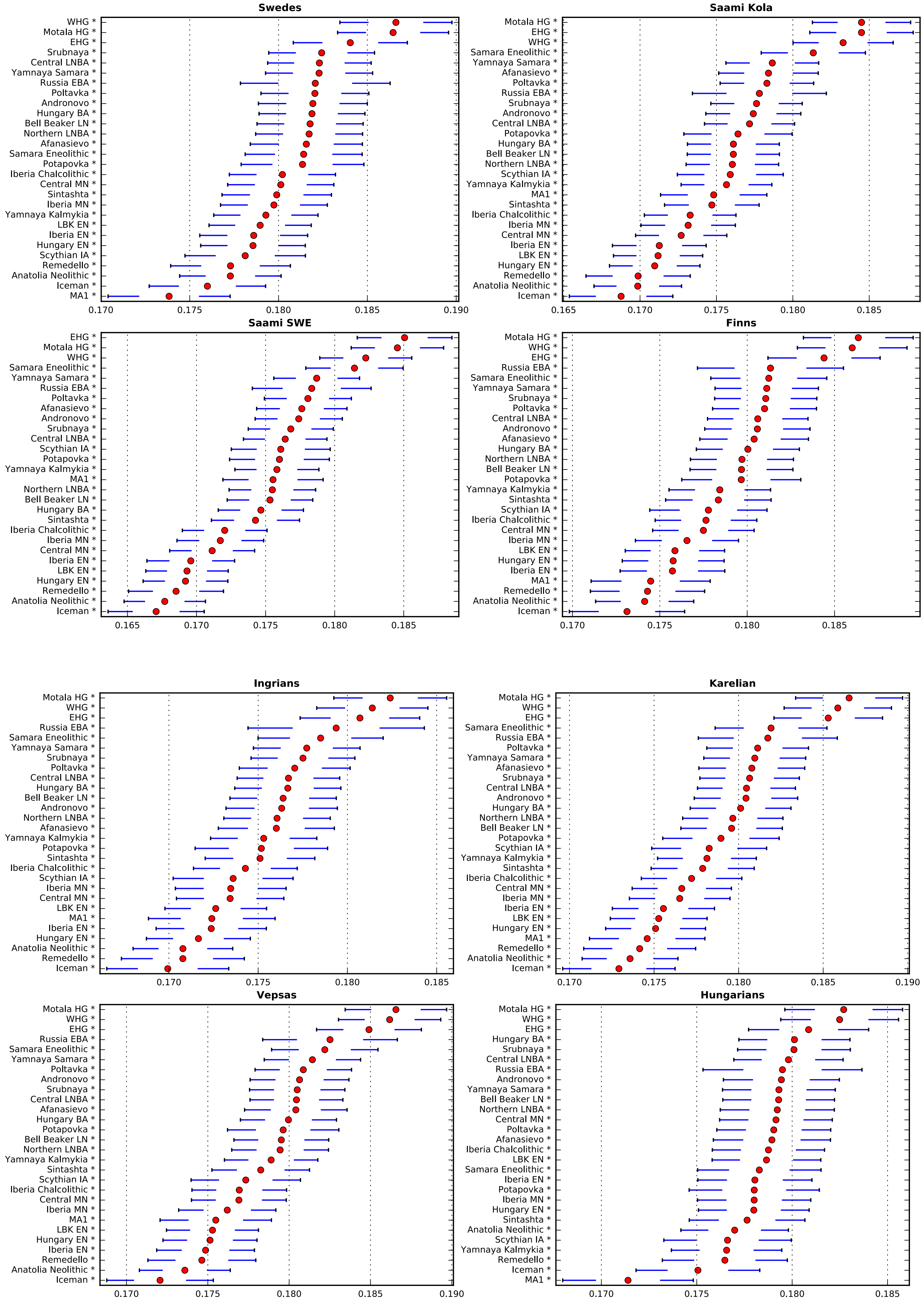

Swedes
WHG *
Motala HG *
EHG *
Srubnaya *
Central LNBA *
Yamnaya Samara *
Russia EBA *
Poltavka *
Andronovo *
Hungary BA *
Bell Beaker LN *
Northern LNBA *
Afanasievo *
Samara Eneolithic *
Potapovka *
Iberia Chalcolithic *
Central MN *
Sintashta *
Iberia MN *
Yamnaya Kalmykia *
LBK EN *
Iberia EN *
Hungary EN *
Scythian IA *
Remedello *
Anatolia Neolithic *
Iceman *
MA1 *
0.170
0.175
0.180
0.185
0.190
Saami Kola
Motala HG *
EHG *
WHG *
Samara Eneolithic *
Yamnaya Samara *
Afanasievo *
Poltavka *
Russia EBA *
Srubnaya *
Andronovo *
Central LNBA *
Potapovka *
Hungary BA *
Bell Beaker LN *
Northern LNBA *
Scythian IA *
Yamnaya Kalmykia *
MA1 *
Sintashta *
Iberia Chalcolithic *
Iberia MN *
Central MN *
Iberia EN *
LBK EN *
Hungary EN *
Remedello *
Anatolia Neolithic *
Iceman *
0.165
0.170
0.175
0.180
0.185
Saami SWE
EHG *
Motala HG *
WHG *
Samara Eneolithic *
Yamnaya Samara *
Russia EBA *
Poltavka *
Afanasievo *
Andronovo *
Srubnaya *
Central LNBA *
Scythian IA *
Potapovka *
Yamnaya Kalmykia *
MA1 *
Northern LNBA *
Bell Beaker LN *
Hungary BA *
Sintashta *
Iberia Chalcolithic *
Iberia MN *
Central MN *
Iberia EN *
LBK EN *
Hungary EN *
Remedello *
Anatolia Neolithic *
Iceman *
0.165
0.170
0.175
0.180
0.185
Finns
Motala HG *
WHG *
EHG *
Russia EBA *
Samara Eneolithic *
Yamnaya Samara *
Srubnaya *
Poltavka *
Central LNBA *
Andronovo *
Afanasievo *
Hungary BA *
Northern LNBA *
Bell Beaker LN *
Potapovka *
Yamnaya Kalmykia *
Sintashta *
Scythian IA *
Iberia Chalcolithic *
Central MN *
Iberia MN *
LBK EN *
Hungary EN *
Iberia EN *
MA1 *
Remedello *
Anatolia Neolithic *
Iceman *
0.170
0.175
0.180
0.185
Ingrians
Motala HG *
WHG *
EHG *
Russia EBA *
Samara Eneolithic *
Yamnaya Samara *
Srubnaya *
Poltavka *
Central LNBA *
Hungary BA *
Bell Beaker LN *
Andronovo *
Northern LNBA *
Afanasievo *
Yamnaya Kalmykia *
Potapovka *
Sintashta *
Iberia Chalcolithic *
Scythian IA *
Iberia MN *
Central MN *
LBK EN *
MA1 *
Iberia EN *
Hungary EN *
Anatolia Neolithic *
Remedello *
Iceman *
0.170
0.175
0.180
0.185
Karelian
Motala HG *
WHG *
EHG *
Samara Eneolithic *
Russia EBA *
Poltavka *
Yamnaya Samara *
Afanasievo *
Srubnaya *
Central LNBA *
Andronovo *
Hungary BA *
Northern LNBA *
Bell Beaker LN *
Potapovka *
Scythian IA *
Yamnaya Kalmykia *
Sintashta *
Iberia Chalcolithic *
Central MN *
Iberia MN *
Iberia EN *
LBK EN *
Hungary EN *
MA1 *
Remedello *
Anatolia Neolithic *
Iceman *
0.170
0.175
0.180
0.185
0.190
Vepsas
Motala HG *
WHG *
EHG *
Russia EBA *
Samara Eneolithic *
Yamnaya Samara *
Poltavka *
Andronovo *
Srubnaya *
Central LNBA *
Afanasievo *
Hungary BA *
Potapovka *
Bell Beaker LN *
Northern LNBA *
Yamnaya Kalmykia *
Sintashta *
Scythian IA *
Iberia Chalcolithic *
Central MN *
Iberia MN *
MA1 *
LBK EN *
Hungary EN *
Iberia EN *
Remedello *
Anatolia Neolithic *
Iceman *
0.170
0.175
0.180
0.185
0.190
Hungarians
Motala HG *
WHG *
EHG *
Hungary BA *
Srubnaya *
Central LNBA *
Russia EBA *
Andronovo *
Yamnaya Samara *
Bell Beaker LN *
Northern LNBA *
Central MN *
Poltavka *
Afanasievo *
Iberia Chalcolithic *
LBK EN *
Samara Eneolithic *
Iberia EN *
Potapovka *
Iberia MN *
Hungary EN *
Sintashta *
Anatolia Neolithic *
Scythian IA *
Yamnaya Kalmykia *
Remedello *
Iceman *
MA1 *
0.170
0.175
0.180
0.185

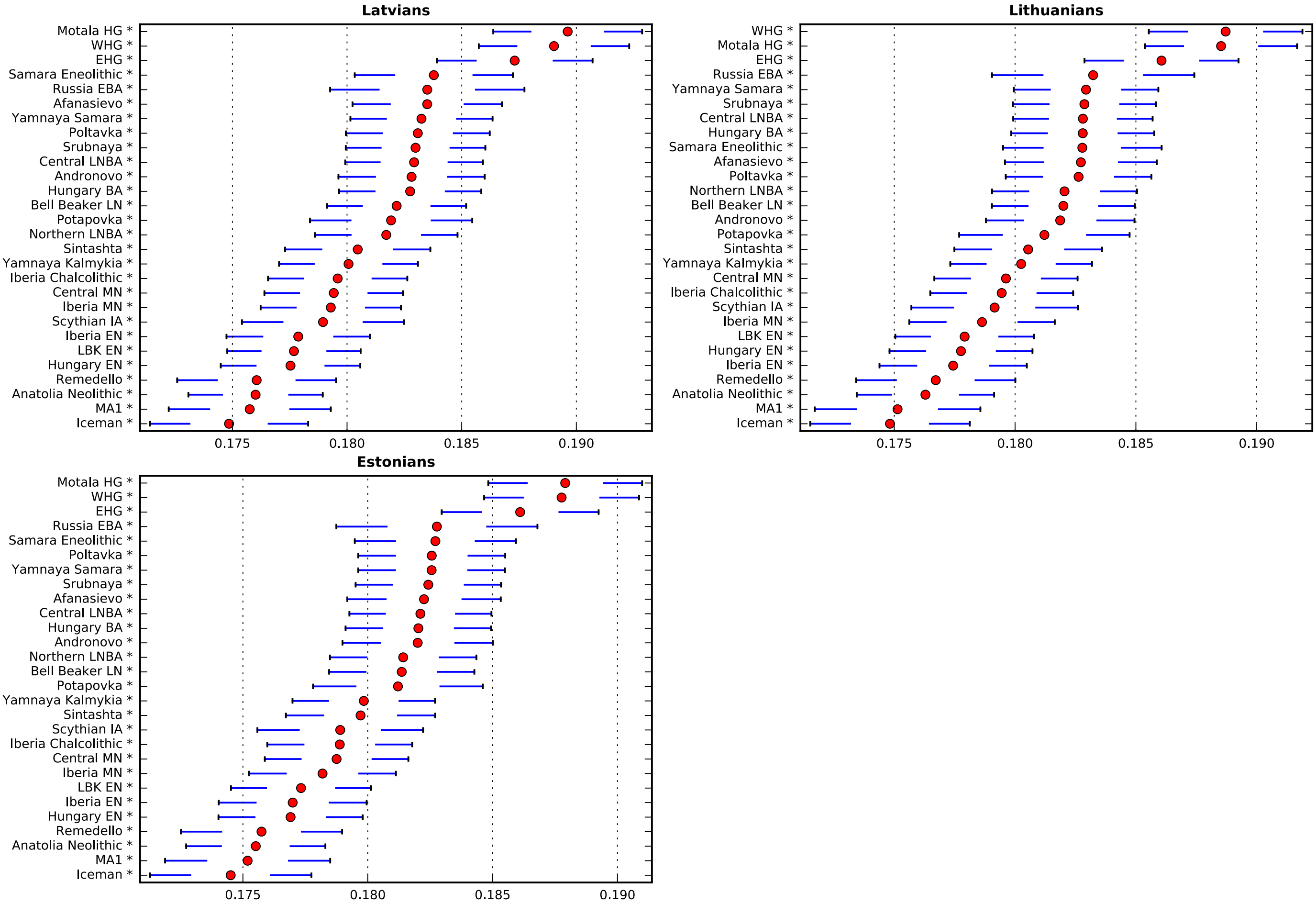
Latvians
Lithuanians
Estonians
0.175
0.180
0.185
0.190

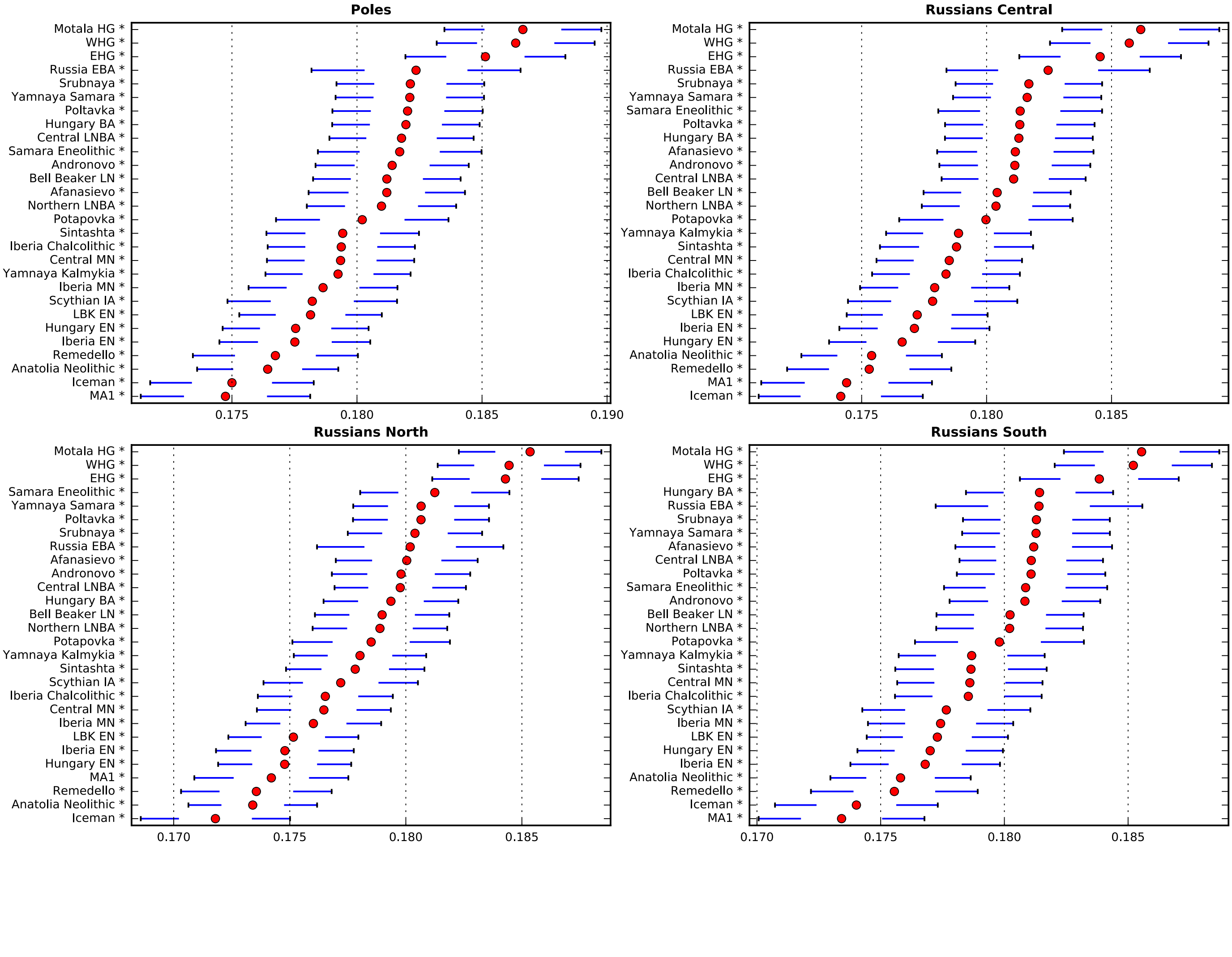
Poles
Russians Central
Russians North
Russians South
0.170
0.175
0.180
0.185
0.190

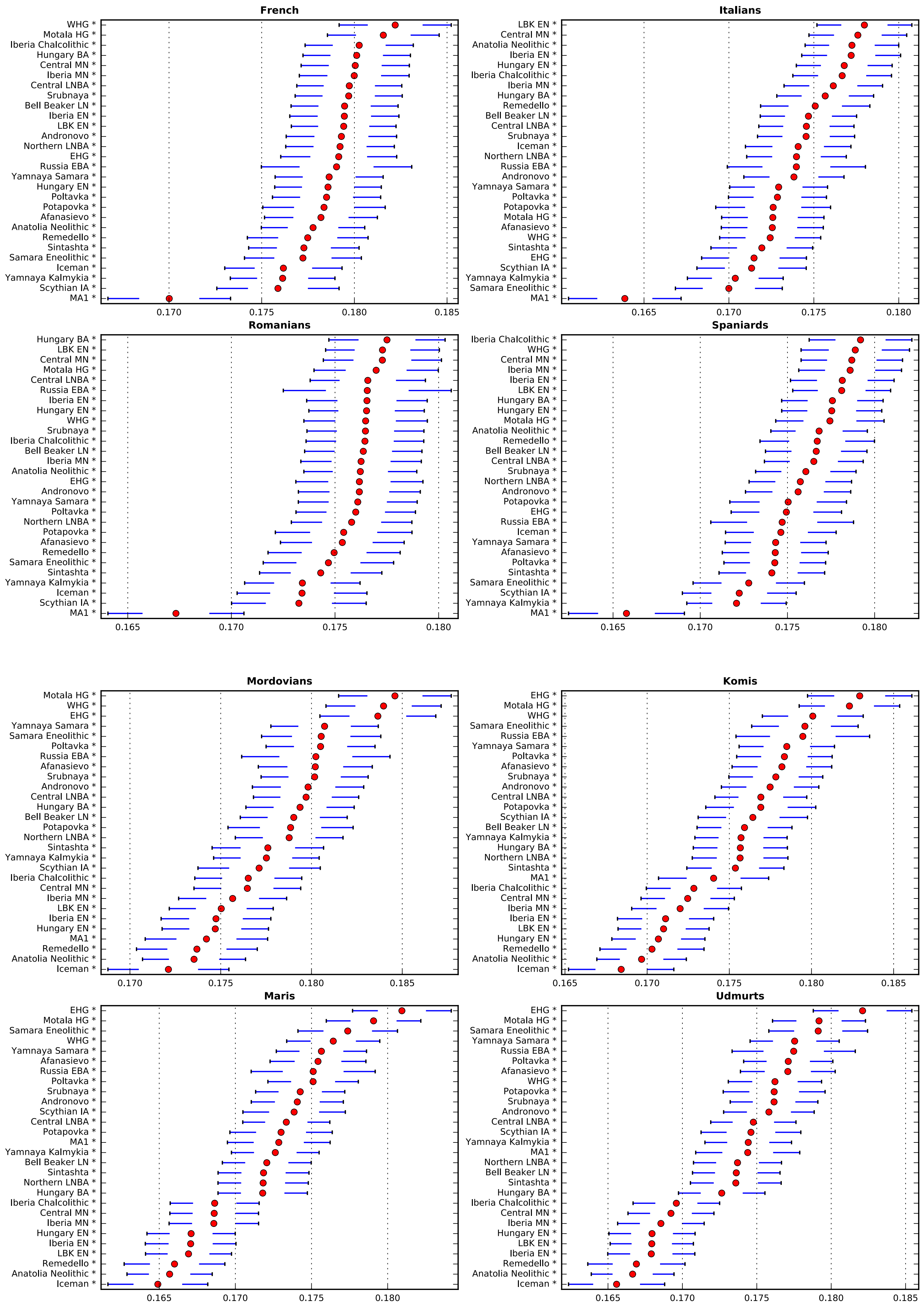
French
WHG *
Motala HG *
Iberia Chalcolithic *
Hungary BA *
Central MN *
Iberia MN *
Central LNBA *
Srubnaya *
Bell Beaker LN *
Iberia EN *
LBK EN *
Andronovo *
Northern LNBA *
EHG *
Russia EBA *
Yamnaya Samara *
Hungary EN *
Poltavka *
Potapovka *
Afanasievo *
Anatolia Neolithic *
Remedello *
Sintashta *
Samara Eneolithic *
Iceman *
Yamnaya Kalmykia *
Scythian IA *
MA1 *
0.170
0.175
0.180
0.185
Italians
LBK EN *
Central MN *
Anatolia Neolithic *
Iberia EN *
Hungary EN *
Iberia Chalcolithic *
Iberia MN *
Hungary BA *
Remedello *
Bell Beaker LN *
Central LNBA *
Srubnaya *
Iceman *
Northern LNBA *
Russia EBA *
Andronovo *
Yamnaya Samara *
Poltavka *
Potapovka *
Motala HG *
Afanasievo *
WHG *
Sintashta *
EHG *
Scythian IA *
Yamnaya Kalmykia *
Samara Eneolithic *
MA1 *
0.165
0.170
0.175
0.180
Romanians
Hungary BA *
LBK EN *
Central MN *
Motala HG *
Central LNBA *
Russia EBA *
Iberia EN *
Hungary EN *
WHG *
Srubnaya *
Iberia Chalcolithic *
Bell Beaker LN *
Iberia MN *
Anatolia Neolithic *
EHG *
Andronovo *
Yamnaya Samara *
Poltavka *
Northern LNBA *
Potapovka *
Afanasievo *
Remedello *
Samara Eneolithic *
Sintashta *
Yamnaya Kalmykia *
Iceman *
Scythian IA *
MA1 *
0.165
0.170
0.175
0.180
Spaniards
Iberia Chalcolithic *
WHG *
Central MN *
Iberia MN *
Iberia EN *
LBK EN *
Hungary BA *
Hungary EN *
Motala HG *
Anatolia Neolithic *
Remedello *
Bell Beaker LN *
Central LNBA *
Srubnaya *
Northern LNBA *
Andronovo *
Potapovka *
EHG *
Russia EBA *
Iceman *
Yamnaya Samara *
Afanasievo *
Poltavka *
Sintashta *
Samara Eneolithic *
Scythian IA *
Yamnaya Kalmykia *
MA1 *
0.165
0.170
0.175
0.180
Mordovians
Motala HG *
WHG *
EHG *
Yamnaya Samara *
Samara Eneolithic *
Poltavka *
Russia EBA *
Afanasievo *
Srubnaya *
Andronovo *
Central LNBA *
Hungary BA *
Bell Beaker LN *
Potapovka *
Northern LNBA *
Sintashta *
Yamnaya Kalmykia *
Scythian IA *
Iberia Chalcolithic *
Central MN *
Iberia MN *
LBK EN *
Iberia EN *
Hungary EN *
MA1 *
Remedello *
Anatolia Neolithic *
Iceman *
0.170
0.175
0.180
0.185
Komis
EHG *
Motala HG *
WHG *
Samara Eneolithic *
Russia EBA *
Yamnaya Samara *
Poltavka *
Afanasievo *
Srubnaya *
Andronovo *
Central LNBA *
Potapovka *
Scythian IA *
Bell Beaker LN *
Yamnaya Kalmykia *
Hungary BA *
Northern LNBA *
Sintashta *
MA1 *
Iberia Chalcolithic *
Central MN *
Iberia MN *
Iberia EN *
LBK EN *
Hungary EN *
Remedello *
Anatolia Neolithic *
Iceman *
0.165
0.170
0.175
0.180
0.185
Maris
EHG *
Motala HG *
Samara Eneolithic *
WHG *
Yamnaya Samara *
Afanasievo *
Russia EBA *
Poltavka *
Srubnaya *
Andronovo *
Scythian IA *
Central LNBA *
Potapovka *
MA1 *
Yamnaya Kalmykia *
Bell Beaker LN *
Sintashta *
Northern LNBA *
Hungary BA *
Iberia Chalcolithic *
Central MN *
Iberia MN *
Hungary EN *
Iberia EN *
LBK EN *
Remedello *
Anatolia Neolithic *
Iceman *
0.165
0.170
0.175
0.180
Udmurts
EHG *
Motala HG *
Samara Eneolithic *
Yamnaya Samara *
Russia EBA *
Poltavka *
Afanasievo *
WHG *
Potapovka *
Srubnaya *
Andronovo *
Central LNBA *
Scythian IA *
Yamnaya Kalmykia *
MA1 *
Northern LNBA *
Bell Beaker LN *
Sintashta *
Hungary BA *
Iberia Chalcolithic *
Central MN *
Iberia MN *
Hungary EN *
LBK EN *
Iberia EN *
Remedello *
Anatolia Neolithic *
Iceman *
0.165
0.170
0.175
0.180
0.185

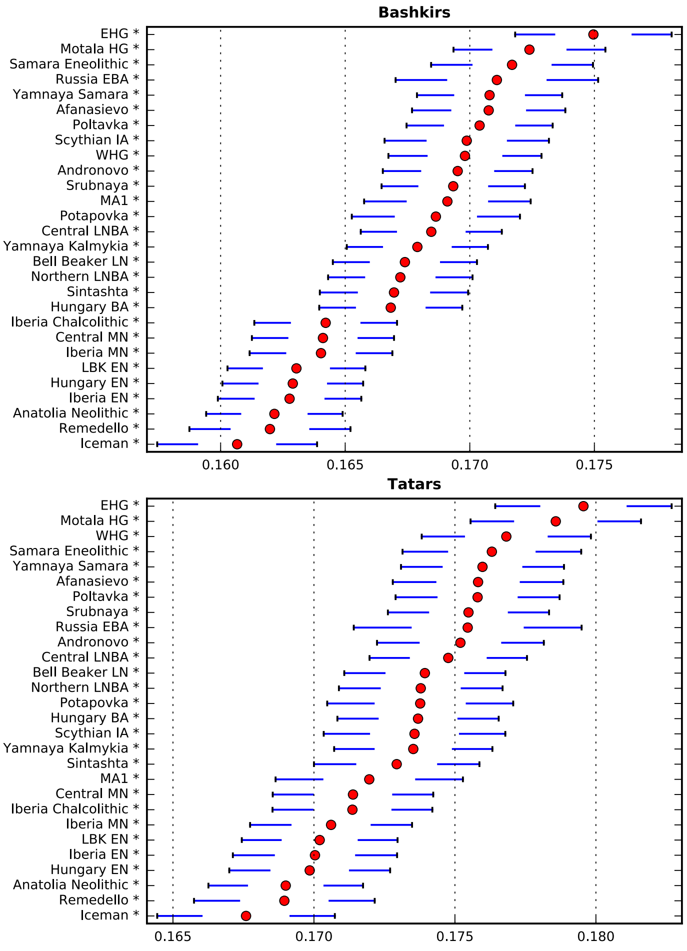
Bashkirs
EHG *
Motala HG *
Samara Eneolithic *
Russia EBA *
Yamnaya Samara *
Afanasievo *
Poltavka *
Scythian IA *
WHG *
Andronovo *
Srubnaya *
MA1 *
Potapovka *
Central LNBA *
Yamnaya Kalmykia *
Bell Beaker LN *
Northern LNBA *
Sintashta *
Hungary BA *
Iberia Chalcolithic *
Central MN *
Iberia MN *
LBK EN *
Hungary EN *
Iberia EN *
Anatolia Neolithic *
Remedello *
Iceman *
0.160
0.165
0.170
0.175
Chuvash
EHG *
Motala HG *
WHG *
Samara Eneolithic *
Yamnaya Samara *
Poltavka *
Russia EBA *
Afanasievo *
Srubnaya *
Andronovo *
Potapovka *
Central LNBA *
Bell Beaker LN *
Scythian IA *
Hungary BA *
Northern LNBA *
Yamnaya Kalmykia *
Sintashta *
MA1 *
Iberia Chalcolithic *
Central MN *
Iberia MN *
Hungary EN *
LBK EN *
Iberia EN *
Remedello *
Anatolia Neolithic *
Iceman *
0.165
0.170
0.175
0.180
Tatars
EHG *
Motala HG *
WHG *
Samara Eneolithic *
Yamnaya Samara *
Afanasievo *
Poltavka *
Srubnaya *
Russia EBA *
Andronovo *
Central LNBA *
Bell Beaker LN *
Northern LNBA *
Potapovka *
Hungary BA *
Scythian IA *
Yamnaya Kalmykia *
Sintashta *
MA1 *
Central MN *
Iberia Chalcolithic *
Iberia MN *
LBK EN *
Iberia EN *
Hungary EN *
Anatolia Neolithic *
Remedello *
Iceman *
0.165
0.170
0.175
0.180

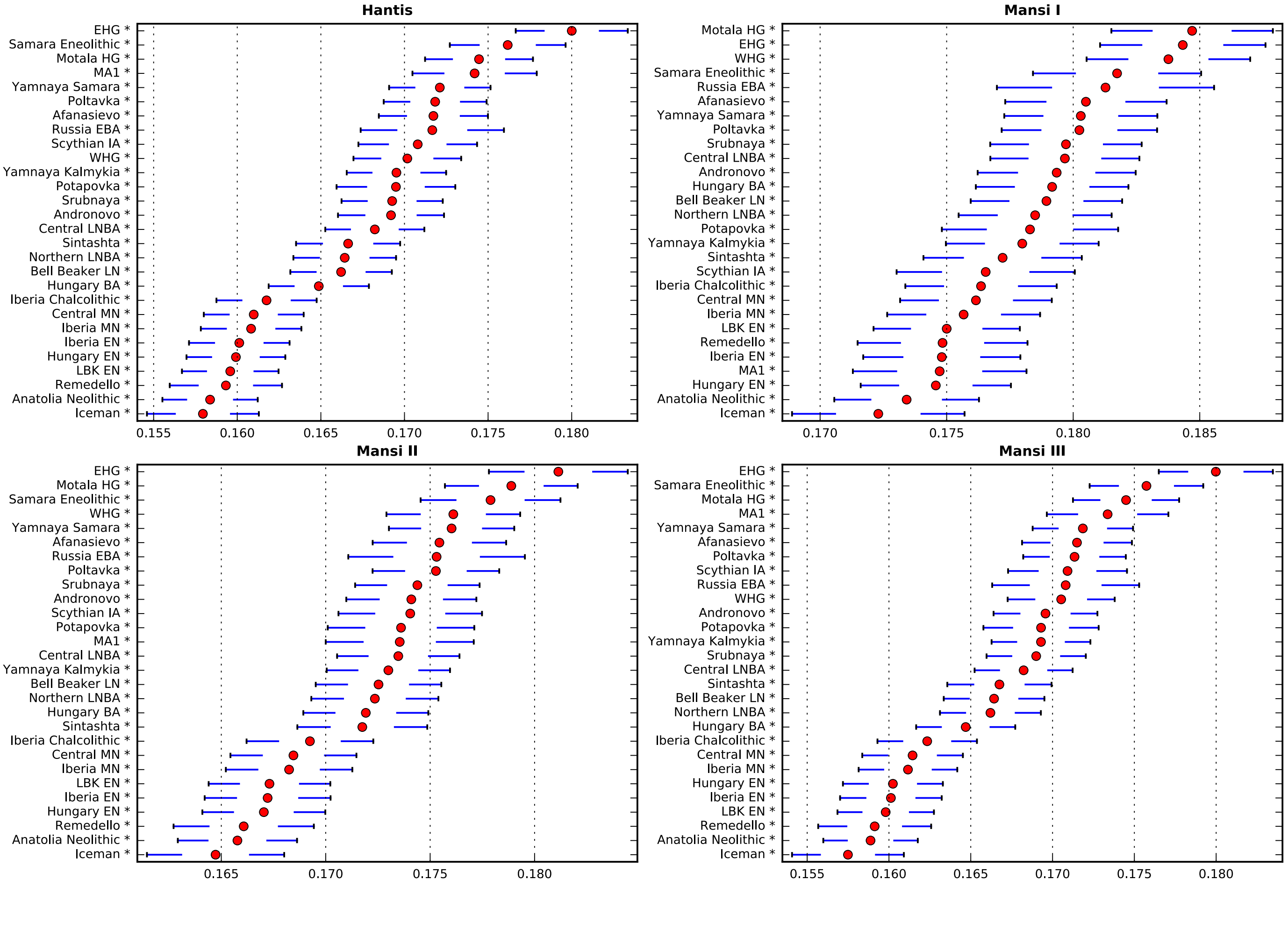
Hantis
EHG *
Samara Eneolithic *
Motala HG *
MA1 *
Yamnaya Samara *
Poltavka *
Afanasievo *
Russia EBA *
Scythian IA *
WHG *
Yamnaya Kalmykia *
Potapovka *
Srubnaya *
Andronovo *
Central LNBA *
Sintashta *
Northern LNBA *
Bell Beaker LN *
Hungary BA *
Iberia Chalcolithic *
Central MN *
Iberia MN *
Iberia EN *
Hungary EN *
LBK EN *
Remedello *
Anatolia Neolithic *
Iceman *
0.155
0.160
0.165
0.170
0.175
0.180
Mansi I
Motala HG *
EHG *
WHG *
Samara Eneolithic *
Russia EBA *
Afanasievo *
Yamnaya Samara *
Poltavka *
Srubnaya *
Central LNBA *
Andronovo *
Hungary BA *
Bell Beaker LN *
Northern LNBA *
Potapovka *
Yamnaya Kalmykia *
Sintashta *
Scythian IA *
Iberia Chalcolithic *
Central MN *
Iberia MN *
LBK EN *
Remedello *
Iberia EN *
MA1 *
Hungary EN *
Anatolia Neolithic *
Iceman *
0.170
0.175
0.180
0.185
Mansi II
EHG *
Motala HG *
Samara Eneolithic *
WHG *
Yamnaya Samara *
Afanasievo *
Russia EBA *
Poltavka *
Srubnaya *
Andronovo *
Scythian IA *
Potapovka *
MA1 *
Central LNBA *
Yamnaya Kalmykia *
Bell Beaker LN *
Northern LNBA *
Hungary BA *
Sintashta *
Iberia Chalcolithic *
Central MN *
Iberia MN *
LBK EN *
Iberia EN *
Hungary EN *
Remedello *
Anatolia Neolithic *
Iceman *
0.165
0.170
0.175
0.180
Mansi III
EHG *
Samara Eneolithic *
Motala HG *
MA1 *
Yamnaya Samara *
Afanasievo *
Poltavka *
Scythian IA *
Russia EBA *
WHG *
Andronovo *
Potapovka *
Yamnaya Kalmykia *
Srubnaya *
Central LNBA *
Sintashta *
Bell Beaker LN *
Northern LNBA *
Hungary BA *
Iberia Chalcolithic *
Central MN *
Iberia MN *
Hungary EN *
Iberia EN *
LBK EN *
Remedello *
Anatolia Neolithic *
Iceman *
0.155
0.160
0.165
0.170
0.175
0.180

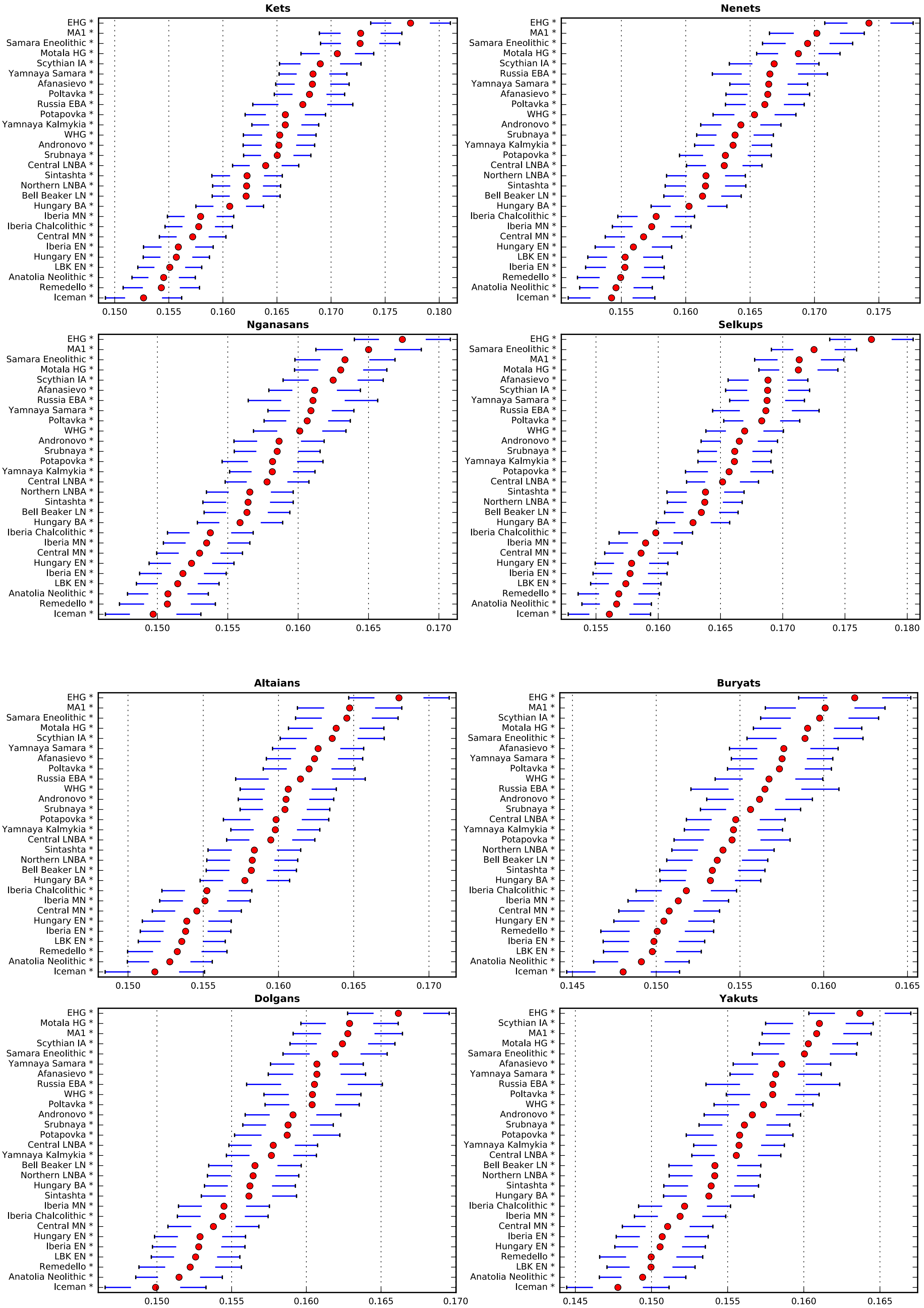
Kets
EHG *
MA1 *
Samara Eneolithic *
Motala HG *
Scythian IA *
Yamnaya Samara *
Afanasievo *
Poltavka *
Russia EBA *
Potapovka *
Yamnaya Kalmykia *
WHG *
Andronovo *
Srubnaya *
Central LNBA *
Sintashta *
Northern LNBA *
Bell Beaker LN *
Hungary BA *
Iberia MN *
Iberia Chalcolithic *
Central MN *
Iberia EN *
Hungary EN *
LBK EN *
Anatolia Neolithic *
Remedello *
Iceman *
0.150 0.155 0.160 0.165 0.170 0.175 0.180
Nenets
EHG *
MA1 *
Samara Eneolithic *
Motala HG *
Scythian IA *
Russia EBA *
Yamnaya Samara *
Afanasievo *
Poltavka *
WHG *
Andronovo *
Srubnaya *
Yamnaya Kalmykia *
Potapovka *
Central LNBA *
Northern LNBA *
Sintashta *
Bell Beaker LN *
Hungary BA *
Iberia Chalcolithic *
Iberia MN *
Central MN *
Hungary EN *
LBK EN *
Iberia EN *
Remedello *
Anatolia Neolithic *
Iceman *
0.155 0.160 0.165 0.170 0.175
Nganasans
EHG *
MA1 *
Samara Eneolithic *
Motala HG *
Scythian IA *
Afanasievo *
Russia EBA *
Yamnaya Samara *
Poltavka *
WHG *
Andronovo *
Srubnaya *
Potapovka *
Yamnaya Kalmykia *
Central LNBA *
Northern LNBA *
Sintashta *
Bell Beaker LN *
Hungary BA *
Iberia Chalcolithic *
Iberia MN *
Central MN *
Hungary EN *
Iberia EN *
LBK EN *
Anatolia Neolithic *
Remedello *
Iceman *
0.150 0.155 0.160 0.165 0.170
Selkups
EHG *
Samara Eneolithic *
MA1 *
Motala HG *
Afanasievo *
Scythian IA *
Yamnaya Samara *
Russia EBA *
Poltavka *
WHG *
Andronovo *
Srubnaya *
Yamnaya Kalmykia *
Potapovka *
Central LNBA *
Sintashta *
Northern LNBA *
Bell Beaker LN *
Hungary BA *
Iberia Chalcolithic *
Iberia MN *
Central MN *
Hungary EN *
Iberia EN *
LBK EN *
Remedello *
Anatolia Neolithic *
Iceman *
0.155 0.160 0.165 0.170 0.175 0.180
Altaians
EHG *
MA1 *
Samara Eneolithic *
Motala HG *
Scythian IA *
Yamnaya Samara *
Afanasievo *
Poltavka *
Russia EBA *
WHG *
Andronovo *
Srubnaya *
Potapovka *
Yamnaya Kalmykia *
Central LNBA *
Sintashta *
Northern LNBA *
Bell Beaker LN *
Hungary BA *
Iberia Chalcolithic *
Iberia MN *
Central MN *
Hungary EN *
Iberia EN *
LBK EN *
Remedello *
Anatolia Neolithic *
Iceman *
0.150 0.155 0.160 0.165 0.170
Buryats
EHG *
MA1 *
Scythian IA *
Motala HG *
Samara Eneolithic *
Afanasievo *
Yamnaya Samara *
Poltavka *
WHG *
Russia EBA *
Andronovo *
Srubnaya *
Central LNBA *
Yamnaya Kalmykia *
Potapovka *
Northern LNBA *
Bell Beaker LN *
Sintashta *
Hungary BA *
Iberia Chalcolithic *
Iberia MN *
Central MN *
Hungary EN *
Remedello *
Iberia EN *
LBK EN *
Anatolia Neolithic *
Iceman *
0.145 0.150 0.155 0.160 0.165
Dolgans
EHG *
Motala HG *
MA1 *
Scythian IA *
Samara Eneolithic *
Yamnaya Samara *
Afanasievo *
Russia EBA *
WHG *
Poltavka *
Andronovo *
Srubnaya *
Potapovka *
Central LNBA *
Yamnaya Kalmykia *
Bell Beaker LN *
Northern LNBA *
Hungary BA *
Sintashta *
Iberia MN *
Iberia Chalcolithic *
Central MN *
Hungary EN *
Iberia EN *
LBK EN *
Remedello *
Anatolia Neolithic *
Iceman *
0.150 0.155 0.160 0.165 0.170
Yakuts
EHG *
Scythian IA *
MA1 *
Motala HG *
Samara Eneolithic *
Afanasievo *
Yamnaya Samara *
Russia EBA *
Poltavka *
WHG *
Andronovo *
Srubnaya *
Potapovka *
Yamnaya Kalmykia *
Central LNBA *
Bell Beaker LN *
Northern LNBA *
Sintashta *
Hungary BA *
Iberia Chalcolithic *
Iberia MN *
Central MN *
Iberia EN *
Hungary EN *
Remedello *
LBK EN *
Anatolia Neolithic *
Iceman *
0.145 0.150 0.155 0.160 0.165

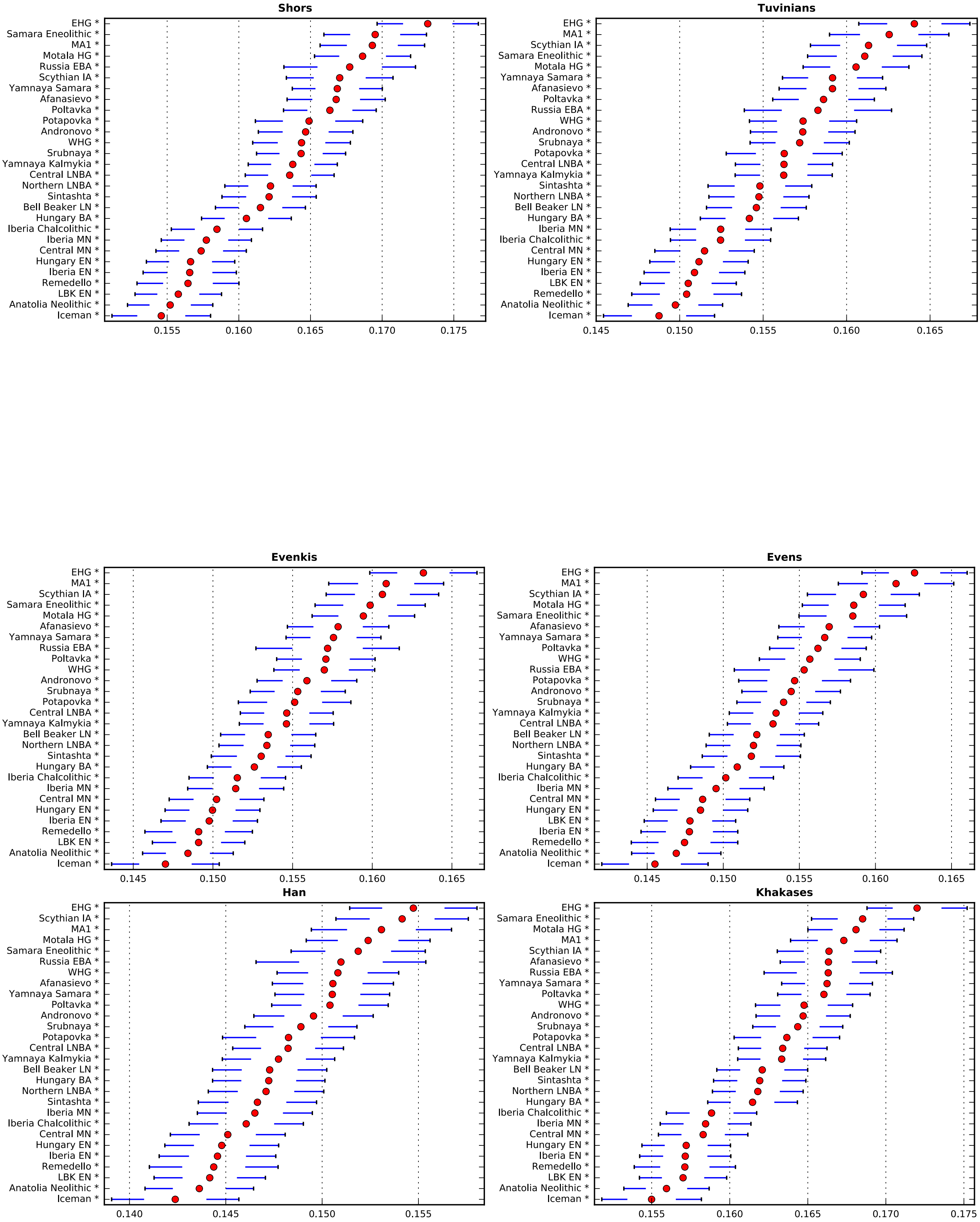
Shors
EHG *
Samara Eneolithic *
MA1 *
Motala HG *
Russia EBA *
Scythian IA *
Yamnaya Samara *
Afanasievo *
Poltavka *
Potapovka *
Andronovo *
WHG *
Srubnaya *
Yamnaya Kalmykia *
Central LNBA *
Northern LNBA *
Sintashta *
Bell Beaker LN *
Hungary BA *
Iberia Chalcolithic *
Iberia MN *
Central MN *
Hungary EN *
Iberia EN *
Remedello *
LBK EN *
Anatolia Neolithic *
Iceman *
0.155
0.160
0.165
0.170
0.175
Tuvinians
EHG *
MA1 *
Scythian IA *
Samara Eneolithic *
Motala HG *
Yamnaya Samara *
Afanasievo *
Poltavka *
Russia EBA *
WHG *
Andronovo *
Srubnaya *
Potapovka *
Central LNBA *
Yamnaya Kalmykia *
Sintashta *
Northern LNBA *
Bell Beaker LN *
Hungary BA *
Iberia MN *
Iberia Chalcolithic *
Central MN *
Hungary EN *
Iberia EN *
LBK EN *
Remedello *
Anatolia Neolithic *
Iceman *
0.145
0.150
0.155
0.160
0.165
Evenkis
EHG *
MA1 *
Scythian IA *
Samara Eneolithic *
Motala HG *
Afanasievo *
Yamnaya Samara *
Russia EBA *
Poltavka *
WHG *
Andronovo *
Srubnaya *
Potapovka *
Central LNBA *
Yamnaya Kalmykia *
Bell Beaker LN *
Northern LNBA *
Sintashta *
Hungary BA *
Iberia Chalcolithic *
Iberia MN *
Central MN *
Hungary EN *
Iberia EN *
Remedello *
LBK EN *
Anatolia Neolithic *
Iceman *
0.145
0.150
0.155
0.160
0.165
Evens
EHG *
MA1 *
Scythian IA *
Motala HG *
Samara Eneolithic *
Afanasievo *
Yamnaya Samara *
Poltavka *
WHG *
Russia EBA *
Potapovka *
Andronovo *
Srubnaya *
Yamnaya Kalmykia *
Central LNBA *
Bell Beaker LN *
Northern LNBA *
Sintashta *
Hungary BA *
Iberia Chalcolithic *
Iberia MN *
Central MN *
Hungary EN *
LBK EN *
Iberia EN *
Remedello *
Anatolia Neolithic *
Iceman *
0.145
0.150
0.155
0.160
0.165
Han
EHG *
Scythian IA *
MA1 *
Motala HG *
Samara Eneolithic *
Russia EBA *
WHG *
Afanasievo *
Yamnaya Samara *
Poltavka *
Andronovo *
Srubnaya *
Potapovka *
Central LNBA *
Yamnaya Kalmykia *
Bell Beaker LN *
Hungary BA *
Northern LNBA *
Sintashta *
Iberia MN *
Iberia Chalcolithic *
Central MN *
Hungary EN *
Iberia EN *
Remedello *
LBK EN *
Anatolia Neolithic *
Iceman *
0.140
0.145
0.150
0.155
Khakases
EHG *
Samara Eneolithic *
Motala HG *
MA1 *
Scythian IA *
Afanasievo *
Russia EBA *
Yamnaya Samara *
Poltavka *
WHG *
Andronovo *
Srubnaya *
Potapovka *
Central LNBA *
Yamnaya Kalmykia *
Bell Beaker LN *
Sintashta *
Northern LNBA *
Hungary BA *
Iberia Chalcolithic *
Iberia MN *
Central MN *
Hungary EN *
Iberia EN *
Remedello *
LBK EN *
Anatolia Neolithic *
Iceman *
0.155
0.160
0.165
0.170
0.175

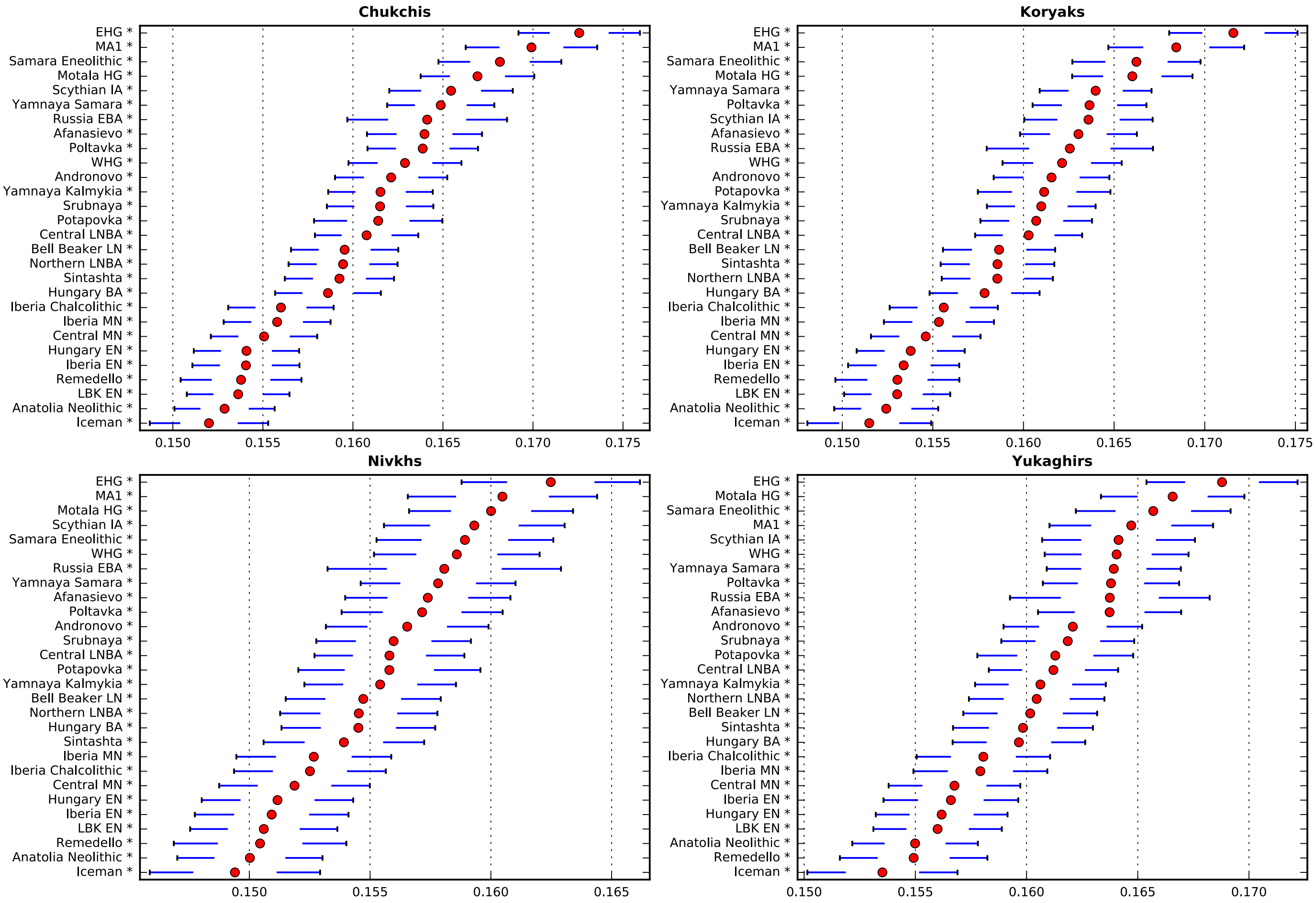
Chukchis
EHG *
MA1 *
Samara Eneolithic *
Motala HG *
Scythian IA *
Yamnaya Samara *
Russia EBA *
Afanasievo *
Poltavka *
WHG *
Andronovo *
Yamnaya Kalmykia *
Srubnaya *
Potapovka *
Central LNBA *
Bell Beaker LN *
Northern LNBA *
Sintashta *
Hungary BA *
Iberia Chalcolithic *
Iberia MN *
Central MN *
Hungary EN *
Iberia EN *
Remedello *
LBK EN *
Anatolia Neolithic *
Iceman *
0.150
0.155
0.160
0.165
0.170
0.175
Koryaks
EHG *
MA1 *
Samara Eneolithic *
Motala HG *
Yamnaya Samara *
Poltavka *
Scythian IA *
Afanasievo *
Russia EBA *
WHG *
Andronovo *
Potapovka *
Yamnaya Kalmykia *
Srubnaya *
Central LNBA *
Bell Beaker LN *
Sintashta *
Northern LNBA *
Hungary BA *
Iberia Chalcolithic *
Iberia MN *
Central MN *
Hungary EN *
Iberia EN *
Remedello *
LBK EN *
Anatolia Neolithic *
Iceman *
0.150
0.155
0.160
0.165
0.170
0.175
Nivkhs
EHG *
MA1 *
Motala HG *
Scythian IA *
Samara Eneolithic *
WHG *
Russia EBA *
Yamnaya Samara *
Afanasievo *
Poltavka *
Andronovo *
Srubnaya *
Central LNBA *
Potapovka *
Yamnaya Kalmykia *
Bell Beaker LN *
Northern LNBA *
Hungary BA *
Sintashta *
Iberia MN *
Iberia Chalcolithic *
Central MN *
Hungary EN *
Iberia EN *
LBK EN *
Remedello *
Anatolia Neolithic *
Iceman *
0.150
0.155
0.160
0.165
Yukaghirs
EHG *
Motala HG *
Samara Eneolithic *
MA1 *
Scythian IA *
WHG *
Yamnaya Samara *
Poltavka *
Russia EBA *
Afanasievo *
Andronovo *
Srubnaya *
Potapovka *
Central LNBA *
Yamnaya Kalmykia *
Northern LNBA *
Bell Beaker LN *
Sintashta *
Hungary BA *
Iberia Chalcolithic *
Iberia MN *
Central MN *
Iberia EN *
Hungary EN *
LBK EN *
Anatolia Neolithic *
Remedello *
Iceman *
0.150
0.155
0.160
0.165
0.170

B.

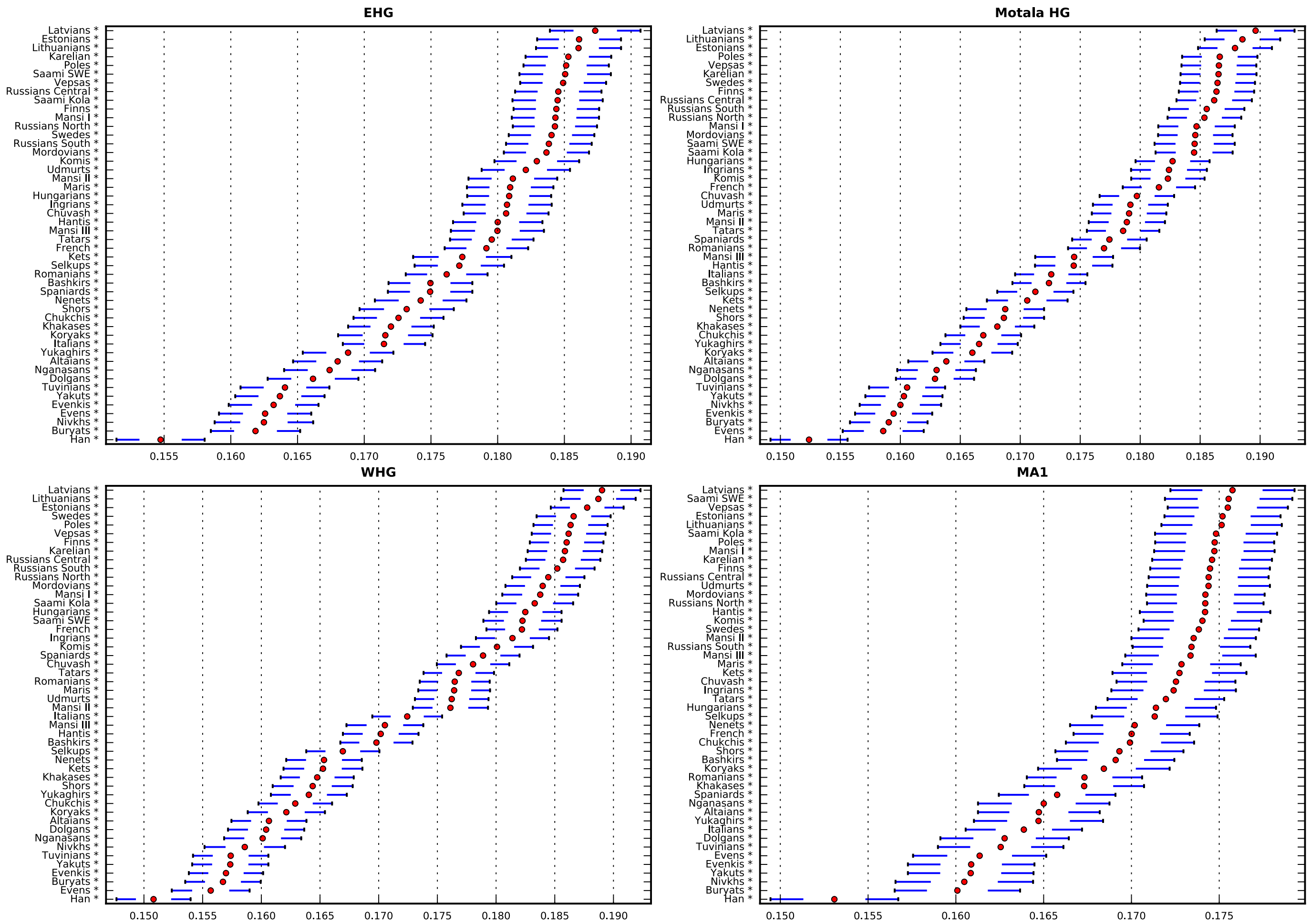
EHG
Latvians *
Estonians *
Lithuanians *
Karelian *
Poles *
Saami SWE *
Vepsas *
Russians Central *
Saami Kola *
Finns *
Mansi I *
Russians North *
Swedes *
Russians South *
Mordovians *
Komis *
Udmurts *
Mansi II *
Maris *
Hungarians *
Ingrians *
Chuvash *
Hantis *
Mansi III *
Tatars *
French *
Kets *
Selkups *
Romanians *
Bashkirs *
Spaniards *
Nenets *
Shors *
Chukchis *
Khakases *
Koryaks *
Italians *
Yukaghirs *
Altaians *
Nganasans *
Dolgans *
Tuvinians *
Yakuts *
Evenkis *
Evens *
Nivkhs *
Buryats *
Han *
0.155
0.160
0.165
0.170
0.175
0.180
0.185
0.190
Motala HG
Latvians *
Lithuanians *
Estonians *
Poles *
Vepsas *
Karelian *
Swedes *
Finns *
Russians Central *
Russians South *
Russians North *
Mansi I *
Mordovians *
Saami SWE *
Saami Kola *
Hungarians *
Ingrians *
Komis *
French *
Chuvash *
Udmurts *
Maris *
Mansi II *
Tatars *
Spaniards *
Romanians *
Mansi III *
Hantis *
Italians *
Bashkirs *
Selkups *
Kets *
Nenets *
Shors *
Khakases *
Chukchis *
Yukaghirs *
Koryaks *
Altaians *
Nganasans *
Dolgans *
Tuvinians *
Yakuts *
Nivkhs *
Evenkis *
Buryats *
Evens *
Han *
0.150
0.155
0.160
0.165
0.170
0.175
0.180
0.185
0.190
WHG
Latvians *
Lithuanians *
Estonians *
Swedes *
Poles *
Vepsas *
Finns *
Karelian *
Russians Central *
Russians South *
Russians North *
Mordovians *
Mansi I *
Saami Kola *
Hungarians *
Saami SWE *
French *
Ingrians *
Komis *
Spaniards *
Chuvash *
Tatars *
Romanians *
Maris *
Udmurts *
Mansi II *
Italians *
Mansi III *
Hantis *
Bashkirs *
Selkups *
Nenets *
Kets *
Khakases *
Shors *
Yukaghirs *
Chukchis *
Koryaks *
Altaians *
Dolgans *
Nganasans *
Nivkhs *
Tuvinians *
Yakuts *
Evenkis *
Buryats *
Evens *
Han *
0.150
0.155
0.160
0.165
0.170
0.175
0.180
0.185
0.190
MA1
Latvians *
Saami SWE *
Vepsas *
Estonians *
Lithuanians *
Saami Kola *
Poles *
Mansi I *
Karelian *
Finns *
Russians Central *
Udmurts *
Mordovians *
Russians North *
Hantis *
Komis *
Swedes *
Mansi II *
Russians South *
Mansi III *
Maris *
Kets *
Chuvash *
Ingrians *
Tatars *
Hungarians *
Selkups *
Nenets *
French *
Chukchis *
Shors *
Bashkirs *
Koryaks *
Romanians *
Khakases *
Spaniards *
Nganasans *
Altaians *
Yukaghirs *
Italians *
Dolgans *
Tuvinians *
Evens *
Evenkis *
Yakuts *
Nivkhs *
Buryats *
Han *
0.150
0.155
0.160
0.165
0.170
0.175

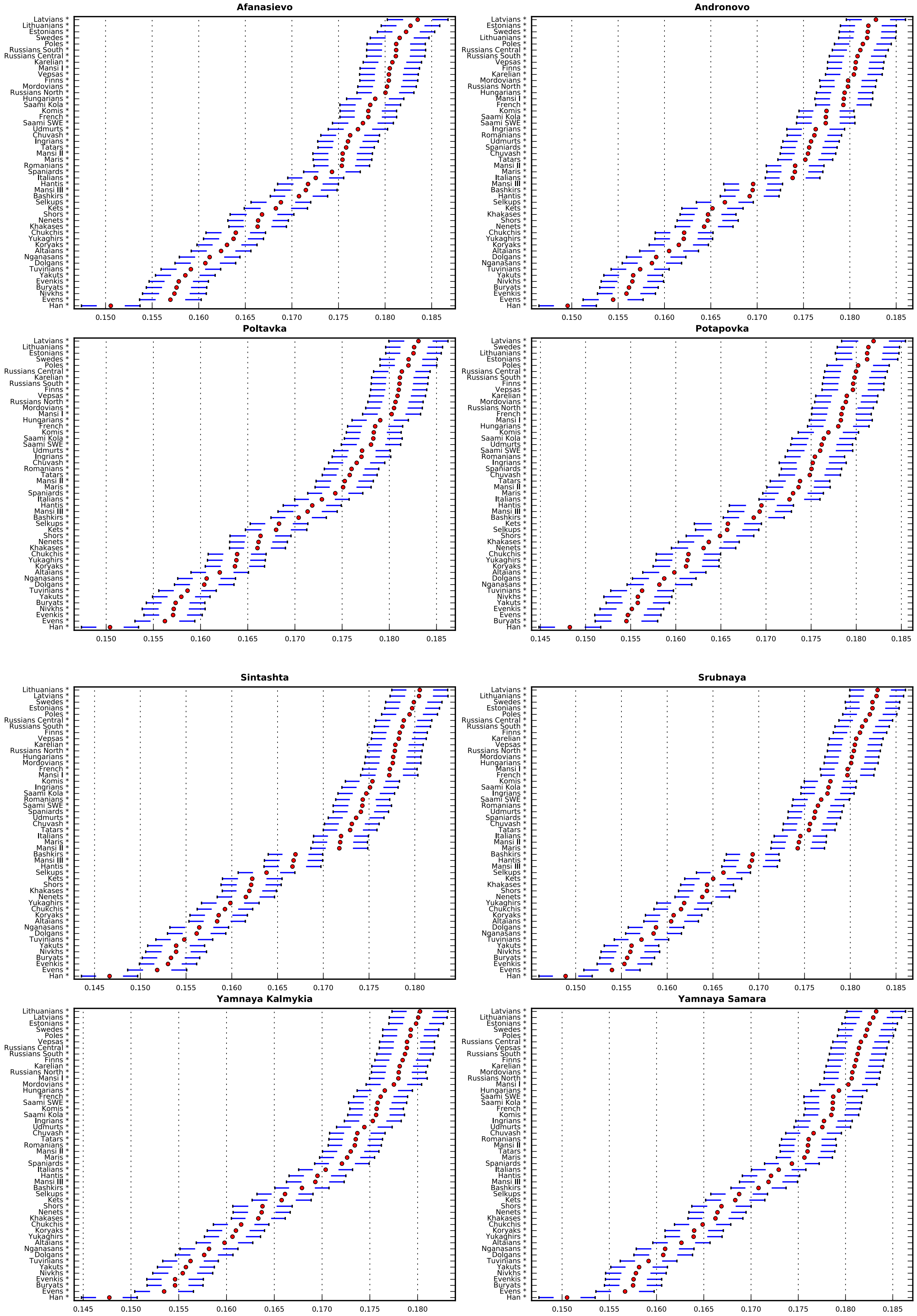
Afanasievo
Andronovo
Poltavka
Potapovka
Sintashta
Srubnaya
Yamnaya Kalmykia
Yamnaya Samara

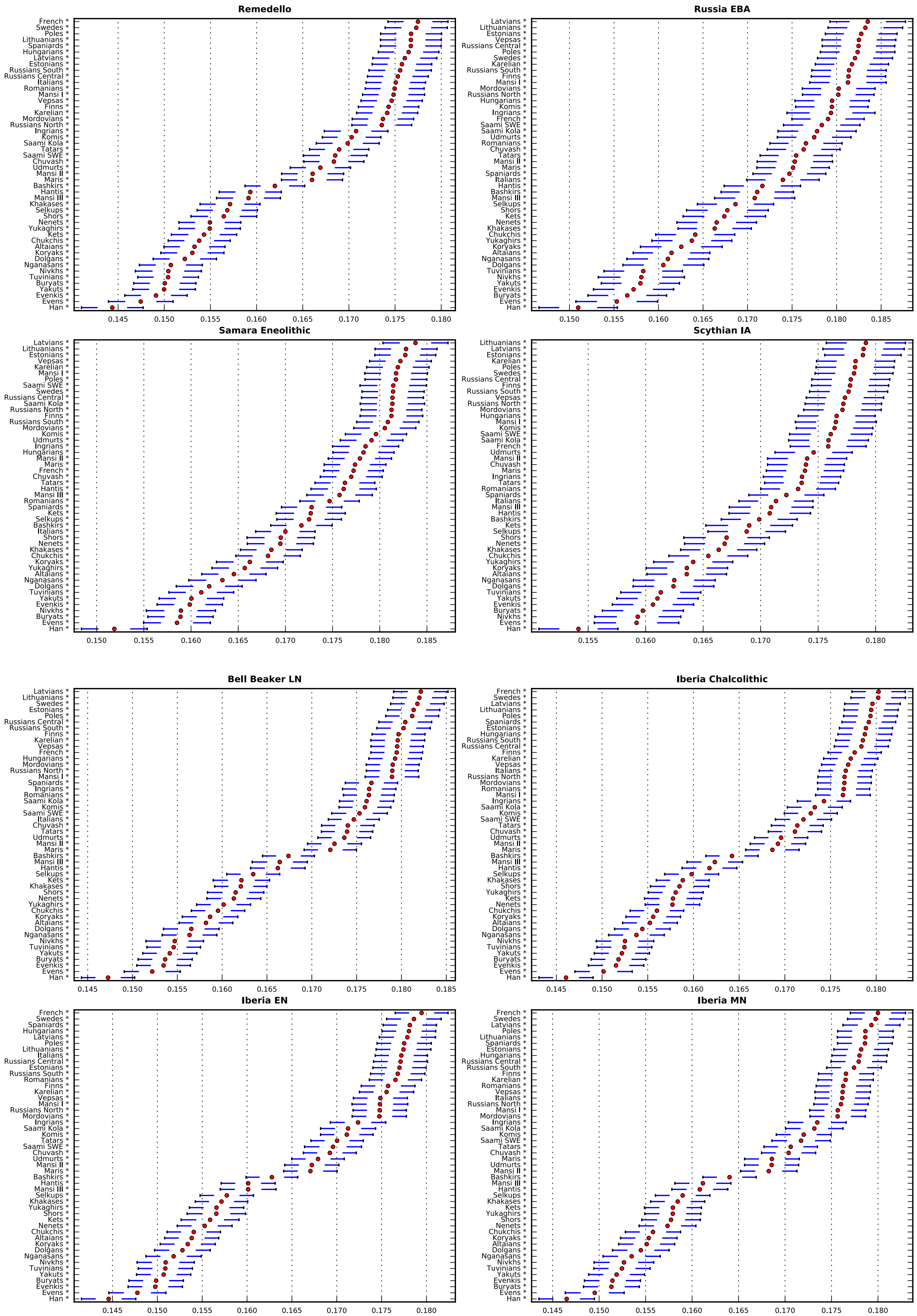
Remedello
French *
Swedes *
Poles *
Lithuanians *
Spaniards *
Hungarians *
Latvians *
Estonians *
Russians South *
Russians Central *
Italians *
Romanians *
Mansi I *
Vepsas *
Finns *
Karelian *
Mordovians *
Russians North *
Ingrians *
Komis *
Saami Kola *
Tatars *
Saami SWE *
Chuvash *
Udmurts *
Mansi II *
Maris *
Bashkirs *
Hantis *
Mansi III *
Khakases *
Selkups *
Shors *
Nenets *
Yukaghirs *
Kets *
Chukchis *
Altaians *
Koryaks *
Dolgans *
Nganasans *
Nivkhs *
Tuvinians *
Buryats *
Yakuts *
Evenkis *
Evens *
Han *
0.145
0.150
0.155
0.160
0.165
0.170
0.175
0.180
Russia EBA
Latvians *
Lithuanians *
Estonians *
Vepsas *
Russians Central *
Poles *
Swedes *
Karelian *
Russians South *
Finns *
Mansi I *
Mordovians *
Russians North *
Hungarians *
Komis *
Ingrians *
French *
Saami SWE *
Saami Kola *
Udmurts *
Romanians *
Chuvash *
Tatars *
Mansi II *
Maris *
Spaniards *
Italians *
Hantis *
Bashkirs *
Mansi III *
Selkups *
Shors *
Kets *
Nenets *
Khakases *
Chukchis *
Yukaghirs *
Koryaks *
Altaians *
Nganasans *
Dolgans *
Tuvinians *
Nivkhs *
Yakuts *
Evenkis *
Buryats *
Evens *
Han *
0.150
0.155
0.160
0.165
0.170
0.175
0.180
0.185
Samara Eneolithic
Latvians *
Lithuanians *
Estonians *
Vepsas *
Karelian *
Mansi I *
Poles *
Saami SWE *
Swedes *
Russians Central *
Saami Kola *
Russians North *
Finns *
Russians South *
Mordovians *
Komis *
Udmurts *
Ingrians *
Hungarians *
Mansi II *
Maris *
French *
Chuvash *
Tatars *
Hantis *
Mansi III *
Romanians *
Spaniards *
Kets *
Selkups *
Bashkirs *
Italians *
Shors *
Nenets *
Khakases *
Chukchis *
Koryaks *
Yukaghirs *
Altaians *
Nganasans *
Dolgans *
Tuvinians *
Yakuts *
Evenkis *
Nivkhs *
Buryats *
Evens *
Han *
0.150
0.155
0.160
0.165
0.170
0.175
0.180
0.185
Scythian IA
Lithuanians *
Latvians *
Estonians *
Karelian *
Poles *
Swedes *
Russians Central *
Finns *
Russians South *
Vepsas *
Russians North *
Mordovians *
Hungarians *
Mansi I *
Komis *
Saami SWE *
Saami Kola *
French *
Udmurts *
Mansi II *
Chuvash *
Maris *
Ingrians *
Tatars *
Romanians *
Spaniards *
Italians *
Mansi III *
Hantis *
Bashkirs *
Kets *
Selkups *
Shors *
Nenets *
Khakases *
Chukchis *
Yukaghirs *
Koryaks *
Altaians *
Nganasans *
Dolgans *
Tuvinians *
Yakuts *
Evenkis *
Buryats *
Nivkhs *
Evens *
Han *
0.155
0.160
0.165
0.170
0.175
0.180
Bell Beaker LN
Latvians *
Lithuanians *
Swedes *
Estonians *
Poles *
Russians Central *
Russians South *
Finns *
Karelian *
Vepsas *
French *
Hungarians *
Mordovians *
Russians North *
Mansi I *
Spaniards *
Ingrians *
Romanians *
Saami Kola *
Komis *
Saami SWE *
Italians *
Chuvash *
Tatars *
Udmurts *
Mansi II *
Maris *
Bashkirs *
Mansi III *
Hantis *
Selkups *
Kets *
Khakases *
Shors *
Nenets *
Yukaghirs *
Chukchis *
Koryaks *
Altaians *
Dolgans *
Nganasans *
Nivkhs *
Tuvinians *
Yakuts *
Buryats *
Evenkis *
Evens *
Han *
0.145
0.150
0.155
0.160
0.165
0.170
0.175
0.180
0.185
Iberia Chalcolithic
French *
Swedes *
Latvians *
Lithuanians *
Poles *
Spaniards *
Estonians *
Hungarians *
Russians South *
Russians Central *
Finns *
Karelian *
Vepsas *
Italians *
Russians North *
Mordovians *
Romanians *
Mansi I *
Ingrians *
Saami Kola *
Komis *
Saami SWE *
Tatars *
Chuvash *
Udmurts *
Mansi II *
Maris *
Bashkirs *
Mansi III *
Hantis *
Selkups *
Khakases *
Shors *
Yukaghirs *
Kets *
Nenets *
Chukchis *
Koryaks *
Altaians *
Dolgans *
Nganasans *
Nivkhs *
Tuvinians *
Yakuts *
Buryats *
Evenkis *
Evens *
Han *
0.145
0.150
0.155
0.160
0.165
0.170
0.175
0.180
Iberia EN
French *
Swedes *
Spaniards *
Hungarians *
Latvians *
Poles *
Lithuanians *
Italians *
Russians Central *
Estonians *
Russians South *
Romanians *
Finns *
Karelian *
Vepsas *
Mansi I *
Russians North *
Mordovians *
Ingrians *
Saami Kola *
Komis *
Tatars *
Saami SWE *
Chuvash *
Udmurts *
Mansi II *
Maris *
Bashkirs *
Hantis *
Mansi III *
Selkups *
Khakases *
Yukaghirs *
Shors *
Kets *
Nenets *
Chukchis *
Altaians *
Koryaks *
Dolgans *
Nganasans *
Nivkhs *
Tuvinians *
Yakuts *
Buryats *
Evenkis *
Evens *
Han *
0.145
0.150
0.155
0.160
0.165
0.170
0.175
0.180
Iberia MN
French *
Swedes *
Latvians *
Poles *
Lithuanians *
Spaniards *
Estonians *
Hungarians *
Russians Central *
Russians South *
Finns *
Karelian *
Romanians *
Vepsas *
Italians *
Russians North *
Mansi I *
Mordovians *
Ingrians *
Saami Kola *
Komis *
Saami SWE *
Tatars *
Chuvash *
Maris *
Udmurts *
Mansi II *
Bashkirs *
Mansi III *
Hantis *
Selkups *
Khakases *
Kets *
Yukaghirs *
Shors *
Nenets *
Chukchis *
Koryaks *
Altaians *
Dolgans *
Nganasans *
Nivkhs *
Tuvinians *
Yakuts *
Evenkis *
Buryats *
Evens *
Han *
0.145
0.150
0.155
0.160
0.165
0.170
0.175
0.180

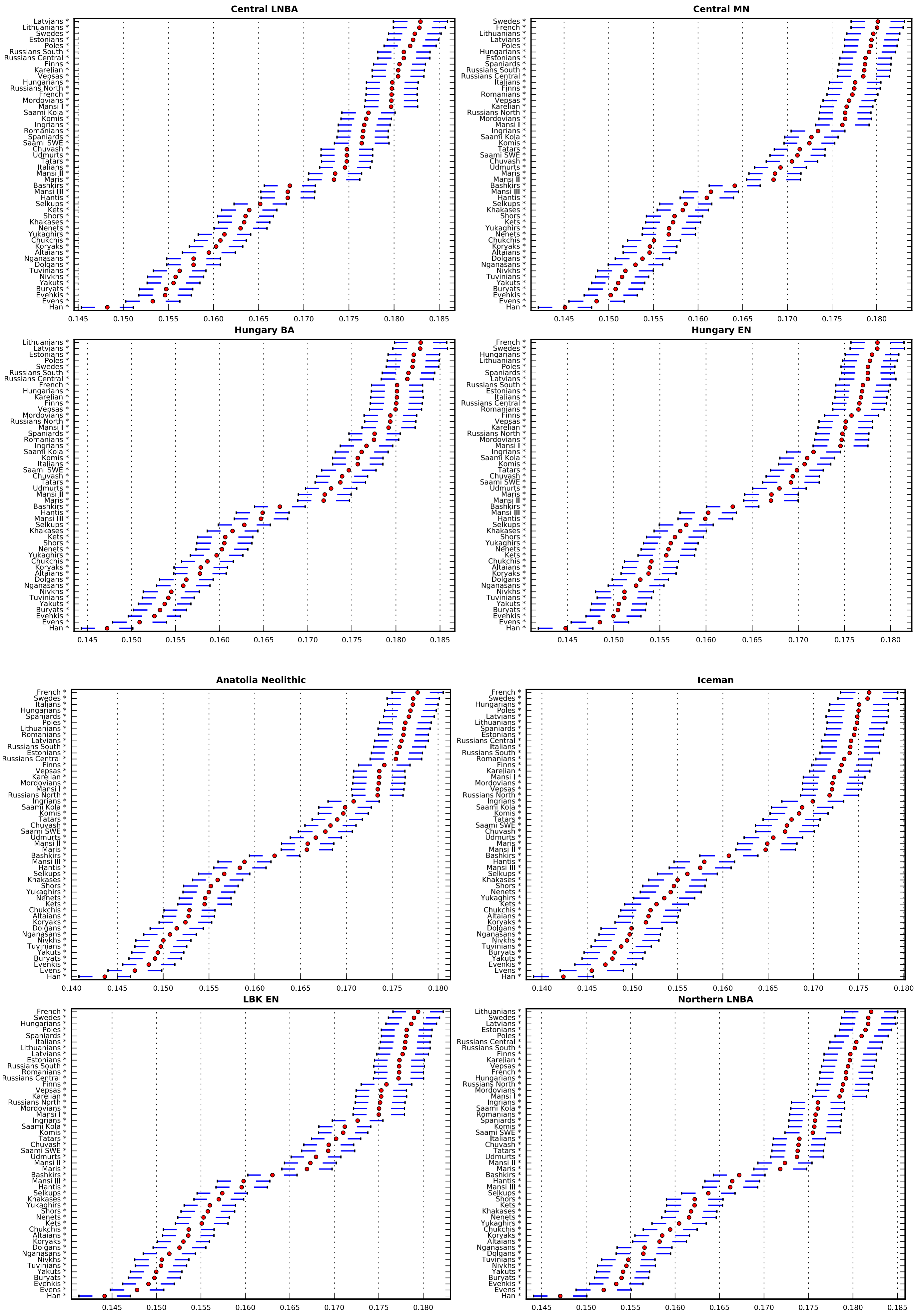
Central LNBA
Central MN
Hungary BA
Hungary EN
Anatolia Neolithic
Iceman
LBK EN
Northern LNBA

Figure S8. Results of outgroup *f3* test between modern samples and ancient genomes. The (modern reference, ancient reference; outgroup) test configuration was used, and Yoruba from Africa (YRI) was used and an outgroup. Each individual plot shows tests results between a reference given in the title of that plot, and all other reference populations (y-axis). For convenience, results for plotting were grouped using modern reference population (**A.**) or ancient reference cluster or genome (**B.**). Detailed results, including the number of SNPs used for each particular test are given in the Additional file 14: Table S13.

A.

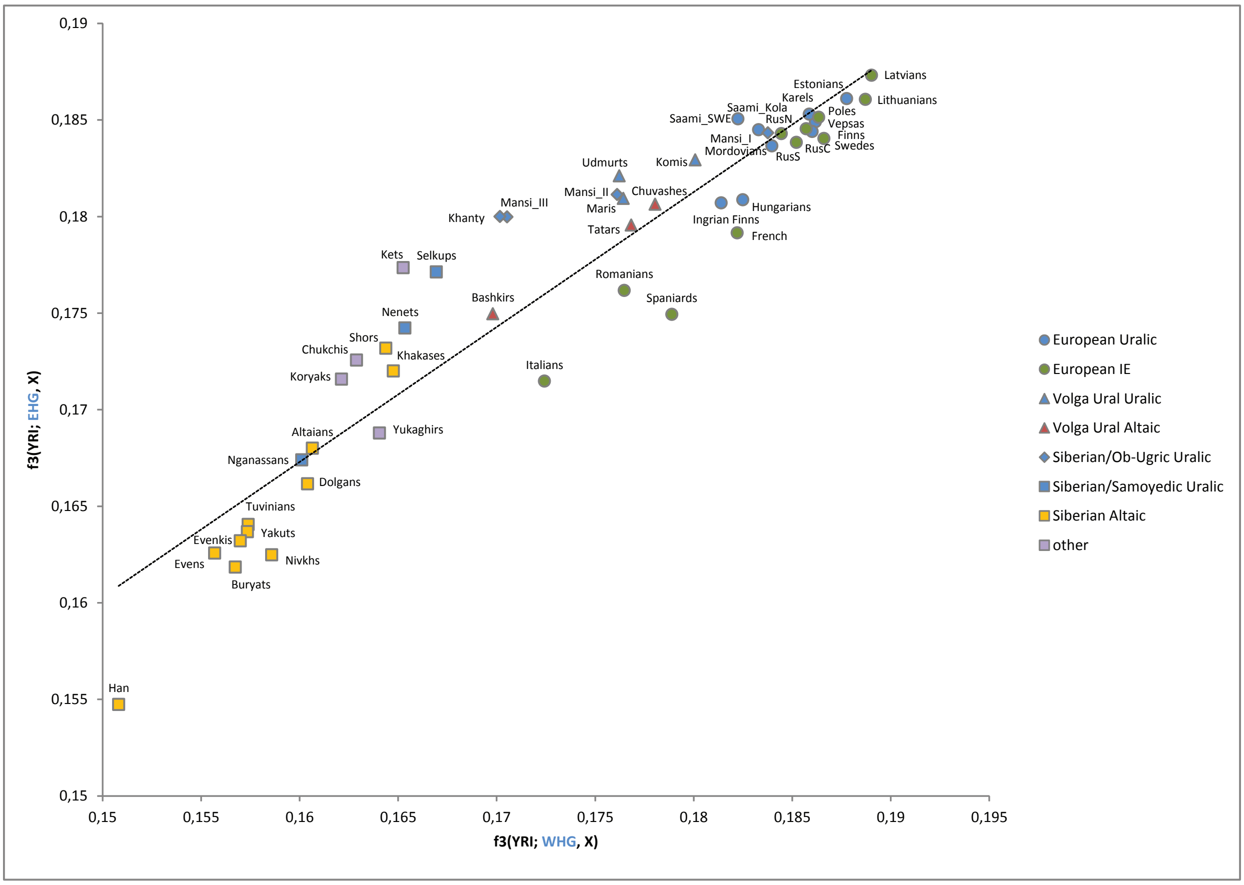

0,19
0,185
0,18
0,175
0,17
0,165
0,16
0,155
0,15
f3(YRI; EHG, X)
0,15
0,155
0,16
0,165
0,17
0,175
0,18
0,185
0,19
0,195
f3(YRI; WHG, X)
Latvians
Estonians
Karels
Lithuanians
Saami_Kola
Poles
Saami_SWE
RusN
Vepsas
Finns
Mansi_I
Swedes
Mordovians
RusC
RusS
Udmurts
Komis
Mansi_II
Chuvashes
Mansi_III
Maris
Hungarians
Khanty
Ingrian Finns
Tatars
French
Kets
Selkups
Romanians
Bashkirs
Spaniards
Nenets
Shors
Chukchis
Khakases
Italians
Koryaks
Yukaghirs
Altaians
Nganassans
Dolgans
Tuvinians
Yakuts
Evenkis
Evens
Nivkhs
Buryats
Han
European Uralic
European IE
Volga Ural Uralic
Volga Ural Altaic
Siberian/Ob-Ugric Uralic
Siberian/Samoyedic Uralic
Siberian Altaic
other

B.

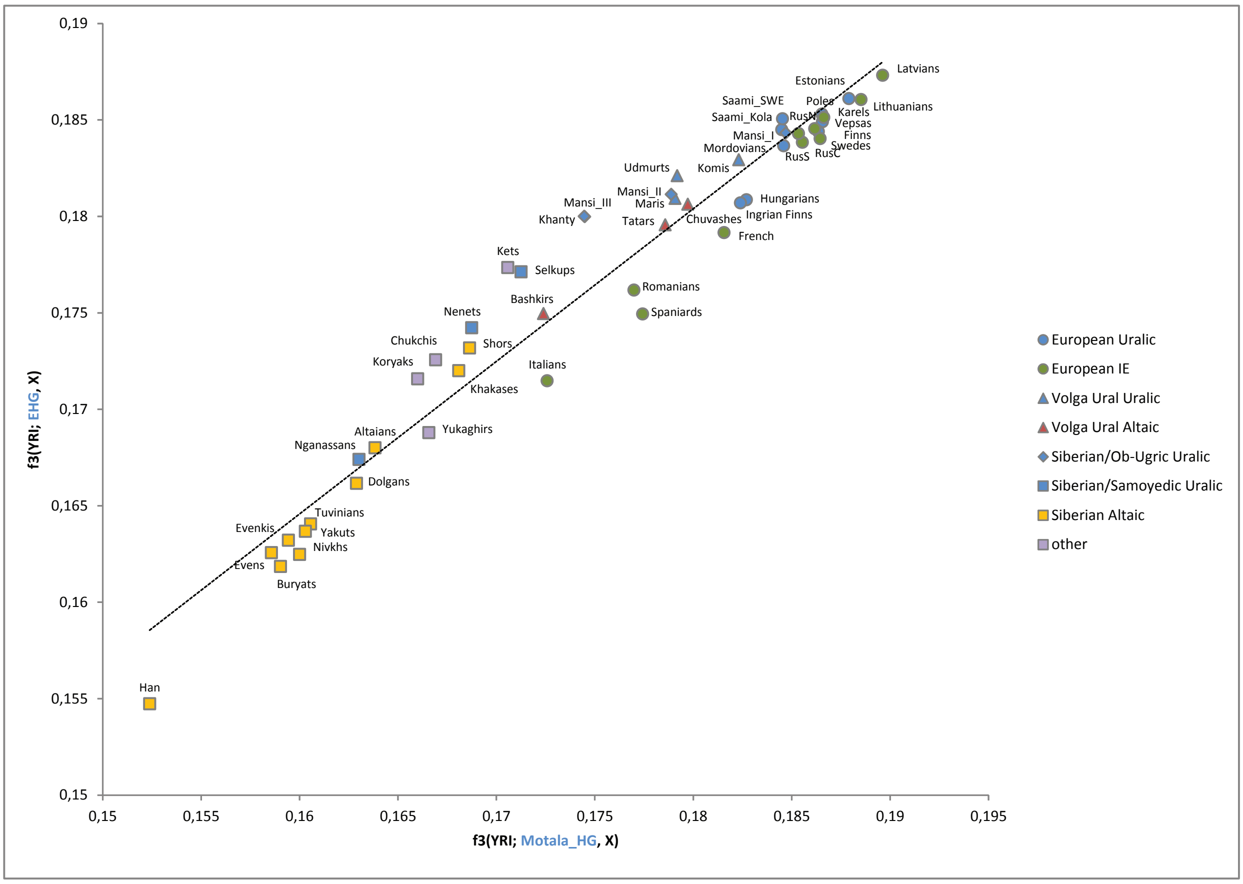

0,19
0,185
0,18
0,175
0,17
0,165
0,16
0,155
0,15
f3(YRI; EHG, X)
0,15
0,155
0,16
0,165
0,17
0,175
0,18
0,185
0,19
0,195
f3(YRI; Motala_HG, X)
Latvians
Estonians
Saami_SWE
Poles
Lithuanians
Saami_Kola
RusN
Karels
Vepsas
Mansi_I
Finns
Mordovians
Swedes
RusS
RusC
Udmurts
Komis
Mansi_II
Hungarians
Mansi_III
Maris
Khanty
Tatars
Chuvashes
Ingrian Finns
French
Kets
Selkups
Romanians
Bashkirs
Spaniards
Nenets
Chukchis
Shors
Koryaks
Italians
Khakases
Altaians
Yukaghirs
Nganassans
Dolgans
Tuvinians
Evenkis
Yakuts
Nivkhs
Evens
Buryats
Han
European Uralic
European IE
Volga Ural Uralic
Volga Ural Altaic
Siberian/Ob-Ugric Uralic
Siberian/Samoyedic Uralic
Siberian Altaic
other

C.

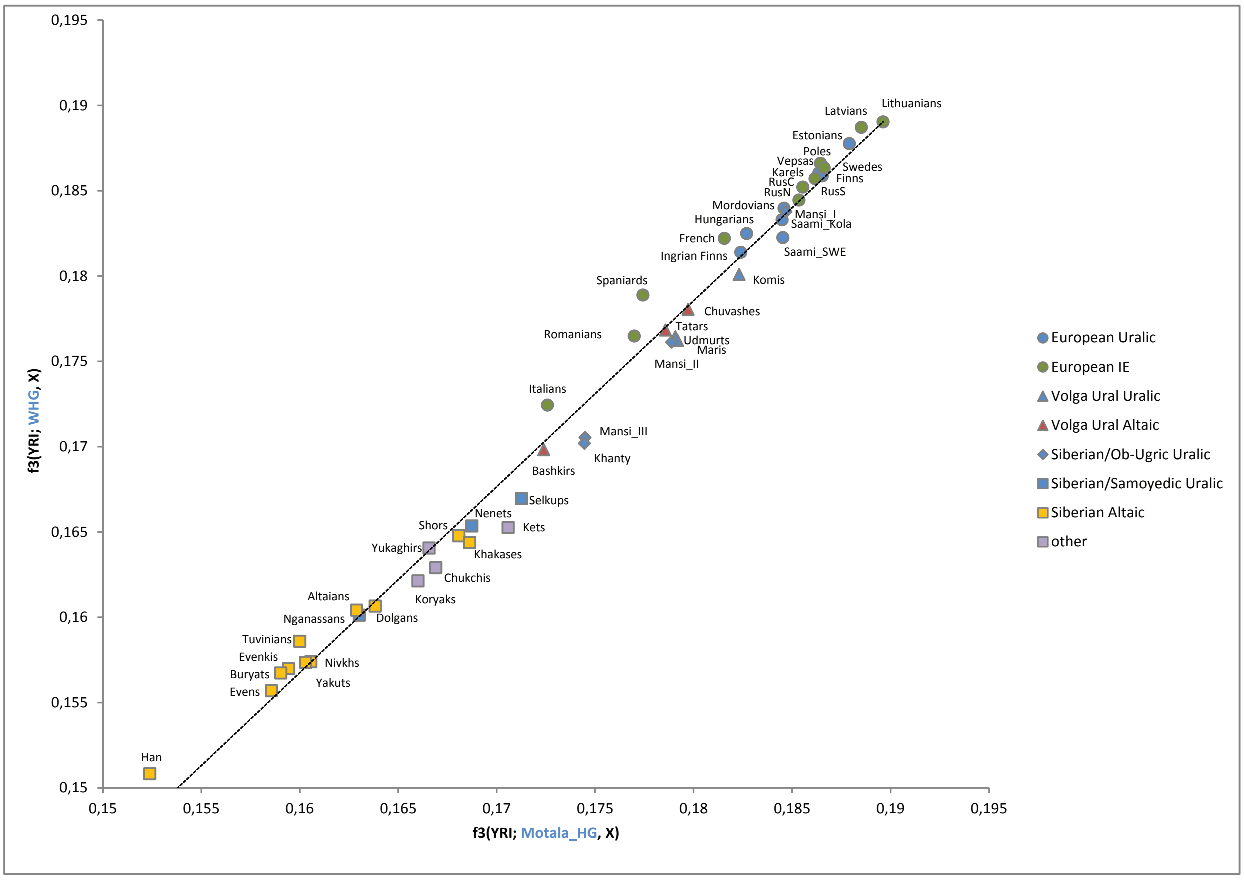

0,195
0,19
0,185
0,18
0,175
0,17
0,165
0,16
0,155
0,15
f3(YRI; WHG, X)
0,15
0,155
0,16
0,165
0,17
0,175
0,18
0,185
0,19
0,195
f3(YRI; Motala_HG, X)
Lithuanians
Latvians
Estonians
Poles
Vepsas
Swedes
Karels
Finns
RusC
RusN
RusS
Mordovians
Mansi_I
Hungarians
Saami_Kola
French
Ingrian Finns
Saami_SWE
Komis
Spaniards
Chuvashes
Tatars
Romanians
Udmurts
Maris
Mansi_II
Italians
Mansi_III
Khanty
Bashkirs
Selkups
Nenets
Shors
Kets
Yukaghirs
Khakases
Chukchis
Koryaks
Altaians
Nganassans
Dolgans
Tuvinians
Evenkis
Nivkhs
Buryats
Yakuts
Evens
Han
European Uralic
European IE
Volga Ural Uralic
Volga Ural Altaic
Siberian/Ob-Ugric Uralic
Siberian/Samoyedic Uralic
Siberian Altaic
other

D.

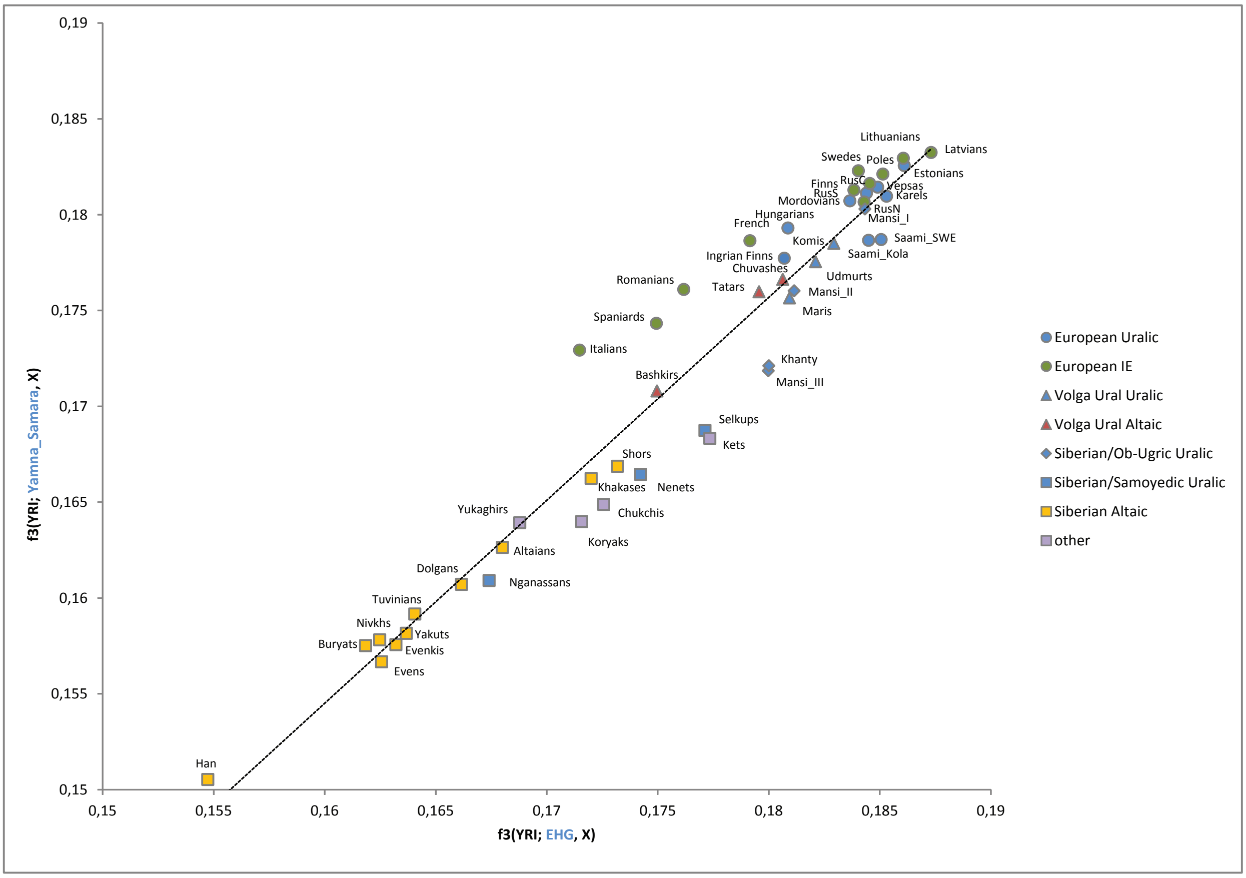

0,19
0,185
0,18
0,175
0,17
0,165
0,16
0,155
0,15
f3(YRI; Yamna_Samara, X)
0,15
0,155
0,16
0,165
0,17
0,175
0,18
0,185
0,19
f3(YRI; EHG, X)
Lithuanians
Latvians
Swedes
Poles
Estonians
Finns
RusC
Vepsas
RusS
Karels
Mordovians
RusN
Hungarians
Mansi_I
French
Komis
Saami_SWE
Ingrian Finns
Saami_Kola
Chuvashes
Udmurts
Romanians
Tatars
Mansi_II
Maris
Spaniards
Italians
Khanty
Bashkirs
Mansi_III
Selkups
Kets
Shors
Khakases
Nenets
Chukchis
Yukaghirs
Koryaks
Altaians
Dolgans
Nganassans
Tuvinians
Nivkhs
Yakuts
Buryats
Evenkis
Evens
Han
European Uralic
European IE
Volga Ural Uralic
Volga Ural Altaic
Siberian/Ob-Ugric Uralic
Siberian/Samoyedic Uralic
Siberian Altaic
other

E.

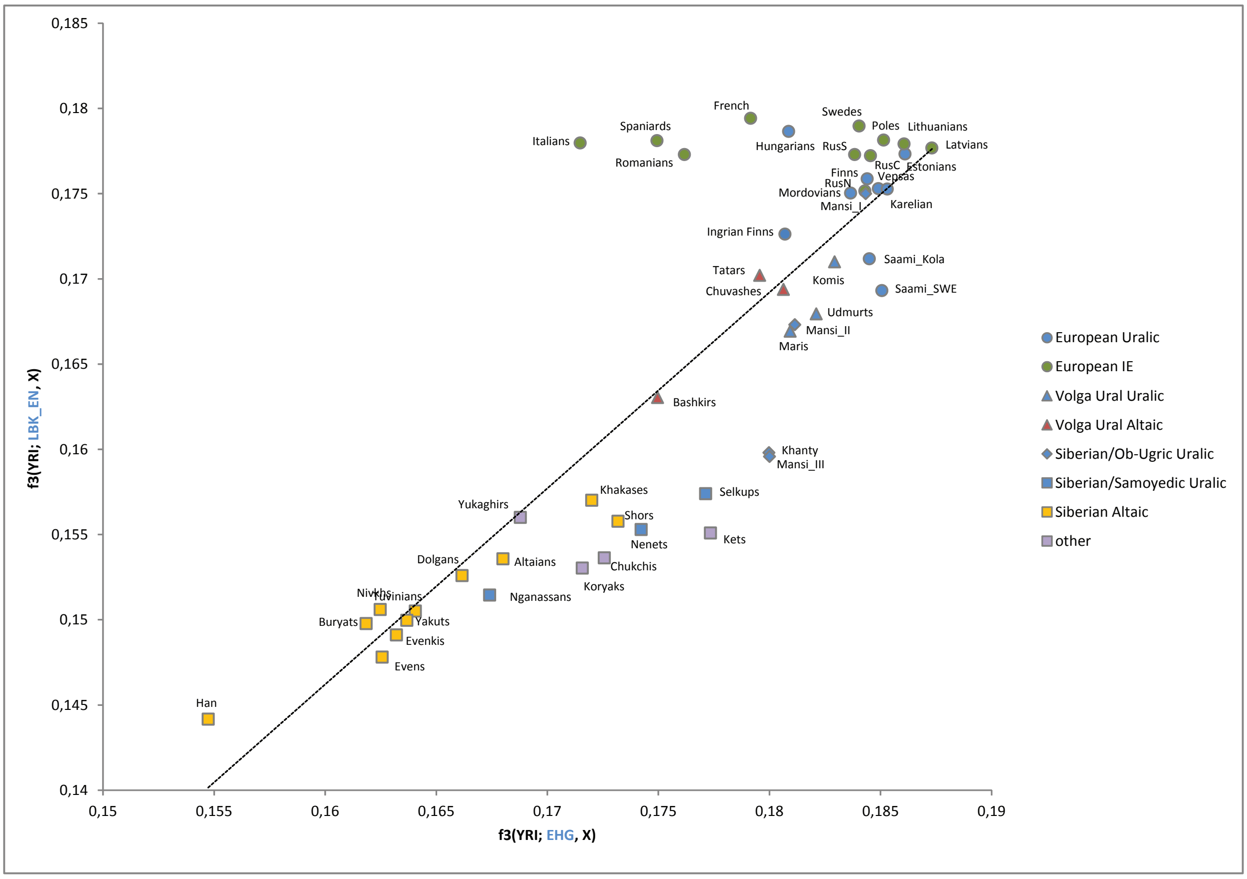

f3(YRI; LBK_EN, X)
f3(YRI; EHG, X)
European Uralic
European IE
Volga Ural Uralic
Volga Ural Altaic
Siberian/Ob-Ugric Uralic
Siberian/Samoyedic Uralic
Siberian Altaic
other

F.

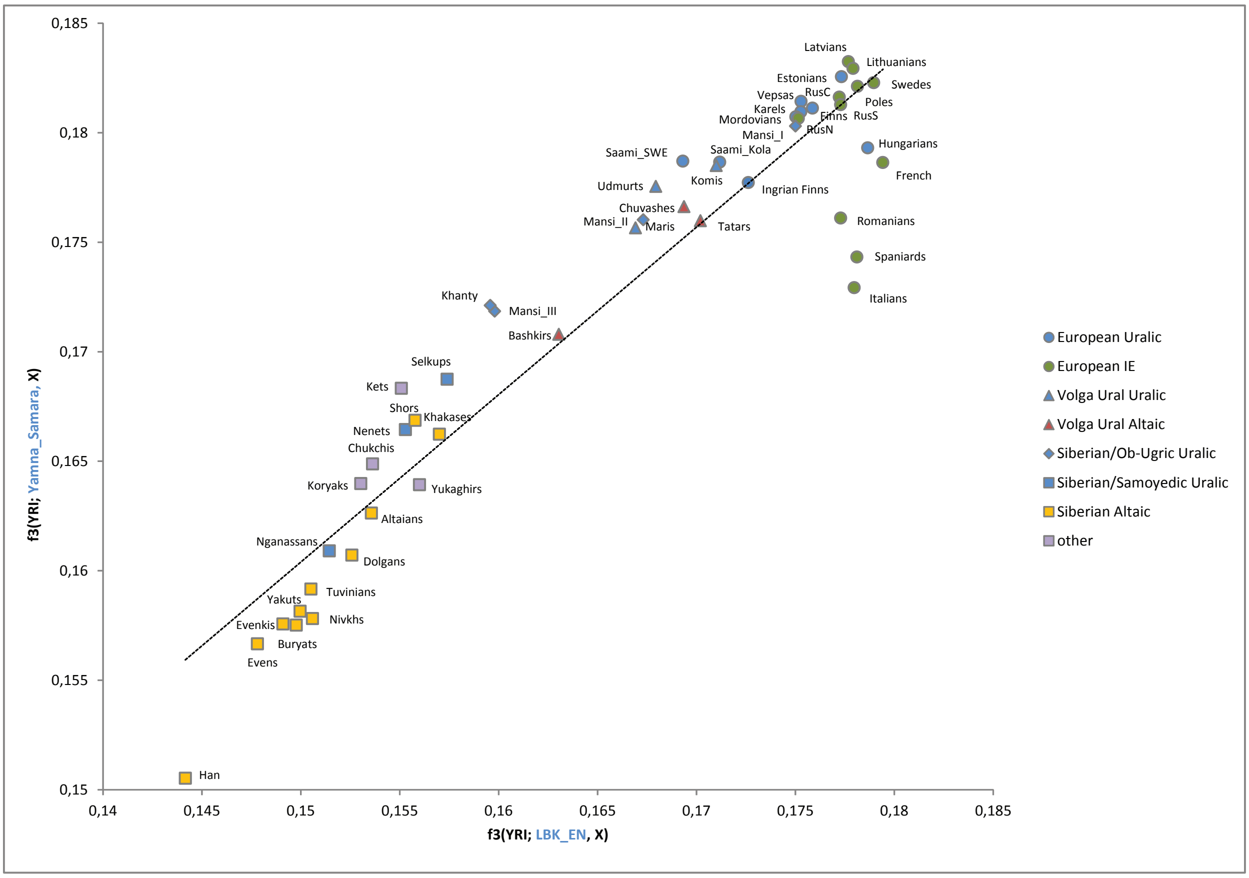

f3(YRI; Yamna_Samara, X)
f3(YRI; LBK_EN, X)
European Uralic
European IE
Volga Ural Uralic
Volga Ural Altaic
Siberian/Ob-Ugric Uralic
Siberian/Samoyedic Uralic
Siberian Altaic
other

Figure S9. Outgroup f3-statistics' results in the form f3(Yorubas; ancient Eurasian population, modern Eurasian population) plotted pairwise against each other. The information about the modern and ancient individuals is listed in Additional file 1: Table S1, underlying data of f3 statistics is from Additional file 14: Table S13. F3 of Eastern hunter gatherer (EHG) is plotted against f3 of **A.** western hunter gatherer (WHG), **B.** Scandinavian hunter gatherer (Motala_HG), **D.** Bronze Age steppe people (Yamna_Samara) and **E.** Central European early farmers (LBK_EN). **C.** F3 of WHG is plotted against f3 of Motala_HG and **F.** f3 of Yamna_Samara against f3 of LBK_EN.

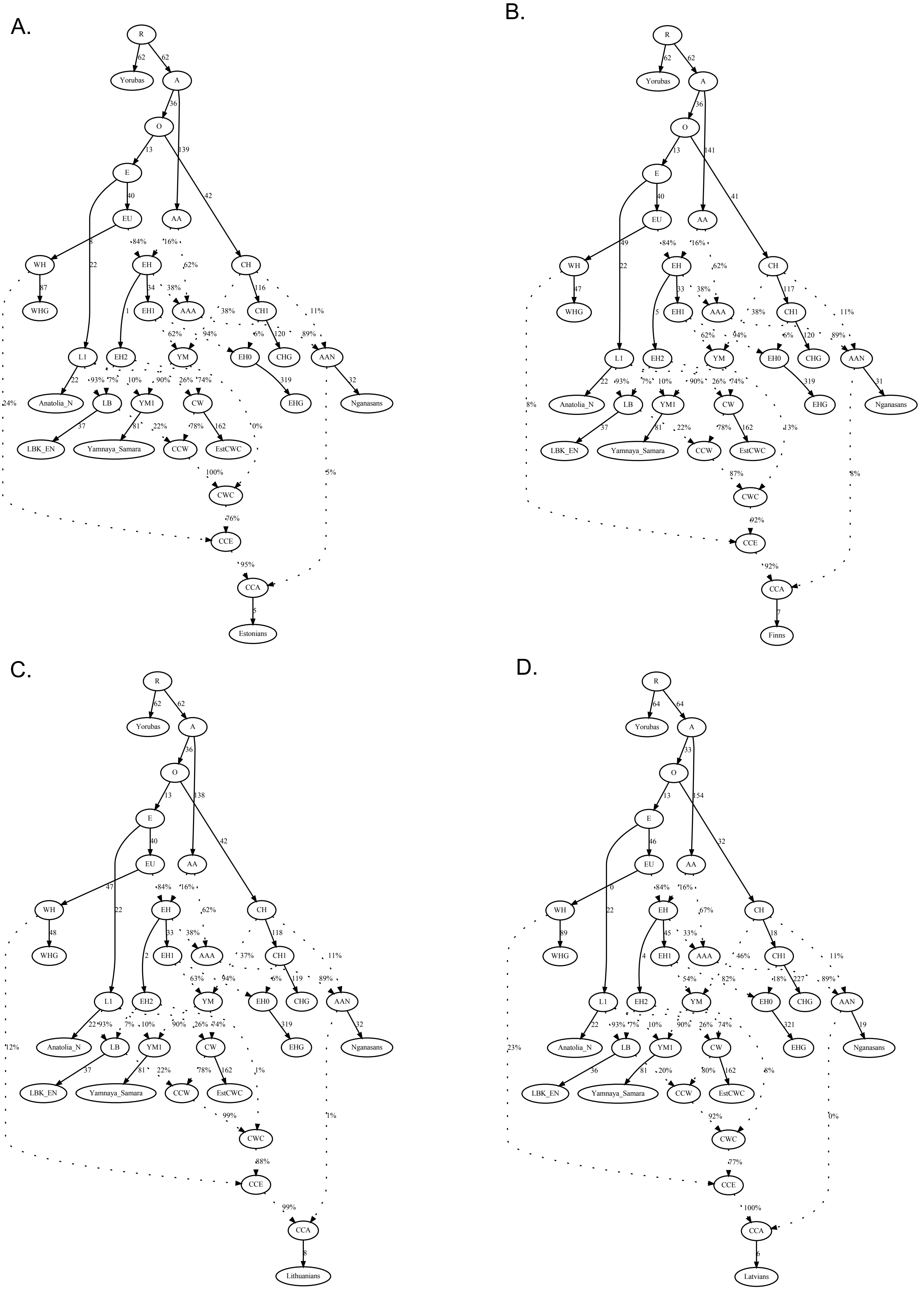
A.
R
Yorubas
A
O
E
EU
AA
CH
WH
EH
WHG
EH1
AAA
CH1
L1
EH2
YM
EH0
CHG
AAN
Anatolia_N
LB
YM1
CW
EHG
Nganasans
LBK_EN
Yamnaya_Samara
CCW
EstCWC
CWC
CCE
CCA
Estonians
B.
Finns
C.
Lithuanians
D.
Latvians

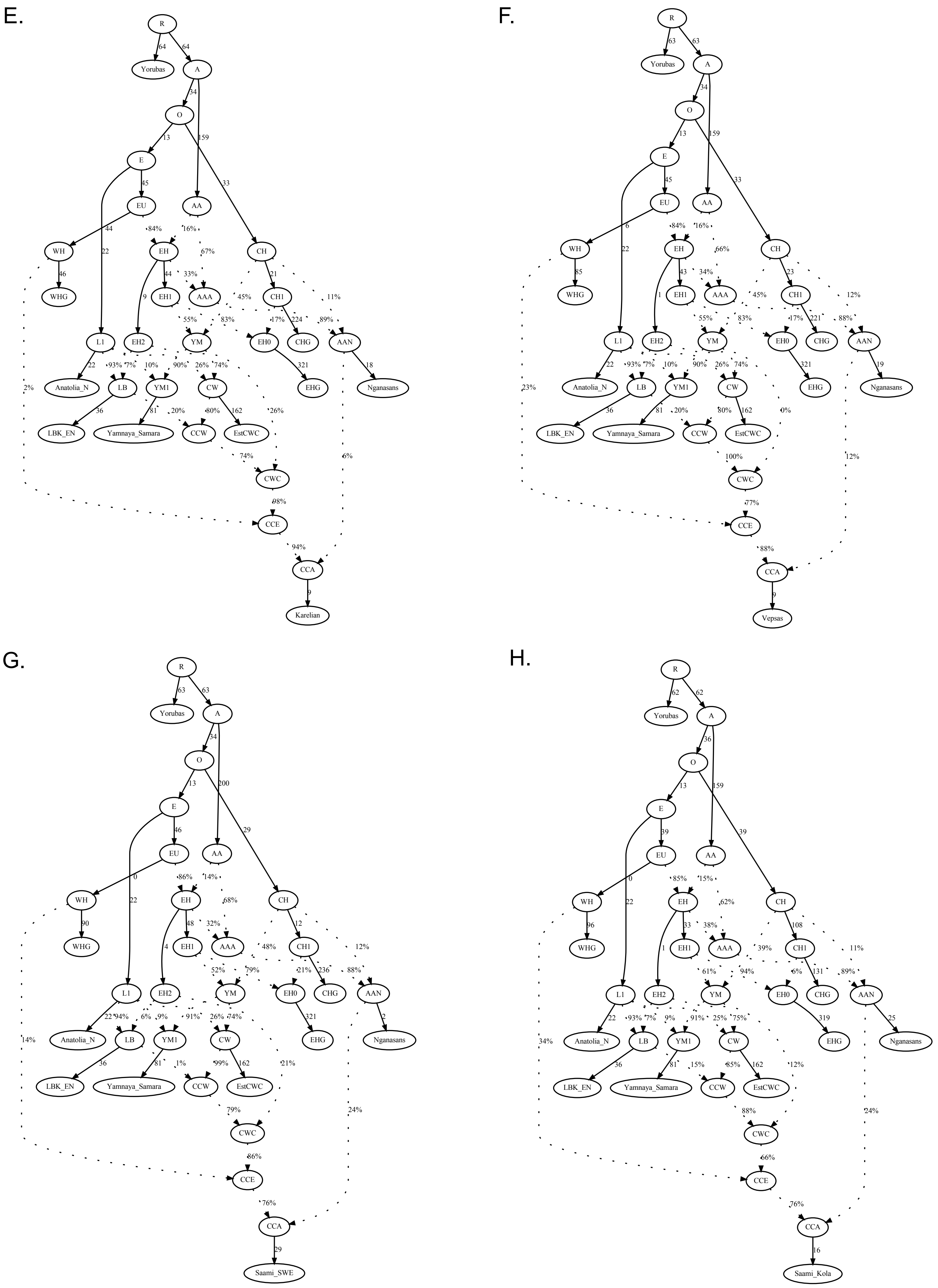
E.
R
Yorubas
A
O
E
EU
AA
CH
WH
EH
WHG
EH1
AAA
CH1
L1
EH2
YM
EH0
CHG
AAN
Anatolia_N
LB
YM1
CW
EHG
Nganasans
LBK_EN
Yamnaya_Samara
CCW
EstCWC
CWC
CCE
CCA
Karelian
F.
Vepsas
G.
Saami_SWE
H.
Saami_Kola

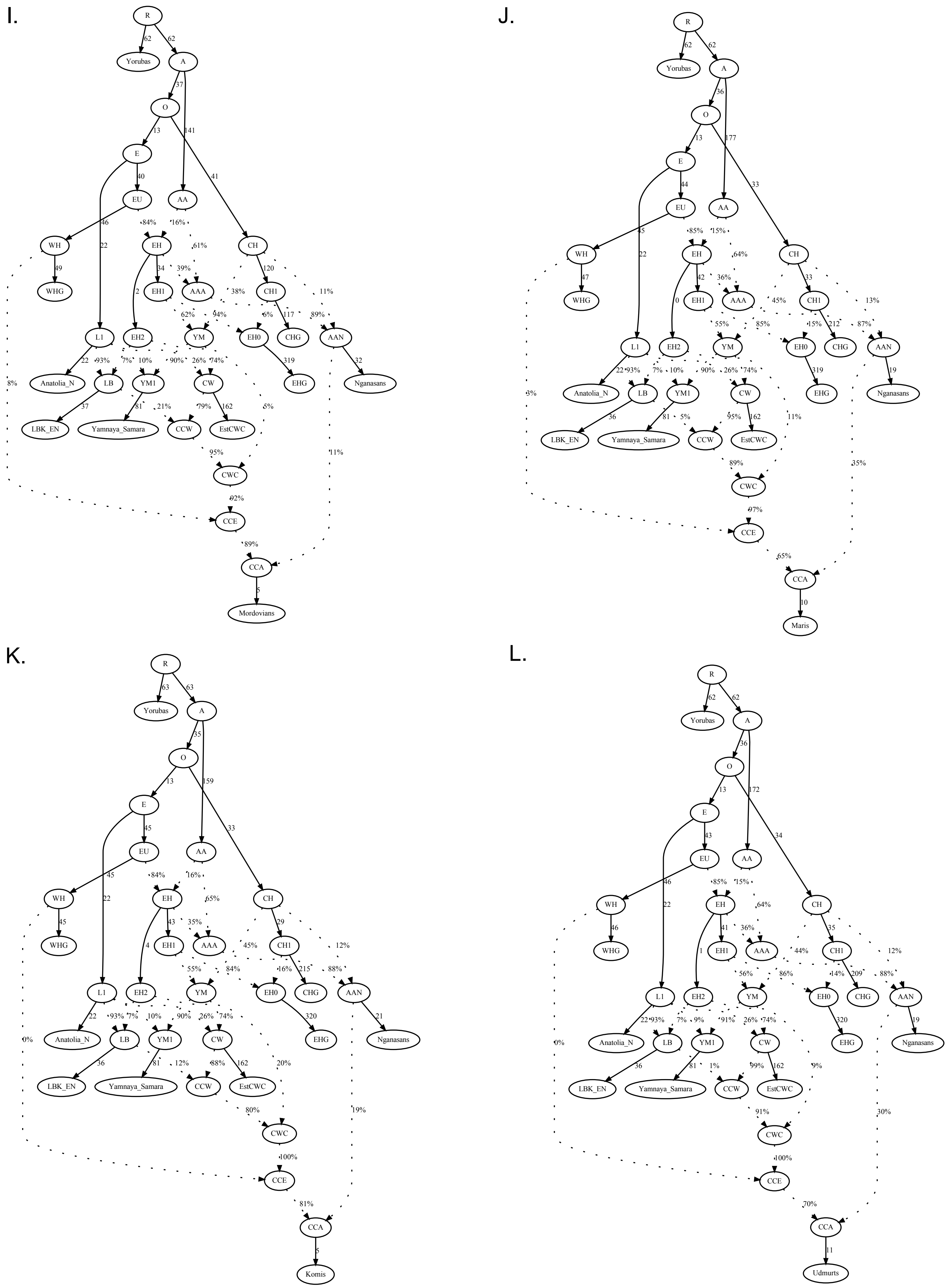
I.
R
62
62
Yorubas
A
37
141
O
13
41
E
40
EU
AA
46
22
84%
16%
61%
WH
EH
CH
49
34
39%
120
WHG
EH1
AAA
38%
CH1
11%
2
62%
94%
6%
117
89%
L1
EH2
YM
EH0
CHG
AAN
22
93%
7%
10%
90%
26%
74%
319
32
8%
Anatolia_N
LB
YM1
CW
EHG
Nganasans
37
81
21%
79%
162
5%
LBK_EN
Yamnaya_Samara
CCW
EstCWC
95%
11%
CWC
92%
CCE
89%
CCA
5
Mordovians
J.
R
62
62
Yorubas
A
36
177
O
13
33
E
44
EU
AA
45
22
85%
15%
64%
WH
EH
CH
47
42
36%
33
WHG
EH1
AAA
45%
CH1
13%
0
55%
85%
15%
212
87%
L1
EH2
YM
EH0
CHG
AAN
22
93%
7%
10%
90%
26%
74%
319
19
3%
Anatolia_N
LB
YM1
CW
EHG
Nganasans
36
81
5%
95%
162
11%
LBK_EN
Yamnaya_Samara
CCW
EstCWC
89%
35%
CWC
97%
CCE
65%
CCA
10
Maris
K.
R
63
63
Yorubas
A
35
159
O
13
33
E
45
EU
AA
45
22
84%
16%
65%
WH
EH
CH
45
43
35%
29
WHG
EH1
AAA
45%
CH1
12%
4
55%
84%
16%
215
88%
L1
EH2
YM
EH0
CHG
AAN
22
93%
7%
10%
90%
26%
74%
320
21
0%
Anatolia_N
LB
YM1
CW
EHG
Nganasans
36
81
12%
88%
162
20%
LBK_EN
Yamnaya_Samara
CCW
EstCWC
80%
19%
CWC
100%
CCE
81%
CCA
5
Komis
L.
R
62
62
Yorubas
A
36
172
O
13
34
E
43
EU
AA
46
22
85%
15%
64%
WH
EH
CH
46
41
36%
35
WHG
EH1
AAA
44%
CH1
12%
1
56%
86%
14%
209
88%
L1
EH2
YM
EH0
CHG
AAN
22
93%
7%
9%
91%
26%
74%
320
19
0%
Anatolia_N
LB
YM1
CW
EHG
Nganasans
36
81
1%
99%
162
9%
LBK_EN
Yamnaya_Samara
CCW
EstCWC
91%
30%
CWC
100%
CCE
70%
CCA
11
Udmurts

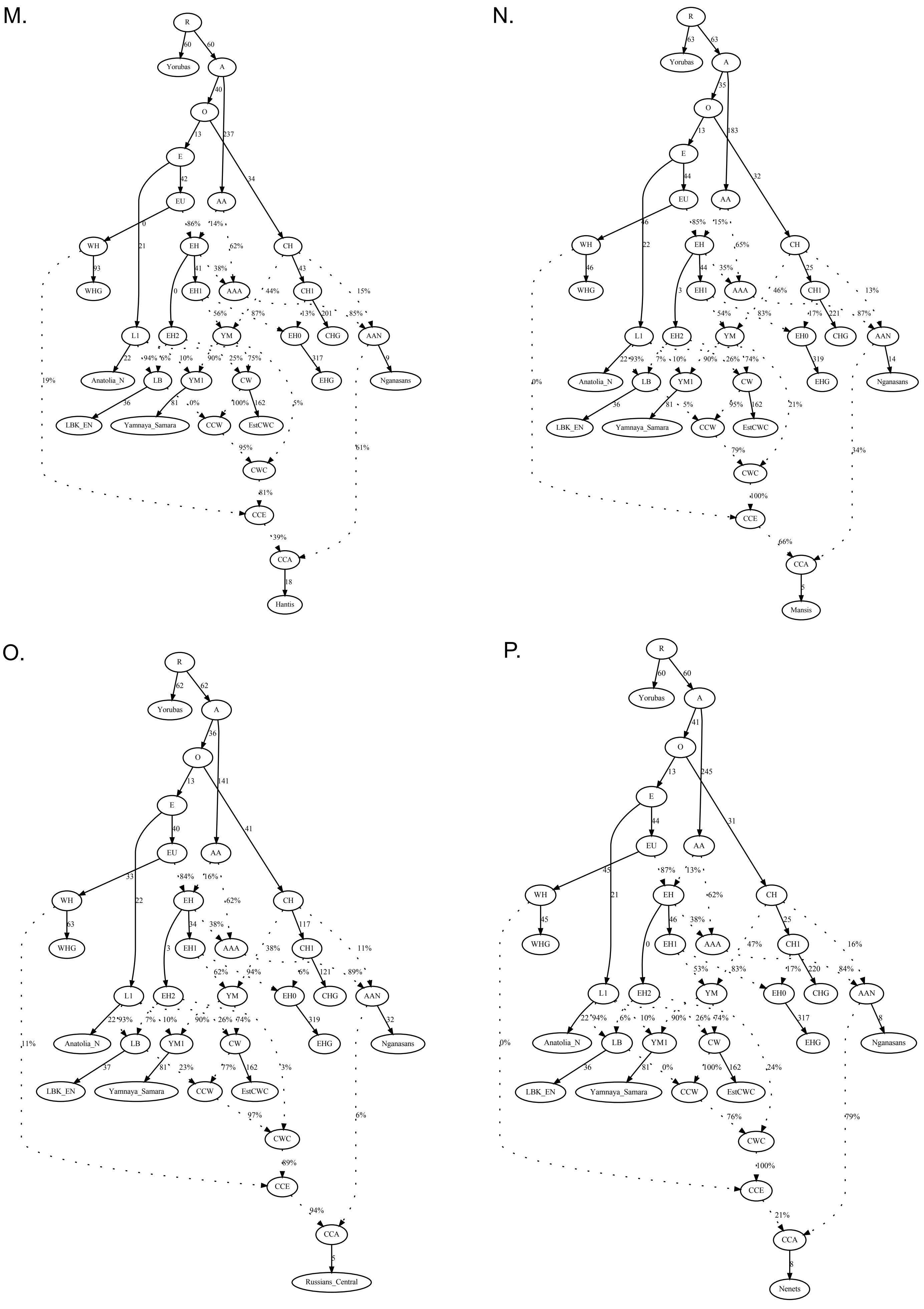
M.
R
Yorubas
A
O
E
EU
AA
WH
EH
CH
WHG
EH1
AAA
CH1
L1
EH2
YM
EH0
CHG
AAN
Anatolia_N
LB
YM1
CW
EHG
Nganasans
LBK_EN
Yamnaya_Samara
CCW
EstCWC
CWC
CCE
CCA
Hantis
N.
Mansis
O.
Russians_Central
P.
Nenets

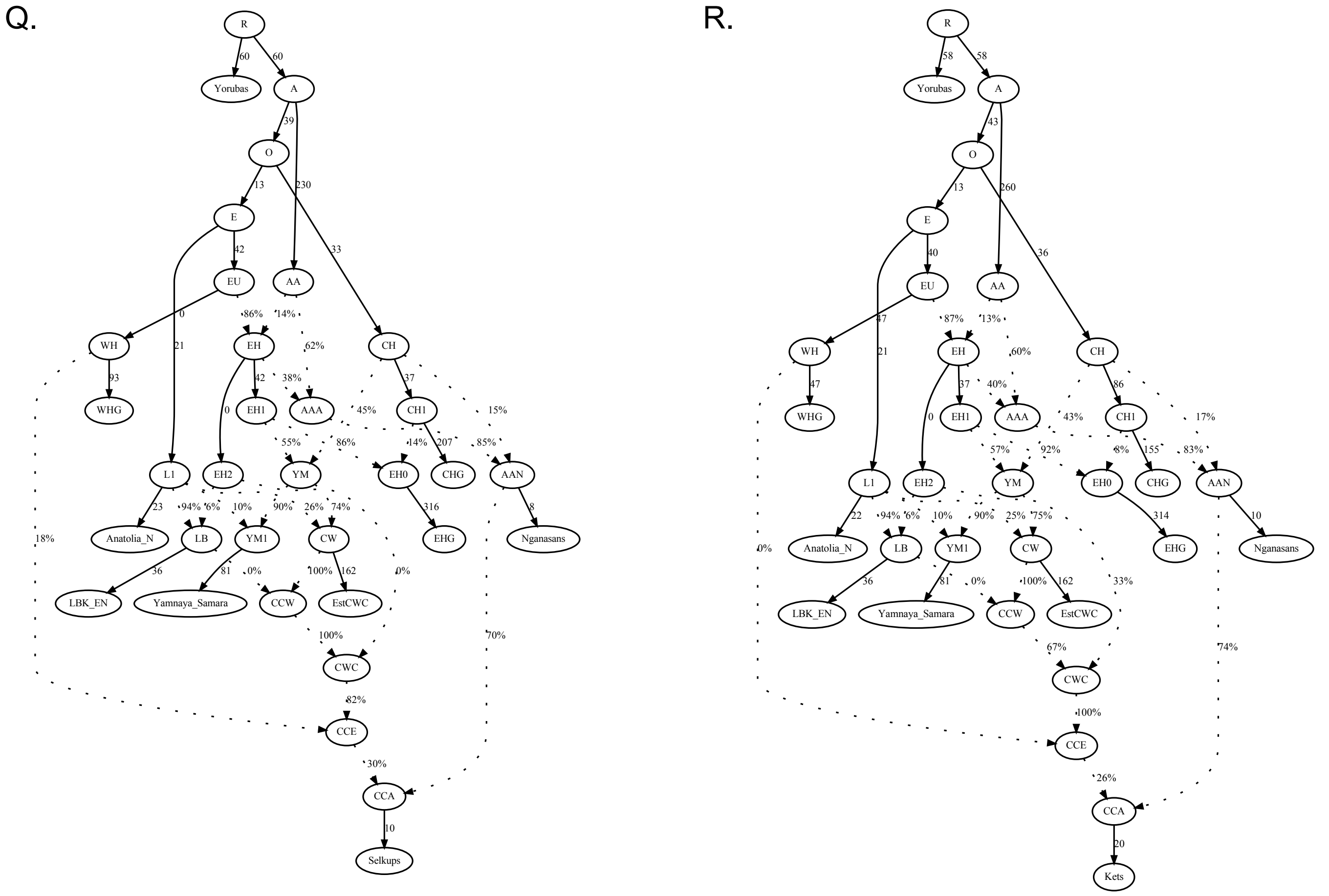

Figure S10. Admixture graphs of the demographic model with several admixture events (with shown % proportions), that fits tested data. **A.-B.**, **E.-N.** and **P.-Q.** – Uralic speaking populations; **C.-D.**, **O.** and **R.** – non-Uralic speaking populations.

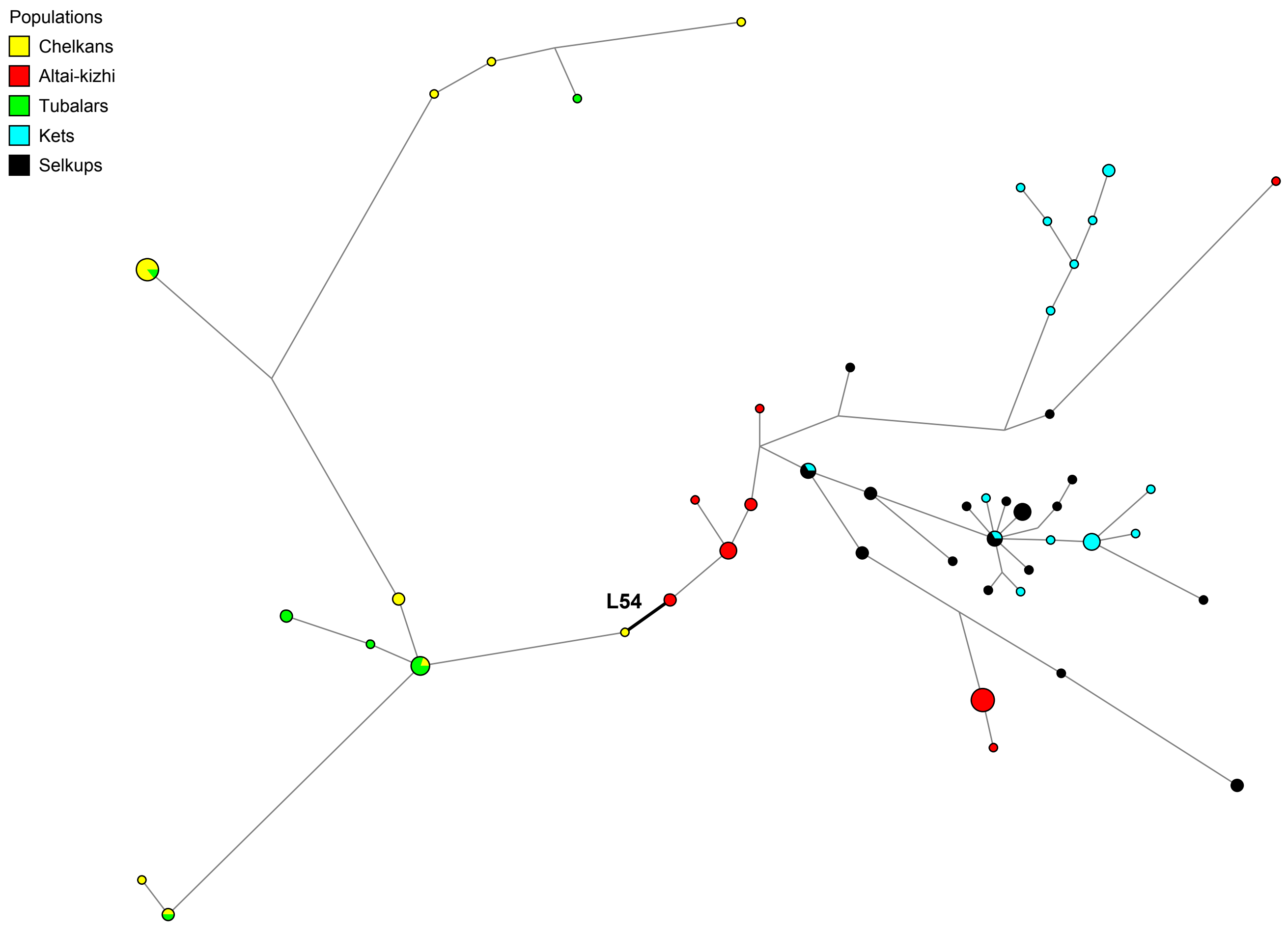

Figure S11. Phylogenetic network of Y chromosome haplogroup Q constructed from 17 Y chromosomal STRs with Network software version 2.0.0.1 (Fluxus-Engineering), applying median joining algorithm with a weight value of 90 for sub-clade defining SNP marker and a weight value of 10 for STR markers. A total of 88 samples (see legend for population data and Additional file 17: Table S16 for STR haplotypes) belonging to subhaplogroup Q1a3a were analyzed using the Y-Filer Kit and run on the ABI PRISM 3130*xl* Genetic Analyzer (Applied Biosystems). We tested all hg Q Y chromosomes of our sample of Selkups (n=17) and Kets (n=14) and found that all of these belong to the L54 defined branch of Q1a3a1, shown to be very common among Altai-kizhi populations of South Siberia.

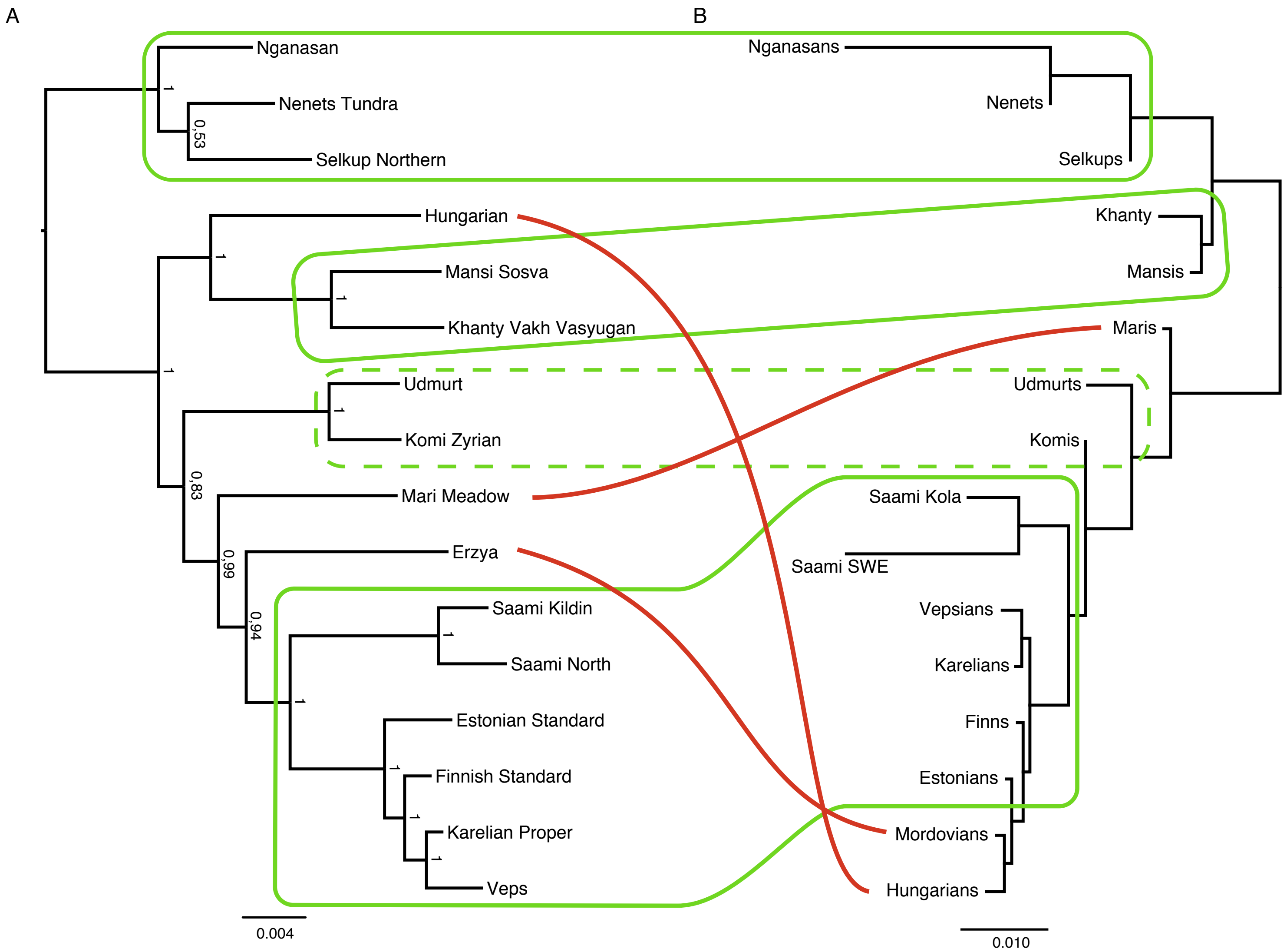

Figure S12. A. Quantitative phylogeny of 16 Uralic languages based on 226 basic vocabulary meanings coded by their cognacy relationships. Phylogeny is made with MrBayes by following the settings in Syrjänen et al. (2013). Shedding more light on language classification using basic vocabularies and phylogenetic methods. A case study of Uralic. Diachronica 30, 323–352. **B.** The evolutionary history of the Uralic speaking populations as inferred from pairwise Fst distance matrix using the Neighbor-Joining method [1]. The optimal tree with the sum of branch length = 0.13079639 is shown. The tree is drawn to scale, with branch lengths in the same units as those of the evolutionary distances used to infer the phylogenetic tree. Evolutionary analyses were conducted in MEGA7 [2]. Green and red cartoon overlaid on panels A and B highlights the concordances and disconcordances, respectively, of the two trees.

1. Saitou N. and Nei M. (1987). The neighbor-joining method: A new method for reconstructing phylogenetic trees. Molecular Biology and Evolution 4:406-425.
2. Kumar S., Stecher G., and Tamura K. (2016). MEGA7: Molecular Evolutionary Genetics Analysis version 7.0 for bigger datasets.Molecular Biology and Evolution 33:1870-1874.

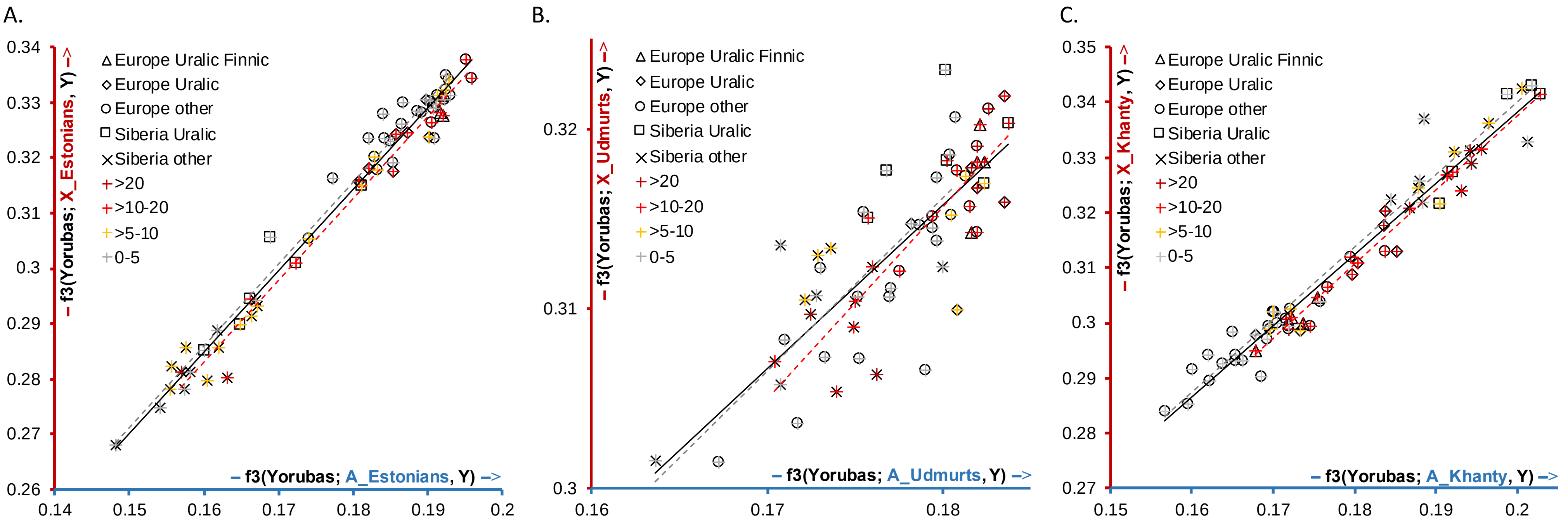

Figure S13. Comparison of autosomal (x-axis) and X chromosome (y-axis) outgroup f3-statistics for Estonians (A.), Udmurts (B.) and Khanty (C.). The population data is grouped by their geographic and linguistic (Uralic/non-Uralic) origins (see legend). Four classes of probability values (%) for a pair of men from two populations (Estonians, Udmurts or Khanty *vs* others) to share Y chromosome hg N3-M178 (calculated based on frequencies in Table S5) are shown with crosses of different colours. The overall trendline (black) and trendlines for lower (<5%, grey) and higher (>10%, red) probability classes are shown with dashed lines. Differences of slopes of the grey and red trendlines were non-significant in all tests (A. p=0.96; B. p=0.53; C. p=0.73). Differences of the interception points of the grey and red trendlines were significant for Estonians (p=0.01532) and Khanty (p=0.00542) but not for Udmurts (p=0.61670).
