# Supplementary material for: Genes reveal traces of common recent demographic history for most of the Uralic-speaking populations
Source: Genome Biol. 2018 Sep 21;19:139. doi: 10.1186/s13059-018-1522-1 (PMC6151024; doi:10.1186/s13059-018-1522-1)
Supplement: Supplementary file 12 — Table S11. Details of Globetrotter regional analysis. (PDF 50 kb) [file 13059_2018_1522_MOESM12_ESM.pdf]

Table S11. Details of regional Globetrotter (GT) analysis. In contrast to the 'full' analysis, where each recipient population was allowed to copy from all donor groups, in the 'regional' analysis recipients were allowed to copy only from a sub-set of possible donors. Populations, which are geographically and/or genetically (Figure S5) closely related to the target group of admixture, were excluded from the donor set. Note that West/Central Siberian group ("W-C-Sib") which also includes Nganassans, was split into three sub-groups for the GT analysis of Nganassan population – Nganassan, *Sib 1 and *Sib 2 (see Note below).

Region	Recipient population	Donor groups
Europe	Saami, Finnic, Europe1, Europe2	Bashkir, Cauc/N-East, N-Cauc/C-Asia, C-Asia/S-Siberia, Chuvash, E-Asia/S-Sib, Far_East, Komi, Khanty-Mansi, Mansi, Mari, N-Cauc/C-Asia, Samoyed, W-C-Sib, Tatar, Udmurt
Volga-Ural	Tatar, Udmurt, Chuvash, Komi, Mari	Bashkir, Cauc/N-East, N-Cauc/C-Asia, C-Asia/S-Sib, E-Asia/S-Sib, Europe1, Europe2, Far_East, Khanty-Mansi, N-Cauc/C-Asia, W-S-Europe, Saami, Samoyed, W-C-Sib, Finnic
Volga-Ural	Bashkir (see Note below)	Cauc/N-East, N-Cauc/C-Asia, C-Asia/S-Sib, Chuvash, E-Asia/S-Sib, Europe1, Europe2, Far_East, Komi, Khanty-Mansi, Mansi, Mari, N-Cauc/C-Asia, W-S-Europe, Saami, Samoyed, W-C-Sib, Tatar, Udmurt, Finnic
Siberia	Nganassan	Bashkir, Cauc/N-East, N-Cauc/C-Asia, C-Asia/S-Sib, Chuvash, E-Asia/S-Sib, Europe1, Europe2, Far_East, Komi, Khanty-Mansi, Mansi, Mari, N-Cauc/C-Asia, W-S-Europe, Saami, Samoyed, *Sib1, *Sib2, Tatar, Udmurt, Finnic
Siberia	Samoyed	Bashkir, Cauc/N-East, N-Cauc/C-Asia, C-Asia/S-Siberia, Chuvash, E-Asia/S-Sib, Europe1, Europe2, Far_East, Komi, Khanty-Mansi, Mansi, Mari, N-Cauc/C-Asia, W-S-Europe, Saami, W-C-Sib, Tatar, Udmurt, Finnic
Siberia	Khanty-Mansi	Bashkir, Cauc/N-East, N-Cauc/C-Asia, C-Asia/S-Sib, Chuvash, E-Asia/S-Siberia, Europe1, Europe2, Far_East, Komi, Mari, N-Cauc/C-Asia, W-S-Europe, Saami, Samoyed, W-C-Sib, Tatar, Udmurt, Finnic
Siberia	Mansi	Bashkir, Cauc/N-East, N-Cauc/C-Asia, C-Asia/S-Sib, E-Asia/S-Sib, Europe1, Europe2, Far_East, N-Cauc/C-Asia, W-S-Europe, Saami, Samoyed, W-C-Sib, Finnic

Note.

A) For the admixture analysis of all, except of the “Nganassan” clusters, the chromosome painting was performed using merged “W-C-Sib” cluster, which included the following clades from Figure S5):

6Evens;1Yakut
2Evens;1Yukaghir;1Dolgan
3Selkups
1Nganassan
8Nganassans
6Dolgans
4Evenkis;1Yakut;1Even;1Yukaghir
6Evenkis;1Dolgan
5Yakuts;3Evenkis
12Yakuts;1Evenkis

For the admixture analysis of “Nganassan” cluster, “W-C-Sib” was split into the following three groups (individual clade labels correspond to Figure S14):

“Nganassan”

3Selkups
1Nganassan
8Nganassans

“*Sib 1”

6Evens;1Yakut
2Evens;1Yukaghir;1Dolgan

“*Sib 2”

6Dolgans
4Evenkis;1Yakut;1Even;1Yukaghir
6Evenkis;1Dolgan
5Yakuts;3Evenkis
12Yakuts;1Evenkis

B) As Bashkirs cluster closely with Central Asian populations (Uzbeks and Turkmens), but not with other Volga-Ural groups, for the admixture analysis of “Bashkir” cluster copying was allowed from Tatars, Udmurts, Chuvashes, Komis and Maris.
